# Deep learning prediction of left atrial structure and function from 12-lead electrocardiograms

**DOI:** 10.1038/s41467-026-76155-6

**Published:** 2026-07-31

**Authors:** Jennifer A. Brody, Vidhushei Yogeswaran, Kerri L. Wiggins, Colleen M. Sitlani, Joshua C. Bis, Lin Yee Chen, Susan R. Heckbert, João A. C. Lima, W. T. Longstreth, Bruce M. Psaty, Elsayed Z. Soliman, Geoffrey H. Tison, Ting Ye, Ali Shojaie, James S. Floyd

**Affiliations:** 1 Cardiovascular Health Research Unit, Department of Medicine, University of Washington, Seattle, WA USA; 2 Lillehei Heart Institute and Department of Medicine (Cardiovascular Division), University of Minnesota Medical School, Minneapolis, MN USA; 3 Department of Epidemiology, University of Washington, Seattle, WA USA; 4 Cardiology Division, Department of Medicine, Johns Hopkins Hospital, Baltimore, MD USA; 5 Department of Neurology, University of Washington, Seattle, WA USA; 6 Department of Health Systems and Population Health, University of Washington, Seattle, WA USA; 7 Department of Cardiovascular Medicine, Wake Forest University School of Medicine, Winston-Salem, NC USA; 8 Division of Cardiology, University of California-San Francisco, San Francisco, CA USA; 9 Department of Biostatistics, University of Washington, Seattle, WA USA; 10 Department of Statistics, University of Washington, Seattle, WA USA

**Keywords:** Cardiovascular diseases, Risk factors, Machine learning

## Abstract

Abnormal cardiac atrial structure and function, termed atrial cardiopathy, typically precedes atrial fibrillation and downstream cardiovascular complications, yet detection is limited by the cost and accessibility of high-quality cardiac imaging. Here we show that a deep learning model trained on 12-lead electrocardiograms paired with 21,749 cardiac magnetic resonance scans from the UK Biobank predicts left atrial structure and function. Model-derived measures of atrial cardiopathy are strongly associated with new-onset atrial fibrillation, heart failure, and ischemic stroke after adjustment for clinical risk factors and biomarkers in two external cohorts, with magnitudes comparable to or greater than those for direct imaging measures and clinical risk factors. The risk of cardioembolic stroke, the hallmark complication of atrial fibrillation, increases 66% per standard deviation of left atrial volume. In exploratory analyses, model measures predict cardiac monitor-detected atrial fibrillation more accurately than a clinical risk prediction tool and NT-proBNP levels. This model is an inexpensive, accessible tool that identifies individuals at high-risk for atrial fibrillation and related complications.

## Abstract (web summary)

Atrial cardiopathy precedes atrial fibrillation and stroke, yet it is detected through costly, inaccessible cardiac imaging. Here, the authors show that a deep learning model estimates left atrial structure and function from a 12-lead ECG and independently predicts adverse cardiovascular outcomes.

## Introduction

Growing evidence suggests that abnormalities in the structure and function of the cardiac atria are independent risk factors for atrial fibrillation (AF), heart failure, and stroke, even among those without a clinical diagnosis of AF^1–3^. Most studies on atrial cardiopathy have focused on widely available electrocardiographic or echocardiographic measures of left atrial size, which have poor sensitivity for detecting atrial size and function^4–7^. Cardiac magnetic resonance imaging (CMR) is the gold-standard imaging modality for assessing atrial volume and function^8^, but is costly and not widely available in many areas^9,10^.

Recent studies have applied artificial intelligence (AI) and deep learning models to the resting 12-lead electrocardiogram (ECG), which has moderate accuracy in predicting short-and long-term risks of AF^11–14^. Building on the demonstrated success of deep learning approaches in predicting AF from ECG waveforms, we hypothesized that similar methods could extract information about underlying atrial structure and function when trained on paired ECG and imaging data. The clinical diagnosis of AF is influenced by the presence of symptoms and the duration of episodes, as well as characteristics that have little relation to biological risk, such as race^15,16^. This ascertainment bias fundamentally constrains the ability of models trained on clinically diagnosed AF to identify the underlying atrial substrate that predisposes to AF. The development and validation of models that predict left atrial and structure and function from ECG data has the potential to translate atrial cardiopathy into a tractable target for cardiovascular disease prevention, including application for population-wide screening for AF.

In this manuscript, we present a deep learning approach (ECG-AI) that reliably predicts left atrial volumes and ejection fraction from the resting 12-lead ECG. Our ECG-AI models, trained on high-quality CMR imaging data from over 20,000 participants and externally validated in 2 large population-based cohort studies, produced estimates that correlated only moderately with direct CMR-derived imaging measures yet were independently and robustly associated with new-onset AF, stroke, and heart failure after adjustment for traditional clinical risk factors. In an exploratory analysis of individuals who underwent 14-day cardiac monitoring 6 years after a screening 12-lead ECG, our ECG-AI measures predicted which individuals had cardiac monitor-detected AF with greater accuracy than an existing AF risk prediction tool and serum biomarker NT-proBNP. We also conducted genetic correlation analyses and model explainability studies to validate the cardiac specificity and explore the biological basis of our model’s predictions. This approach offers a translatable, low-cost method for assessing atrial size and function using the widely available ECG, facilitating the widespread screening of individuals who may be at high-risk for AF-related consequences across diverse clinical settings.

## Results

### ECG-AI model

**Fig. 1:**
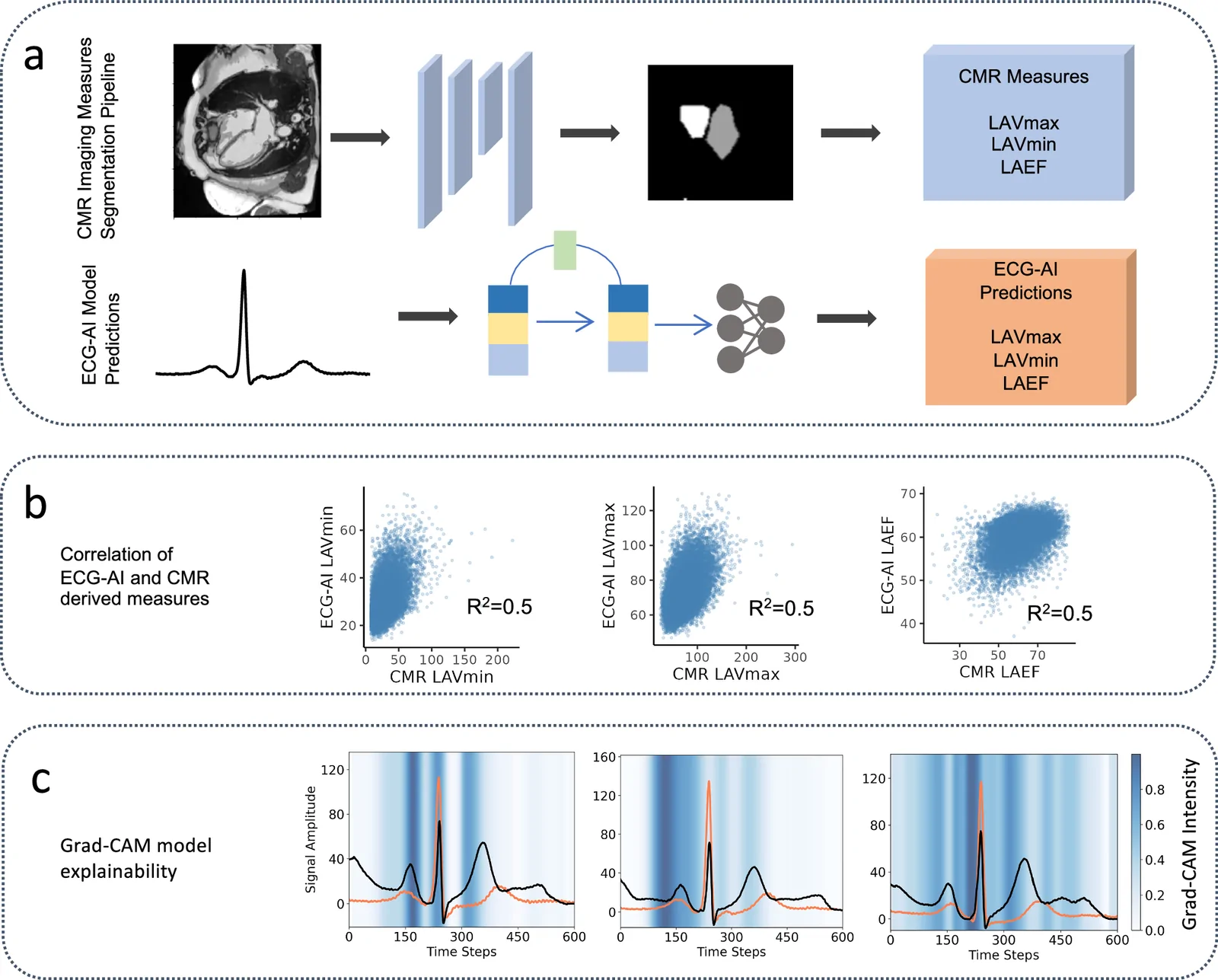
ECG-AI model development and evaluation. **a** Atrial volumes were derived from CMR images in UKB by applying a deep-learning model to segment the left atrial minimum and maximum volumes. We then trained our ECG-AI models to predict these volumes (LAVmin, LAVmax) and their proportion (LAEF) from 12-lead ECGs at the same study visit. **b** In the test partition of UKB, the Pearson correlations between the segmented CMR LA measure and ECG-AI predicted LA measure were 0.50 for LAVmin and LAVmax and 0.40 for LAEF. **c** Heatmaps for the ECG regions driving the predictions of the ECG-AI in UKB. The blue intensity corresponds to feature importance, with the darkest blue signaling the highest importance. The 50 largest volumes (worse) for LAVmin and the 50 smallest ejection fractions for LAEF (worse) median beats are averaged and displayed in orange. For comparison, the average of the 50 smallest (volumes) or largest (LAEF) are plotted in black. For LAVmin, the model is primarily focused on the decline of the P-wave, while LAVmax is focused on the first half of the P-wave. In contrast, the LAEF signal is focused on the PQ segment. Images reproduced by kind permission of UK Biobank ©.

We developed a deep learning model to predict left atrial imaging measures derived from CMR using digital 10-s 12-lead ECGs performed on the same day as the CMR imaging studies in the UK Biobank (UKB) participants. Left atrial minimal and maximal volumes (LAVmin, LAVmax) and total left atrial ejection fraction (LAEF) were extracted from CMR images using a validated machine-learning-based analysis pipeline^17^. All available unique participants with concurrent ECGs and CMR scans that passed QC (*N* = 48,300) were randomly split into 45% training, 5% validation, and 50% test samples (Supplementary Fig. 1). Participants in the training sample were 53% female, with a median age of 65 years, and a low prevalence of heart failure (0.4%) and prior myocardial infarction (2.1%) (Supplementary Tables 1 and 2). Participants in the test sample had similar characteristics. In the training sample, we used the vendor-generated median beat of the 8 non-derived leads of the 12-lead ECGs to train several models with a range of hyperparameters to predict LAVmin, LAVmax, and LAEF (Fig. 1). The validation sample was used for model selection and hyperparameter tuning. For each measure, predictions from the 5 highest-performing models were ensembled to create the final ECG-AI prediction model. Additional information on model architecture and training is in the methods, and final parameters are provided in Supplementary Table 3. The resulting ECG-AI model was then evaluated in the testing sample. The predicted (ECG-AI) LA measures were correlated with the imaging (CMR) measures in the testing partition: *r* = 0.50 for LAVmin and LAVmax and 0.40 for LAEF (Fig. 1). Absolute prediction errors represented 0.67–0.72 standard deviations of the variability in LA measurements. Mean absolute error in the test set was 8.6 mL for LAVmin (mean CMR value: 29.0 ± 12.8 mL), 14.8 mL for LAVmax (mean CMR value: 72.5 ± 21.7 mL), and 5.7 percentage points for LAEF (mean CMR value: 61.0 ± 7.9%). Root mean squared errors were 11.5 mL, 19.1 mL, and 7.3% points, respectively. These prediction errors are consistent with the observed correlations and indicate the model captured moderate inter-individual variation in LA parameters. Bland–Altman analyses showed no overall systematic bias (mean difference <1 mL for volumes, <1% for LAEF); however, proportional bias was evident, with the model tending to underestimate extreme values (regression toward the mean) (Supplementary Fig. 2).

### External validation: accuracy and representations

To test the transportability of our findings to other populations, we applied our fully trained ECG-AI model to resting 12-lead ECGs from the baseline examinations of 2 cohort studies: the Multi-Ethnic Study of Atherosclerosis (MESA) and the Cardiovascular Health Study (CHS). These studies provided both age and racial diversity for validation. MESA enrolled 6814 adults aged 45–84 years from 4 racial/ethnic groups (White, African American, Hispanic, and Chinese American), all free of clinical cardiovascular disease at enrollment in 2000–04. CHS is a study of 5888 adults aged 65 and older, recruited in 1989–90 and 1992–93, with an older mean age (73 years) compared to the UKB (65 years) and MESA (62 years) (Supplementary Table 2).

The correlations between LAVmin, LAVmax, and LAEF with ECG-AI predicted measures were lower than in UKB (Pearson’s *r* = 0.30–0.34; Supplementary Fig. 3). To better understand model transportability across cohorts, we analyzed learned representations from the model’s penultimate layer. t-SNE visualization of learned representations across all 3 prediction models showed CHS participants occupying predominantly distinct regions with scattered intermixing, while UKB and MESA overlapped extensively (Supplementary Fig. 4). Linear regression confirmed CHS participants occupied different embedding positions than UKB/MESA after accounting for age (*p* < 0.0001). Age-matched analysis (70–75 years) revealed CHS remained distinctly separated from UKB/MESA (distance: 37.6 units vs. 9.9 units between UKB age groups 10 years apart), indicating cohort-specific factors beyond age alone. Without labeled LA measurements in CHS, prediction accuracy cannot be directly assessed in this cohort.

### ECG-AI associations with clinical outcomes

In the UKB test sample, ECG-AI and CMR atrial measures were evaluated for associations with clinical outcomes. There were 331 AF, 71 ischemic stroke, and 118 heart failure events during a mean follow-up of 4 years. Left atrial volumes were indexed to body surface area (BSA) to obtain LAVImin and LAVImax. After adjustment for established clinical risk factors, CMR-derived LA measures were significantly associated with all outcomes (Table 1). Incident AF hazard ratios (HRs) for ECG-AI measures were slightly attenuated: each standard deviation (SD) higher (worse) CMR LAVImin was associated with a 44% increased risk (95% CI 34–53%), while ECG-AI-predicted LAVImin was associated with a 37% increased risk (95% CI 26–50%). In contrast, PR interval from the 12-lead ECG, an established risk factor for AF that reflects atrial and atrioventricular nodal conduction^18,19^, was not associated with AF (HR 1.01, 95% CI 0.91–1.12) or with other outcomes (*p* > 0.19) (Supplementary Table 4). Associations with ischemic stroke were not statistically significant in the UKB test sample (*n* = 71) and should be interpreted as directional only; substantive stroke inferences are drawn from MESA and CHS. ECG-AI measures were more potent risk factors for heart failure than the imaging measures themselves. For instance, the incident heart failure HR was 1.53 for ECG-AI LAVImax (95% CI 1.30–1.81, *P* = 3.64E-07) compared to only 1.36 (95% CI 1.20–1.55, *P* = 1.23E-06) for CMR LAVImax.

**Table 1:** Associations of CMR and ECG-AI predicted measures of left atrial function with outcomes in the UK Biobank study

		CMR		ECG-AI	
	Events/total *N*	HR (95% CI)	*P* value	HR (95% CI)	*P* value
Atrial fibrillation					
LAVImin	331/18,804	1.44 (1.34–1.53)	5.93E-27	1.37 (1.26–1.50)	2.79E-12
LAVImax	331/18,804	1.37 (1.25–1.50)	4.72E-12	1.31 (1.17–1.46)	1.70E-06
LAEF	331/18,804	0.63 (0.57–0.69)	9.38E-20	0.76 (0.69–0.82)	2.33E-10
Ischemic stroke					
LAVImin	71/16,347	1.37 (1.21–1.56)	1.12E-06	1.16 (0.95–1.43)	0.15
LAVImax	71/16,347	1.38 (1.15–1.64)	3.69E-04	1.10 (0.87–1.39)	0.42
LAEF	71/16,347	0.64 (0.52–0.79)	3.80E-05	0.89 (0.72–1.09)	0.24
Heart failure					
LAVImin	118/16,352	1.26 (1.14–1.39)	7.82E-06	1.42 (1.24–1.63)	6.46E-07
LAVImax	118/16,352	1.36 (1.20–1.55)	1.23E-06	1.53 (1.30–1.81)	3.64E-07
LAEF	118/16,352	0.75 (0.64–0.88)	3.49E-04	0.74 (0.64–0.85)	2.17E-05

Hazard ratios (HR) with 95% confidence intervals (CI) are reported per standard deviation increase in each left atrial measure in the UK Biobank cohort, with direct CMR measurements on the left and ECG-AI predictions on the right for comparison. HRs were derived from Cox proportional hazards regression with two-sided *p*-values; no adjustment for multiple comparisons was made. Models were adjusted for age, sex, race, body mass index, systolic blood pressure, current smoking, antihypertensive medication use, and diabetes mellitus. AF and ischemic stroke analyses were additionally adjusted for heart failure and myocardial infarction, and AF analyses were additionally adjusted for diastolic blood pressure. Heart failure analyses were additionally adjusted for total cholesterol, high-density lipoprotein cholesterol, and left ventricular ejection fraction.

### External validation of outcome associations: MESA and CHS

ECG-AI associations with cardiovascular outcomes, after adjustment for established clinical risk factors, were similar or greater in magnitude in MESA (mean follow-up 13.6 years) and CHS (mean follow-up 11.6 years). For incident AF, risk estimates were similar across all 3 cohorts. Each SD higher (worse) ECG-AI predicted LAVImin was associated with greater risk: HR 1.37 (95% CI 1.26–1.50, *P* = 2.79E-12) in the UKB test sample, HR 1.34 (95% CI 1.28–1.41, *P* = 2.58E-30) in MESA, and HR 1.41 (95% CI 1.35–1.47, *P* = 3.04E-49) in CHS. In contrast, each SD higher (better) ECG-AI predicted LAEF was associated with a HR of 0.76 (95% CI 0.69–0.82, *P* = 2.33E-10) in the UKB test sample, HR 0.76 (95% CI 0.73–0.80, *P* = 8.10E-27) in MESA, and HR 0.72 (95% CI 0.69–0.75, *P* = 8.57E-56) in CHS (Tables 1 and 2).

**Table 2:** Associations of ECG-AI predicted measures and biomarkers of left atrial function with clinical outcomes in the Multi-Ethnic Study of Atherosclerosis and the Cardiovascular Health Study

	MESA			CHS		
	Event/*N*	HR (95% CI)	*P* value	Event/*N*	HR (95% CI)	*P* value
Atrial fibrillation						
ECG-AI LAVImin	1298/6677	1.34 (1.28–1.41)	2.58E-30	2232/5154	1.41 (1.35–1.47)	3.04E-49
ECG-AI LAVImax	1298/6677	1.34 (1.26–1.42)	5.23E-20	2232/5154	1.26 (1.20–1.33)	1.85E-19
ECG-AI LAEF	1298/6677	0.76 (0.73–0.80)	8.10E-27	2232/5154	0.72 (0.69–0.75)	8.57E-56
LA strain	771/4153	0.79 (0.72–0.86)	4.32E-08	1644/3632	0.81 (0.77–0.86)	4.63E-14
ECG P TFV1	1298/6677	1.13 (1.07–1.19)	1.73E-05	2232/5154	1.03 (0.99–1.08)	1.61E-01
Ischemic stroke						
ECG-AI LAVImin	253/5637	1.16 (1.03–1.30)	0.01	777/4940	1.22 (1.13–1.32)	1.88E-07
ECG-AI LAVImax	253/5637	1.16 (1.02–1.33)	0.03	777/4940	1.17 (1.08–1.27)	8.65E-05
ECG-AI LAEF	253/5637	0.87 (0.78–0.98)	0.02	777/4940	0.82 (0.76–0.88)	2.21E-08
LA strain	168/3525	1.03 (0.88–1.20)	0.73	555/3510	0.84 (0.76–0.92)	1.89E-04
ECG PTFV1	253/5637	1.13 (1.00–1.27)	0.05	777/4940	1.13 (1.05–1.21)	8.17E-04
Heart failure						
ECG-AI LAVImin	244/4190	1.36 (1.23–1.50)	4.79E-09	1250/4392	1.45 (1.37–1.53)	6.68E-37
ECG-AI LAVImax	244/4190	1.40 (1.23–1.58)	9.96E-08	1250/4392	1.37 (1.29–1.45)	5.47E-24
ECG-AI LAEF	244/4190	0.71 (0.64–0.79)	3.61E-10	1250/4392	0.74 (0.70–0.78)	4.61E-28
LA strain	216/3527	0.91 (0.78–1.05)	0.2	1011/3506	0.79 (0.74–0.85)	3.91E-11
ECG PTFV1	244/4190	1.26 (1.11–1.43)	2.45E-04	1250/4392	1.10 (1.04–1.17)	7.36E-04

Hazard ratios (HR) with 95% confidence intervals (CI) are reported per standard deviation increase in each left atrial measure in the Multi-Ethnic Study of Atherosclerosis (MESA) and Cardiovascular Health Study (CHS). Results are shown for ECG-AI predicted measures (LAVImin, LAVImax, and LAEF), LA strain measured by cardiac magnetic resonance imaging (CMR) in MESA and echocardiography (Echo) in CHS, and PTFV1 derived from 12-lead ECG. HRs were derived from Cox proportional hazards regression with two-sided *p*-values; no adjustment for multiple comparisons was made. Models were adjusted for age, sex, race, body mass index, systolic blood pressure, current smoking, antihypertensive medication use, and diabetes mellitus. AF and ischemic stroke analyses were additionally adjusted for heart failure and myocardial infarction, and AF analyses were additionally adjusted for diastolic blood pressure. Heart failure analyses were additionally adjusted for total cholesterol, high-density lipoprotein cholesterol, and left ventricular ejection fraction.

Associations of ECG-AI measures with incident ischemic stroke were weaker but statistically significant: HRs for LAVImin were 1.16 in the UKB test sample (95% CI 0.95–1.43, *P* = 0.15), 1.16 in MESA (95% CI 1.03–1.30, *P* = 0.01), and 1.22 in CHS (95% CI 1.13–1.32, *P* = 1.88E-7). We observed the strongest associations for heart failure: each SD higher ECG-AI LAVImin was associated with HRs of 1.42 in the UKB test sample (95% CI 1.24–1.63, *P* = 6.46E-7), 1.36 in MESA (95% CI 1.23–1.50, *P* = 4.79E-9), and 1.45 in CHS (95% CI 1.37–1.53, *P* = 6.68E-37). In MESA and CHS, some measures showed evidence of non-proportionality over the extended follow-up period; sensitivity analyses restricted to 5-year follow-up showed stronger associations and largely resolved the proportional hazards violations (Supplementary Data 1).

Given that CHS enrolled exclusively adults aged ≥65, we performed age-stratified analyses in MESA to assess whether associations differed by age (Supplementary Data 2). ECG-AI associations with AF and heart failure were robust in both younger (<65 years) and older (≥65 years) MESA participants, with AF associations somewhat stronger in older participants (LAVImin HR 1.40 vs. 1.27), consistent with the pattern observed in CHS. Heart failure associations were similarly robust across age groups, with point estimates somewhat larger in younger participants (LAVImin HR 1.46 vs. 1.35). Ischemic stroke associations were directionally consistent but not statistically significant in either age group, likely reflecting limited event numbers.

Sex-stratified analyses in MESA and CHS demonstrated consistent ECG-AI associations with AF and heart failure across both females and males (Supplementary Data 3). For AF, LAVImin hazard ratios ranged from 1.32 to 1.48 in females and 1.32 to 1.34 in males, with highly significant associations in all strata (all *P* < 10E-13). Heart failure associations were similarly robust, with LAVImin hazard ratios of 1.34–1.43 in females and 1.38–1.46 in males (all *P* < 10E-4). However, ischemic stroke associations appeared stronger in females than males in CHS, where female-specific associations were highly significant (LAVImin HR 1.31, *P* = 3.84 × 10E-9) while male-specific associations were not significant (HR 1.06, *P* = 0.38).

### Comparison of ECG-AI with atrial cardiopathy measures

To place these findings in context with existing, widely studied measures of left atrial cardiopathy, we evaluated P-wave terminal force in lead V1 (PTFV1) from the 12-lead ECG and LA reservoir strain from CMR in MESA and echocardiography in CHS^20^. Associations for ECG-AI measures were much stronger than for PTFV1 and were similar to or stronger than LA reservoir strain associations for all 3 outcomes (Table 2). In MESA, LA parameters measured directly from CMR demonstrated slightly stronger associations with outcomes than their ECG-AI predicted counterparts (Supplementary Data 4).

In MESA, we also evaluated correlations between LA parameters with other ECG and imaging measures of cardiac structure and function. The correlations of both the ECG-AI predicted and CMR-measured LA parameters with left ventricular ejection fraction (LVEF) and with PTFV1 were low, with all Pearson correlations below |0.2| (Supplementary Table 5). Correlations with log NT-proBNP, a sensitive but nonspecific protein biomarker of ventricular stretch associated with several cardiovascular outcomes, were stronger for ECG-AI measures (|*r*| = 0.33–0.37) than for CMR measures (|*r*| = 0.16–0.28).

Given that the widely available clinical biomarker NT-proBNP is a strong predictor of AF cardioembolic stroke and heart failure (Supplementary Table 6), and is moderately correlated with LA ECG-AI measures, we additionally adjusted for log NT-proBNP. Associations between ECG-AI measures adjusted for log NT-proBNP with AF, ischemic stroke, and heart failure were partly attenuated, but remained significantly associated with outcomes (Supplementary Table 7).

### Ischemic stroke subtypes

Most ischemic strokes can be classified by their likely causative mechanisms based on neuroimaging and clinical context. Large-artery atherosclerotic (previously called atherothrombotic), small-vessel (i.e., lacunar strokes), and cardioembolic are the most common etiologies^21^. ECG-AI measures were strongly associated with cardioembolic stroke, the hallmark complication of both AF and atrial cardiopathy^22^, but not large-artery atherosclerotic or small-vessel strokes, as hypothesized. For example, each SD higher ECG-AI LAVImin was associated with a 38% greater risk of cardioembolic stroke in MESA (95% CI 14–69%, *P* = 1.27E-03) and 66% greater risk in CHS (95% CI 47–86%, *P* = 3.67E-17), while each SD higher ECG-AI LAEF was associated with a 30% decreased risk of cardioembolic stroke in MESA (95% CI 16–42%, *P* = 2.01E-04) and a 41% decreased risk in CHS (95% CI 34–47%, *P* = 1.49E-20) (Table 3). Conversely, none of the ECG-AI measures were associated with small vessel or large artery atherosclerotic stroke (*P* > = 0.37).

**Table 3:** Associations of left atrial function measures with stroke subtypes in the Multi-Ethnic Study of Atherosclerosis and the Cardiovascular Health Study

		MESA				CHS	
	Event/*N*	HR (95% CI)	*P* value		Event/*N*	HR (95% CI)	*P* value
Cardioembolic							
ECG-AI LAVImin	75/5637	1.38 (1.14–1.69)	1.27E-03		246/4940	1.66 (1.47–1.86)	3.67E-17
ECG-AI LAVImax	75/5637	1.24 (0.96–1.59)	0.10		246/4940	1.47 (1.29–1.67)	3.71E-09
ECG-AI LAEF	75/5637	0.70 (0.58–0.84)	2.01E-04		246/4940	0.59 (0.53–0.66)	1.49E-20
LA strain	49/3525	0.84 (0.60–1.17)	0.30		189/3510	0.67 (0.57–0.80)	3.98E-06
ECG PTFV1	75/5637	1.25 (1.02–1.54)	0.03		246/4940	1.21 (1.07–1.36)	2.22E-03
Small vessel							
ECG-AI LAVImin	71/5637	1.10 (0.88–1.38)	0.40		132/4940	1.03 (0.85–1.26)	0.73
ECG-AI LAVImax	71/5637	1.12 (0.87–1.44)	0.39		132/4940	1.06 (0.87–1.30)	0.54
ECG-AI LAEF	71/5637	0.91 (0.72–1.13)	0.38		132/4940	0.99 (0.83–1.19)	0.94
LA strain	47/3525	1.25 (0.97–1.61)	0.09		101/3510	1.00 (0.81–1.23)	0.99
ECG PTFV1	71/5637	1.14 (0.91–1.43)	0.25		132/4940	1.22 (1.03–1.43)	0.02
Large-artery atherosclerotic							
ECG-AI LAVImin	43/5637	0.88 (0.63–1.24)	0.46		55/4940	1.07 (0.78–1.47)	0.69
ECG-AI LAVImax	43/5637	0.84 (0.58–1.22)	0.37		55/4940	1.08 (0.78–1.51)	0.63
ECG-AI LAEF	43/5637	1.04 (0.76–1.43)	0.79		55/4940	0.90 (0.68–1.19)	0.46
LA strain	28/3525	1.18 (0.85–1.65)	0.32		41/3510	0.97 (0.71–1.34)	0.88
ECG PTFV1	43/5637	0.86 (0.61–1.21)	0.38		55/4940	1.02 (0.76–1.36)	0.91

Hazard ratios (HR) with 95% confidence intervals (CI) are reported for each stroke subtype per standard deviation increase in each left atrial measure across the Multi-Ethnic Study of Atherosclerosis (MESA) and Cardiovascular Health Study (CHS). Results are shown for ECG-AI predicted measures (LAVImin, LAVImax, and LAEF), LA strain measured by cardiac magnetic resonance imaging (CMR) in MESA and echocardiography (Echo) in CHS, and PTFV1 derived from 12-lead ECG. HRs were derived from Cox proportional hazards regression with two-sided *p*-values; no adjustment for multiple comparisons was made. Models were adjusted for age, sex, race, BMI, SBP, current smoking, antihypertensive medication use, DM, prevalent heart failure, and MI.

### Clinical prediction models for AF and cardioembolic stroke

Beyond evaluating prognostic associations, we assessed whether ECG-AI measures could improve clinical risk prediction when incorporated into prediction models. We developed 5-year prediction models for each outcome, comparing four approaches: (1) base model (age and sex only), (2) base model and a weighted linear combination of the 3 ECG-AI measures, (3) CHARGE-AF clinical risk score, and (4) CHARGE-AF and ECG-AI measures. Model weights were estimated in UKB and externally validated in MESA and CHS. There were 842 AF events and 63 cardioembolic strokes across MESA and CHS.

**Fig. 2:**
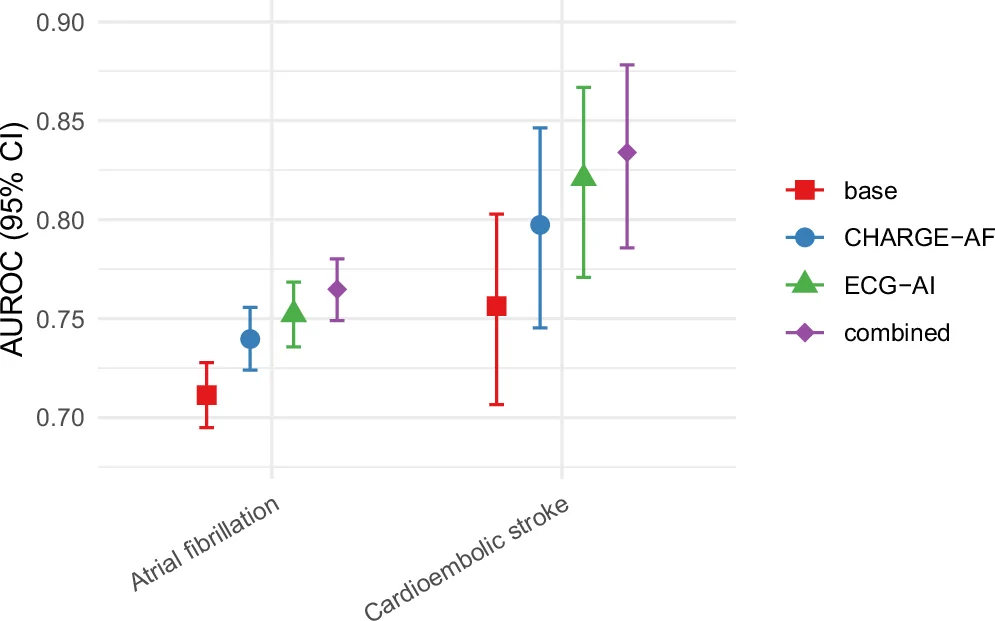
Classification performance of 5-year AF risk scores. Receiver operating characteristic area under the curve (AUC) for 5-year AF risk score classification performance in combined MESA and CHS cohorts. Analyses included 842 participants with incident AF and 10,004 without, and 63 participants with cardioembolic stroke and 1059 without. The prediction models compared are: (1) age and sex only (base), (2) base + ECG-AI left atrial measures (ECG-AI), (3) CHARGE-AF risk score alone (CHARGE-AF), and (4) CHARGE-AF + ECG-AI (combined). Models were evaluated for 5-year risk of AF and cardioembolic stroke. Plots show AUC point estimates with 95% bootstrap confidence intervals as error bars. Models are distinguished by color and shape: base (red square), CHARGE-AF (blue circle), ECG-AI (green triangle), and combined (purple diamond).

For 5-year AF prediction (Fig. 2 and Supplementary Table 8), the ECG-AI model performed similarly to the CHARGE-AF model in terms of area under the received operating characteristic curve (AUROC, 0.75 vs. 0.74) but had better precision-recall characteristics (AUPRC, 0.23 vs. 0.18). Adding ECG-AI to CHARGE-AF resulted in statistically significant but modest improvements in discrimination (Δ AUROC 0.024, 95% CI 0.017–0.034, *P* < 10E-4) and AUPRC (Δ AUPRC 0.046, 95% CI 0.030–0.063, *P* < 10E-4). Decision curve analysis showed that ECG-AI and the combined model provided greater net benefit than CHARGE-AF across clinically relevant risk thresholds (Supplementary Fig. 5).

For the prediction of cardioembolic stroke, ECG-AI improved discrimination compared with CHARGE-AF (AUROC 0.82 vs. 0.80; AUPRC 0.033 vs. 0.026), and the addition of ECG-AI to CHARGE-AF improved prediction further (Δ AUROC 0.037, 95% CI 0.008–0.066, *P* = 0.012). Net benefit differences for cardioembolic stroke were minimal. When categorizing risk predictions into tertiles, the high-risk ECG-AI group has an OR of 52.8 (95% CI 7.3–382, *P* = 8.5E-5) compared to the low-risk group.

### Screening for AF with 14-day cardiac monitoring: an exploratory analysis

AF can be difficult to detect because it is paroxysmal and asymptomatic in many individuals. One potential application for a tool that estimates LA function is the selection of high-risk individuals who might benefit from AF detection (i.e., screening) with extended cardiac monitoring, a sensitive and unbiased method of arrhythmia detection^23^. In MESA, a resting 12-lead ECG was performed at Exam 5 (2010–12) and 14-day ambulatory cardiac monitoring was performed an average of 6 years later at Exam 6 (2016–18) in more than 1500 participants. We applied our ECG-AI model to Exam 5 ECGs and identified participants in the highest risk quintile of each ECG-AI measure, the CHARGE-AF score, and serum NT-proBNP levels. Out of 82 participants with monitor-detected AF, 69 had no previous clinically detected AF at the time of their Exam 5 12-lead ECG. The highest-risk quintile of each ECG-AI measure predicted monitor-detected AF with greater accuracy than CHARGE-AF (AUROC 0.58, 95% CI 0.53–0.63) and NT-proBNP (AUROC 0.62, 95% CI 0.56–0.68). For instance, using the lowest quintile of ECG-AI LAEF as the risk threshold resulted in an AUROC of 0.66 (95% CI 0.60–0.71), a positive predictive value of 14%, and identified half of all cases of monitor-detected AF. (Supplementary Table 9). When comparing the high-risk quintile to the lowest 4 quintiles, the relative risk for cardiac monitoring-detected AF with CHARGE-AF was 2.3 (95% CI 1.4–3.7), and for ECG-AI LAEF it was 4.5 (95% CI 2.9–7.1).

### Model interpretability and generalizability

To identify which ECG features most influenced our model predictions, we analyzed gradient-weighted class activation mapping (Grad-CAM) intensities for the 50 highest-risk ECGs (those predicting the largest LA volumes and lowest LAEF values) (Fig. 1 and Supplementary Fig. 6). For predicting LAVmin volumes, the model primarily focused on the second half of the P wave, with a secondary focus on the R peak and the ascending portion of the T wave. For LAVmax predictions, the model was broadly focused on the entire P-waveform, with an emphasis on the first half of the P wave. When predicting lower LAEF, the influential regions spanned more broadly across the P-QRS waveforms but were most pronounced over the PQ segment. These patterns were consistent across cohorts.

Systematic ablation experiments validated these saliency maps (Supplementary Fig. 7). Removing Grad-CAM high-importance regions caused substantially greater performance degradation than random ablation, confirming the functional importance of identified features.

### Genetic analyses of ECG-AI phenotypes

To uncover some of the biological factors driving our ECG-AI measures, we used human genomic data. We performed genome-wide association study (GWAS) analyses of 5 ECG-AI measures (indexed and non-indexed volumes and LAEF) among European ancestry participants in the UK Biobank study test sample who were free of AF at baseline (*N* = 33,245) (Supplementary Fig. 1). We identified 12 loci across the 5 traits (Supplementary Data 5 and Supplementary Fig. 8). The strongest association was observed for the T allele of rs11153730 (coded allele frequency 0.51, beta = − 0.085, SE = 0.008, *P* = 8.1E-27), a well-characterized variant near *PLN* that is a locus for multiple ECG metrics, heart failure, and AF. The estimated heritability (*h*^2^) of ECG-AI traits was modest, ranging from 0.14 for LAVmax to 0.17 for LAVImax (Supplementary Table 10). Notably, half of these loci do not have genomic targets that overlap with AF loci^24^.

**Fig. 3:**
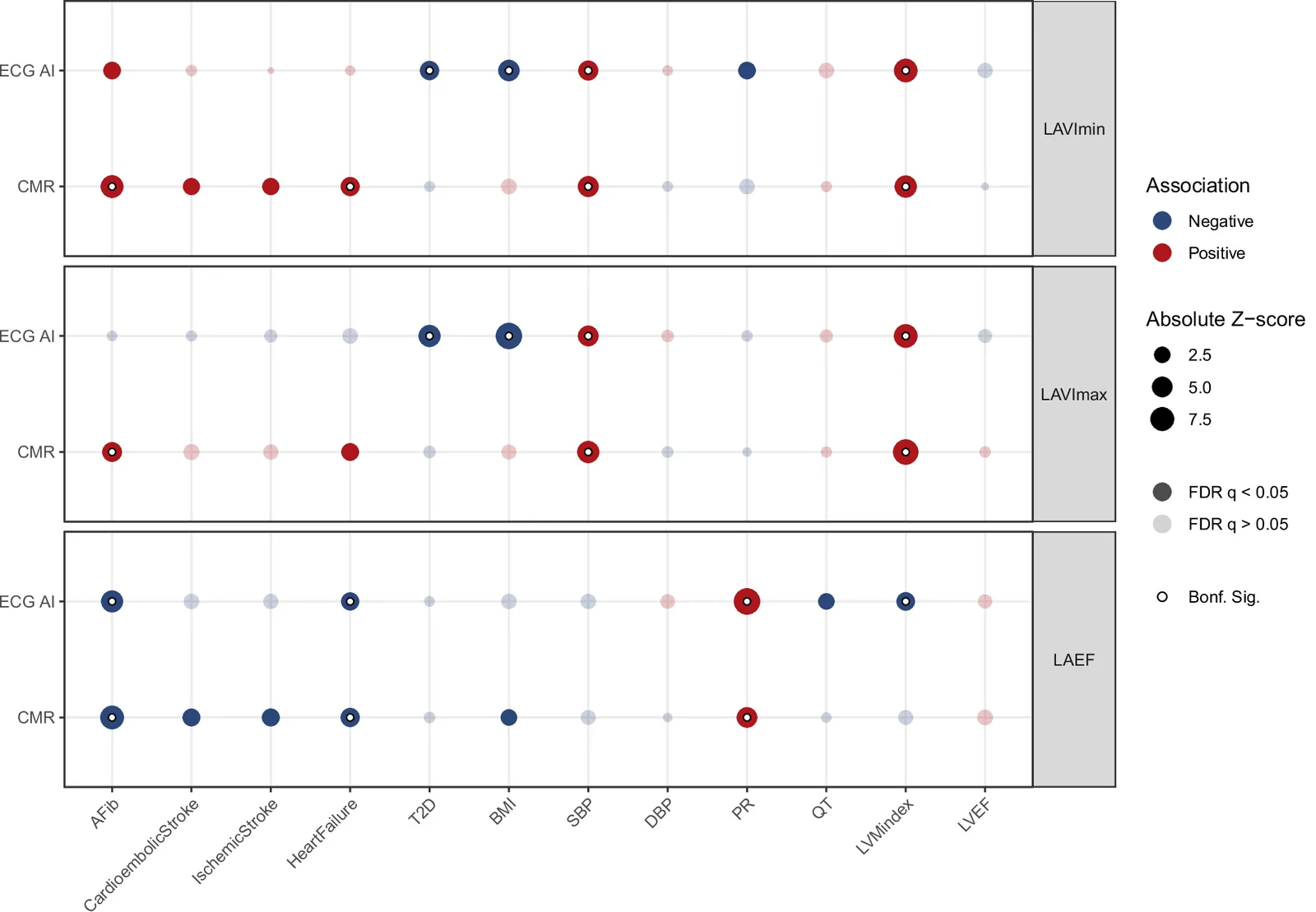
Genetic correlation of ECG-AI and CMR atrial measures with cardiovascular traits. LD score regression shows the genetic correlation between ECG-AI prediction measures, directly measured CMR traits, and cardiovascular traits. Each panel corresponds to a different left atrial parameter: left atrial ejection fraction (LAEF), left atrial maximal volume indexed to BSA (LAVImax), and left atrial minimal volume indexed to BSA (LAVImin). The size of the bubble represents the absolute Z-score for each trait. Positive cross-trait associations are in red, while negative associations are in blue. Associations with a significant correlation after Bonferroni correction contain a white core, and those with a significant correlation after controlling for the false discovery rate (FDR) are saturated (dark), while insignificant correlations are in greyscale.

Using these GWAS results, we evaluated genetic correlations between ECG-AI predictions, CMR measurements, and established cardiovascular risk factors. ECG-AI measures demonstrated moderate genetic correlation with their CMR counterparts: *h*^2^ of 0.47 for LAVImin, 0.34 for LAVImax, and 0.62 for LAEF. We found left ventricular mass (LVM) indexed to BSA had a strong positive genetic correlation with ECG-AI volume measures and was negatively correlated with LAEF (Fig. 3). LAEF was also genetically correlated with AF and heart failure, but the volume measures were not. The ECG-AI volume measures exhibited genetic correlations with type 2 diabetes and body mass index that were not present with the directly measured CMR volume traits.

## Discussion

We have demonstrated that deep learning models trained on high-quality cardiac magnetic resonance imaging can predict left atrial size and function from the resting 12-lead ECG, an inexpensive and widely available test. Our ECG-AI measures were strongly associated with incident AF, incident heart failure, and incident ischemic stroke, independent of established risk factors and biomarkers for these outcomes, with remarkable specificity for the cardioembolic stroke subtype. Our ECG-AI measures demonstrated prognostic associations with cardiovascular outcomes that were comparable to or, in some cases, stronger than conventional ECG and imaging measures of atrial cardiopathy. Human genetic evidence revealed modest genetic correlation with CMR measures (*h*^2^ = 0.34–0.62) and some overlap in genomic loci with AF, suggesting the ECG-AI measures and direct imaging share some but not all biological determinants of cardiovascular risk. The consistent performance of these ECG-AI measures in 2 external cohort studies using different ECG acquisition systems and requiring only voltage rescaling without additional model training, calibration, or cohort-specific preprocessing demonstrates the reproducibility and transportability of the ECG-AI outcome associations across diverse populations, though formal fairness assessment remains necessary to ensure responsible implementation. Moreover, compared to a clinical risk prediction tool, our ECG-AI improved the 5-year prediction of AF and cardioembolic stroke, the hallmark complication of AF, and exploratory analyses found that ECG-AI identified nearly half of individuals with cardiac monitoring-detected AF.

Previous deep learning approaches applied to ECG analysis have primarily focused on predicting the risk of clinically identified outcomes. For instance, Attia et al.^12^ demonstrated that AI-enabled ECG could identify AF from ECGs in sinus rhythm after or within 31 days of diagnosis, with an AUC of 0.87. Similarly, Raghunath et al.^14^ developed an ECG-AI model to detect paroxysmal AF with comparable performance. These classification models effectively served as “black boxes,” identifying patterns associated with AF without explicitly modeling the underlying pathophysiological changes. There have been numerous highly capable ECG models developed for arrhythmia detection^25^, with some achieving physician-level performance but providing limited insight into structural cardiac abnormalities preceding arrhythmogenesis and biological mechanisms^26^. These models are inherently limited by their reliance on a clinical diagnosis of AF, which is present but undetected in a large proportion of individuals at a rate that varies by race^16,27^.

In contrast to these binary classification approaches, our research represents a fundamental shift toward quantitative prediction of continuous LA parameters that reflect the substrate for AF development. While previous models asked, “Does this patient have AF?” our model asks, “What is the state of this patient’s left atrium?” This latter approach may offer advantages. First, by directly modeling LA volumes and function, established imaging biomarkers with known pathophysiological relevance, our predictions provide mechanistic insights into the atrial substrate. Second, our model outputs continuous estimates of cardiac structure and function, potentially enabling more granular risk stratification than binary classification. Third, by directly predicting atrial volume and function, we avoid the ascertainment bias inherent in models that rely on clinically detected AF. Fourth, the moderate genetic correlation between our ECG-AI and CMR measures (*h*^2^ of 0.47–0.62), coupled with identification of genetic loci shared with AF, provides biological validation lacking in previous classification models. Lastly, atrial cardiopathy itself is a target for disease prevention and clinical trials of therapeutics are already underway^28–30^; an inexpensive and widely available tool for the detection of atrial cardiopathy could accelerate the development of new therapeutic approaches to reduce atrial cardiopathy-related complications.

A notable finding was the specificity of ECG-AI measures for cardioembolic stroke and not for other stroke subtypes. This specificity aligns with the pathophysiological mechanisms linking atrial dysfunction to thromboembolism. Stroke subtypes were rigorously adjudicated in the cohort studies, which made this comparison possible. Our findings support the use of ECG-AI measures in risk-stratification for cardioembolic stroke, a condition that often requires different preventive strategies than other stroke subtypes.

Our Grad-CAM analyses revealed mechanistic insight into the ECG features most informative for predicting left atrial measures, with the ECG-AI model focused on different regions of the cardiac cycle for each left atrial measure. The LAVmin model was focused on the second half of the P wave for LAVmin, while the LAVmax model was focused on the first half. In contrast, the LAEF signal was focused during atrioventricular repolarization on the PQ segment, suggesting that our model captures physiological signals consistent with our understanding of cardiac electrophysiology. Ablation analyses confirmed that these regions were functionally important for model predictions.

Although correlations are only moderate, ECG-AI has strong associations with clinical outcomes and captures biologically meaningful information for cardiovascular risk stratification. CMR provides a direct anatomic and functional assessment, while the ECG reflects atrial depolarization and repolarization, and captures other downstream aspects of remodeling, loading conditions, and conduction. To assess whether ECG-AI predictions captured cardiac-specific biology beyond general risk factors, such as age, sex, and BMI, which ECG signals predict well, we performed genetic analyses. The moderate genetic correlations between ECG-AI and CMR measures (*h*^2^ of 0.47 for LAVImin, 0.34 for LAVImax, and 0.62 for LAEF) suggest that our model captures a substantial portion of the heritable component of left atrial structure and function. However, the genetic correlations between our ECG-AI measures and related risk factors and outcomes differed from directly measured CMR variables. The identification of 12 genetic loci, including the well-characterized PLN variant associated with heart failure and AF, supports the cardiac specificity of our ECG-AI measures. These genetic findings support that ECG-AI captures biologically meaningful cardiac information and provide a foundation for future mechanistic studies.

As in previous studies^31,32^, we found that NT-proBNP was a potent risk marker for AF and ischemic stroke, and our ECG-AI measures remained significantly associated with outcomes even after adjusting for this sensitive biomarker of cardiac stretch that is widely performed clinically. This relationship suggests that our ECG-AI approach provides additional prognostic information beyond that captured by this established biomarker, and that combining these complementary biomarkers may improve the identification of individuals at elevated cardiovascular risk. Sex-stratified analyses revealed consistent associations of ECG-AI measures with AF and heart failure across both females and males, but suggested potentially stronger associations with ischemic stroke in females in the older CHS cohort, warranting further investigation in larger stroke samples to explore potential sex-specific mechanisms of atrial cardiopathy-related cerebrovascular events.

Our ECG-AI models predicted 5-year risks of AF and cardioembolic stroke as well as the most widely used clinical risk prediction tool, and the combination of clinical risk factors with ECG-AI enhanced prediction further. In the exploratory analysis of AF detection with 14-day cardiac monitoring, ECG-AI identified high-risk individuals with greater accuracy than both clinical risk factors and the cardiovascular biomarker NT-proBNP, suggesting that this approach should be evaluated as a potential targeted screening strategy for AF in a more definitive study. Of note, performing 14-day cardiac monitoring shortly after screening with a 12-lead ECG is likely to result in far superior performance compared with our analyses in MESA, where cardiac monitoring followed the 12-lead ECG by an average of 6 years. While cardiac imaging remains the gold standard for assessing left atrial function, its widespread application is limited by high costs, availability, and the need for expert interpretation. Our findings demonstrate that AI-enhanced ECG analysis can extract clinically meaningful information from widely available and inexpensive 12-lead ECGs, yielding consistently similar associations to clinical outcomes as the imaging-derived measurements.

This study has several limitations. The correlations between ECG-AI and CMR-derived measures were only moderate (*r* = 0.40–0.50), reflecting the differences in electrical versus anatomical and functional assessments, despite their robust associations with clinical outcomes. Training labels from UKB CMR were generated by a previously validated automated segmentation pipeline rather than by de novo manual annotation within this study, and therefore it is possible that residual label noise may attenuate the observed ECG-AI/CMR correlations. Although we did not directly validate ECG-AI measures again physician-derived measures in another subset of the UKB population, a high degree of segmentation accuracy is supported by published technical metrics (mean Dice 0.93–0.95), our random-sample audit (96% accuracy), and modest correlations of ECG-AI predictions with physician-traced LA measures in MESA (*r* = 0.30–0.34). The UKB population comprises predominantly European ancestry individuals who are healthier than the general population. Our GWAS analyses were further restricted to European ancestry participants to minimize population stratification, limiting the generalizability of our genetic findings to other ancestries. While the external validation of our ECG-AI in a large multi-ethnic cohort study demonstrates the transportability of these findings and models across diverse and heterogeneous populations^33,34^, the genetic architecture of ECG-AI traits in non-European ancestries remains to be characterized. Additionally, formal evaluation of model fairness (e.g., performance metrics stratified by race/ethnicity) was not performed and represents a limitation.

In MESA and CHS, some measures showed violations of the proportional hazards assumption over extended follow-up; however, sensitivity analyses restricted to 5 years showed substantial resolution of violations and stronger associations, indicating our primary results provide conservative estimates for clinically relevant time horizons. Our 14-day cardiac monitoring analysis was exploratory and subject to potential selection bias, as monitoring was performed only in surviving participants who returned for MESA Exam 6 (approximately 6 years after the index ECG) and met eligibility criteria; these findings require confirmation in a prospective study with monitoring performed shortly after ECG-based risk stratification. While ablation validated that Grad-CAM identifies functionally important ECG regions, saliency methods cannot confirm whether features are used in physiologically meaningful ways or capture all relevant patterns. While our minimal preprocessing approach (median beats and voltage rescaling) demonstrated transportability across the ECG acquisition systems in our study, whether additional harmonization would be needed for substantially different acquisition protocols remains to be determined. Learned representation analysis revealed CHS-specific embedding patterns that exceeded age-related variation, likely reflecting era-specific differences in acquisition protocols, selection bias, and secular trends; without labeled LA measurements in CHS, prediction accuracy in this cohort could not be validated. Although we demonstrated external validation in 2 independent research cohorts, broader validation across diverse clinical settings and ECG acquisition protocols is needed to establish widespread clinical utility.

Our ECG-AI approach represents a promising, accessible tool for assessing left atrial function and stratifying cardiovascular risk using standard 12-lead ECGs. In contrast with more resource- and technology-intensive methods, such as cardiac imaging, this approach could democratize access to advanced cardiovascular risk assessment across diverse healthcare settings. The public availability of our model facilitates further validation and implementation studies, potentially accelerating translation into clinical practice.

## Methods

### UK Biobank

The UK Biobank (UKB) is a cohort study that recruited 500,000 participants aged 40–69 years from the United Kingdom between 2006 and 2010^35^. This research has been conducted using the UK Biobank Resource under Application Number #40713. The baseline assessment included comprehensive questionnaires, blood and urine collection for biochemical measurements, anthropometry, and interviews. All UKB participants underwent whole-genome genotyping through the UK Biobank Whole-Genome Sequencing Consortium^36^.

All participants provided electronic informed consent at the UK Biobank assessment centers. The UKB received ethics approval from the North West Multi-Centre Research Ethics Committee.

Longitudinal follow-up for health outcomes of AF, stroke, and heart failure was performed through linkages with national databases on hospitalizations, primary care records, and death registries. Incident AF events were identified from self-reports, nurse interviews, hospital admission codes, death register cause-of-death codes, and UK Biobank first occurrence data, while ischemic stroke and heart failure were identified solely from diagnosis codes and cause-of-death codes (Supplementary Table 11). Participants diagnosed with incident AF before the imaging visit or those with pacemakers were excluded from all analyses. Additionally, participants whose imaging visits occurred after the UKB data censoring dates were excluded. Follow-up for this study extended through 2022. Hospital data were available until October 2022 for England, August 2022 for Scotland, and May 2022 for Wales. Death record data were censored in November 2022 across all sites.

### Multi-Ethnic Study of Atherosclerosis

The MESA is a community-based observational study of 6814 men and women, free of known CVD at baseline, representing 4 racial/ethnic groups (white, African American, Hispanic, and Chinese-American), aged 45–84 years and free of clinical CVD at enrollment^37^. Study participants were recruited in 2000–2 at 6 field centers in the US (Baltimore, MD; Chicago, IL; Forsyth County, NC; Los Angeles, CA; New York, NY; and St. Paul, MN). At telephone contacts every 9–12 months during follow-up, participants were asked to identify new hospitalizations and diagnoses, and medical records were obtained. Institutional review boards of all field centers approved the study protocol, and all participants gave written informed consent.

Clinically recognized AF was identified by an International Classification of Disease (ICD) hospital discharge diagnosis code (version 9: 427.31 or 427.32; version 10: I48) in any position; and for those enrolled in fee-for-service Medicare, by an inpatient, outpatient, or physician claim with an AF code^38^.

A panel reviewed medical records and adjudicated heart failure events according to standardized criteria. The present study included probable or definite heart failure events. Probable heart failure required heart failure symptoms, a physician diagnosis of heart failure, and treatment. Definite heart failure required at least one objective feature of heart failure, including abnormalities on chest X-ray, echocardiography, or ventriculography.

Ischemic stroke in MESA was adjudicated using information collected on all new cardiovascular conditions, hospital admissions, cardiovascular outpatient diagnoses, treatments, and deaths were obtained^39^. Two vascular neurologists from the MESA study events committee independently reviewed all medical records for endpoint classification and assignment of incidence dates. They reviewed and classified stroke as present if there was a focal neurologic deficit lasting 24 h or until death or, if <24 h, there was a clinically relevant lesion on brain imaging and there was no nonvascular cause. Patients with focal neurological deficits secondary to brain trauma, tumor, infection, or other nonvascular cause were excluded. Ischemic strokes were distinguished from hemorrhagic stroke using findings on imaging, surgery, autopsy, or some combination of these. Ischemic stroke subtypes were assigned based on an extension of the Trial of Org 10172 in Acute Stroke Treatment (TOAST) criteria to try to reduce the number classified as undetermined.

This study was exempt from institutional review board (IRB) approval as only de-identified data was used, but the parent MESA study was approved by the institutional review board of University of Washington and all participating sites (Columbia University, Johns Hopkins University, Northwestern University, University of California, Los Angeles (UCLA), University of Minnesota, Wake Forest University), and all study participants provided informed consent.

### Cardiovascular Health Study

The CHS is a cohort study of risk factors for cardiovascular diseases in adults aged 65 years and older, recruited from 4 field centers in the United States (Sacramento, CA; Hagerstown, MD; Winston-Salem, NC; Pittsburgh, PA)^40^. Between June 1989 and June 1990, 5201 participants were recruited from random samples of people from Medicare eligibility lists, and an additional 687 predominantly Black participants were recruited between November 1992 and June 1993. Study participants were seen in the clinic annually between enrollment and 1998-99 and were contacted by telephone at 6-month intervals through June 2015 to collect information to ascertain changes in health status, hospitalizations, and medication use^41^. Institutional review boards of all participating sites approved the study, and all study participants provided informed consent.

AF events were identified using a combination of electrocardiographic (ECG) findings from clinic visits through 1999, hospital discharge records indicating AF, and Medicare inpatient, outpatient, and physician (carrier) claims data. Resting 12-lead ECGs were performed at baseline and follow-up visits, with centralized readings to classify AF according to standardized criteria. For AF identified through Medicare data, diagnosis required either ECG evidence, 1 inpatient claim, or 2 outpatient or physician claims within 365 days using ICD-9-CM codes 427.31 or 427.32 in any diagnostic position.

Heart failure events in CHS were identified through in-person visits or telephone interviews and adjudicated by the central events committee, who reviewed pertinent data from the hospitalization or outpatient visit including history, physical examination, chest radiography, and medication use^42^. Self-reported physician diagnosis was confirmed by medical record documentation of characteristic symptoms (shortness of breath, fatigue, orthopnea, paroxysmal nocturnal dyspnea) and signs (edema, pulmonary rales, gallop rhythm, displaced left ventricular apical impulse), supporting radiographic findings, or current prescription of a diuretic with digitalis or a vasodilator in a participant with a prior heart failure diagnosis.

Ischemic stroke in CHS was adjudicated by an expert events committee, including neurologists from the study sites and CHS coordinating center, along with a neuroradiologist from the imaging reading center^43^. Ischemic stroke was characterized by the sudden onset of a focal neurological deficit persisting for at least 24 h, supported by compatible imaging findings on computed tomography scan or cardiac magnetic resonance imaging (CMR), and without evidence of intracranial hemorrhage or a nonvascular underlying cause. The adjudication committee categorized ischemic strokes into 4 subtypes based on the presumed infarction mechanism: cardioembolic, small vessel, large-artery atherosclerotic, or unknown/other causes.

This study was exempt from institutional review board (IRB) approval as only de-identified data were used, but the parent CHS study was approved by the institutional review boards of all participating sites (University of Washington, University of Vermont, University of Pittsburgh, Johns Hopkins University, Wake Forest University), and all study participants provided informed consent.

### Cardiac magnetic resonance imaging parameters

The UK Biobank Imaging Study began in 2014 and included magnetic resonance imaging of the brain, abdomen, and heart^44^. The cardiac magnetic resonance imaging (CMR) protocol consists of a 20-min scan performed using a 1.5 T scanner (MAGNETOM Aera, Syngo Platform VD13A, Siemens Healthcare, Erlangen, Germany)^45^. Cardiac volume and function parameters were obtained through 3 long-axis cines and complex short-axis stacks of balanced, steady-state free precession cines^45^. The first 5065 CMR scans were manually analyzed, and LA contours at end-systole and end-diastole were traced^46^. Using these manually annotated images, Bai et al.^17^ developed a fully automated convolutional network pipeline for the large-scale derivation of measures of cardiac structure and function. The accuracy of these automated methods was evaluated quantitatively by various technical metrics (Dice metric, contour distance metrics, and Hausdorff distance) and qualitatively by clinical experts^17^. For example, when the Dice metric was evaluated on 600 subjects, the mean Dice metric was 0.93 for the LA on the 2-chamber view, 0.95 for the LA on the 4-chamber view, demonstrating good accuracy. This algorithm is publicly available to the research community through GitHub.

We applied this fully convolutional network to all CMR scans from the first imaging visit available as of October 8th, 2023, to obtain left atrial minimal volume and left atrial maximal volume. Images from the first visit CMR studies were acquired from April 2014 to May 2023. The imaging substudy included participants who returned for a second assessment visit and consented to CMR imaging, which may represent a healthier subset of the overall UK Biobank population. Left atrial volumes were indexed to body surface area to obtain left atrial indexed minimal volume (LAVImin) and left atrial indexed maximal volume (LAVImax). LAEF was calculated through the following formula: [(maximal volume – minimal volume)/maximal volume × 100)]. A cardiologist then performed additional systematic quality control focused on the outliers to improve the reliability of the automated measurements. The 10 highest and 10 lowest measurements for each trait were visually inspected to identify segmentation errors. This process was then repeated in 1-mL increments for LA volumes and 1-percentile increments for LAEF. After a multidisciplinary discussion weighing the risks and benefits of over- or under-exclusion, we defined the quality control exclusion threshold as any increment in which ≥50% of the visually inspected images contained segmentation or imaging-plane errors. Based on this protocol, we included the following measures in our analysis: LA minimal volume > 8 mL, LA maximal volume > 24 mL, and LAEF ≤ 86%. To validate our process, we randomly sampled and visually inspected 50 measurements for each directly measured trait and observed a 96% accuracy rate.

In MESA, CMR imaging was performed at Exam 1 (2000–04, *n* = 5004) and Exam 5 (2011–13, *n* = 3016). Long-axis cine images were acquired during a 30–45-min protocol using 1.5-T scanners with ECG-gated fast gradient echo pulse sequence at baseline and steady-state free precession at Exam 5^47,48^. Ventricular measures were obtained by manually tracing the endocardial and epicardial borders of short-axis cine images at end-systole and end-diastole^49^. LA strain and phasic volume measures were obtained separately from baseline 2- and 4-chamber cine images. LA endocardial and epicardial borders were manually defined at end-systole by an experienced operator using Multimodality tissue tracking software (MTT, Toshiba, Tokyo, Japan). LA maximum, pre-atrial, and minimum contraction volumes were extracted from volume curves generated using area-length methods. The MTT software generated longitudinal strain curves for each atrial wall segment, and global longitudinal strain was calculated by averaging all segmental values at each time frame. The peak (reservoir) longitudinal LA strain value was measured from the global longitudinal strain curve^50^.

### Electrocardiograms

In UKB, ECG waveforms were extracted for all 62,926 subjects with resting digital ECGs measured at the Instance 2 visit that were available for download as of October 8th, 2023. ECGs were acquired using CASE® CardioSoft® Version 6 (GE Healthcare). The CardioSoft calculated median beat waveform from ECGs sampled at 500 Hz in each lead were extracted as Extensible Markup Language files. We included 8 non-derived leads from the 12-lead ECG: leads I, II, and V1 to V6. The remaining leads are mathematical transformations and do not include additional information. Quality-related exclusions included: poor-quality data (*N* = 1483 [2.4%]), no interpretation possible (*N* = 3 [<0.1%]), suspect arm/limb lead reversal (*N* = 429 [0.7%]), undetermined rhythm (*N* = 265 [0.4%]), and implausible ventricular rates <20 or >140 bpm (*N* = 45 [0.1%]). Clinical exclusions included: AF (*N* = 1153 [1.8%]), atrial flutter (*N* = 98 [0.2%]), and pacemaker rhythm (*N* = 174 [0.3%]). Due to potential overlap between categories (e.g., AF with poor quality), the total number of excluded ECGs was *N* = 3230 (5.1%) as detailed in Supplementary Fig. 1. Participants excluded for quality-related reasons had similar baseline characteristics similar to the included population (mean age 65.1 ± 7.7 vs. 63.8 ± 8.2 years), while those excluded for AF/flutter or pacemaker presence were older (71.4 ± 6.5 years), consistent with prevalent disease.

In CHS and MESA, resting 10-s 12-lead ECGs sampled at 500 Hz were recorded in a uniform fashion in all participants at baseline using MAC PC ECG Machines (Marquette Electronics Inc, Milwaukee, WI). All ECGs were visually inspected for technical errors and inadequate quality centrally at the Epidemiological Cardiology Research Center (EPICARE) of Wake Forest University School of Medicine (Winston-Salem, NC) using General Electric (GE) 12-SL software (GE, Milwaukee, WI) running under GE MUSE and Magellan Research Work Station. Median beat waveforms were extracted from the GE MUSE report.

The UKB digital ECGs were reported with 5 μV per least significant bit (μV/LSB). Median ECGs in CHS were reported with 1 μV/LSB, and in MESA, with 4.88. They were both rescaled to 5 μV/LSB prior to prediction from the ECG-AI model. Beyond voltage rescaling and extraction of median beats, no additional preprocessing, filtering, baseline correction, or harmonization was performed. This minimal preprocessing pipeline was applied identically to all cohorts without cohort-specific adjustments or model retraining.

In CHS and MESA, the PTFV1 is defined as the duration (milliseconds) of the downward deflection (terminal portion) of the P-wave in lead V1 multiplied by the absolute value of its amplitude (μV).

### Echocardiography

Left atrial (LA) strain was measured using archived echocardiograms originally acquired in 1989–1990 and 1994–1995^51^, which were digitized from Super VHS tapes between 2016 and 2018. LA reservoir strain was assessed from apical 4-chamber views using speckle-tracking echocardiography (TOMTEC v4.5), with the LA endocardial border manually traced and strain curves generated from well-tracked segments^52^. Analyses were performed by experienced readers with ECG gating, and all strain values were reported as positive absolute percentages relative to the ventricular cycle as the reference point. LA reservoir strain was defined as the peak average LA strain and was calculated as the difference between peak strain values and the reference point.

### ECG-AI model

ECG-AI models to predict CMR-measured LAVmin, LAVmax, and LAEF were trained in UKB using a deep convolutional neural network (CNN). We used the median beat waveform as model input because it reduces dimensionality while preserving morphological features relevant to structural cardiac prediction. The median beat approach eliminates beat-to-beat variability and ectopic beats that do not reflect underlying cardiac structure and improves robustness to acquisition noise and device-specific artifacts through signal averaging.

The UKB sample was randomly divided into 45% training, 5% validation, and a 50% hold-out test set, with each individual contributing data to only 1 split; this resulted in balanced baseline characteristics across samples (Supplementary Table 1). The large allocation to the test sample was done to provide adequate power for extensive epidemiologic analyses of association with clinical outcomes.

The CNN core architecture is an inception network that comprises 4 inception modules followed by 3 fully connected layers. An initial 1D convolutional layer is applied to extract low-level features from the raw ECG data, followed by batch normalization to stabilize the activations. Next, the model employs a series of 4 inception-style blocks. In each block, the network processes the features in 3 parallel branches using convolutional layers with different kernel sizes scaled by factors of 1.0, 1.5, and 2.0 relative to the base filter length; each branch is followed by batch normalization. The outputs from these branches are then concatenated along the feature axis to integrate information captured at multiple temporal scales. Next, a global average pooling layer reduces the concatenated feature maps to a single vector, which is then fed through a sequence of fully connected (dense) layers.

For model training, we performed a random hyperparameter search with 20 configurations per phenotype (LAVmin, LAVmax, and LAEF). The hyperparameter search space included: filter counts [40, 80, and 120], kernel sizes [6, 12, 24, 48, and 96] samples, dense layer sizes of [52, 256] units for layer 1, [4, 8] units for layer 2, and 4 units for layer 3, batch sizes [12, 24], and learning rates sampled from a log-uniform distribution between 10^−5^ and 10^−^^2^. All models used the Adam optimizer^53^ with “same” padding, were trained for up to 100 epochs, and employed early stopping^54^ with patience set to 3 epochs. Model selection was based on validation set mean squared error. Twenty models were trained, and the top 5 models for each outcome were ensembled using the mean of the predictions. The specific hyperparameters for each model in the final ensembles are provided in Supplementary Table 3.

### Statistical analysis

Left atrial measures were evaluated as continuous variables to predict event outcomes. Each measure was centered and scaled to have a mean of 0 and a standard deviation of 1. Associations with outcomes were assessed using Cox proportional hazards models. Event counts are reported for all analyses and determine the effective degrees of freedom for survival models. All statistical hypothesis tests were two-sided with a significance level of *α* = 0.05 unless otherwise specified. All *P* values are reported without adjustment for multiple testing. Our primary scientific interest is comparing effect sizes and consistency of associations across cohorts rather than binary hypothesis testing, with robustness demonstrated by consistent directions and magnitudes of associations across 3 independent cohorts.

AF analyses were adjusted for age, sex, race, height, body mass index (BMI), systolic blood pressure (SBP), diastolic blood pressure (DBP), current smoking, antihypertensive medication use, prevalent diabetes mellitus (DM), heart failure, and myocardial infarction (MI). Individuals with prior AF or a pacemaker were excluded.

Ischemic stroke analyses were adjusted for age, sex, race, BMI, SBP, current smoking, antihypertensive medication use, DM, prevalent heart failure, and MI. Individuals with prior AF, ischemic stroke, or a pacemaker were excluded.

Heart failure analyses were adjusted for age, sex, race, BMI, SBP, current smoking, antihypertensive medication use, DM, total cholesterol, high-density lipoprotein cholesterol, and left ventricular ejection fraction (LVEF). LVEF was modeled as a continuous value in UKB and MESA and categorized as qualitatively normal or abnormal in CHS. Individuals with prior AF, heart failure, or a pacemaker were excluded.

Race and ethnicity were self-reported at baseline and included as covariates to account for social and environmental factors. In MESA, race/ethnicity categories were White, African American, Hispanic, or Chinese American; in UKB and CHS, race was categorized as White or other. Race was not used as a proxy for biological or genetic differences. All covariates were assessed at the time of CMR, echocardiogram, or ECG measurement. Sex was determined by self-report at baseline assessment and included as a covariate in all multivariable models. All analyses included both sexes.

Proportional hazards assumptions were tested using Schoenfeld residuals for all exposure variables. In MESA and CHS, where some measures showed evidence of non-proportionality over extended follow-up, sensitivity analyses restricted to 5-year follow-up were performed (Supplementary Table 5). A complete case analysis was performed due to low rates of missing data for our primary covariates (0.2% in MESA and 2.6% in CHS). The LVEF covariate in the heart failure models had higher missingness (26% in MESA and 12% in CHS), reflecting that these measures were obtained in selected subsamples of the cohorts rather than all participants. To assess the potential impact of this missingness, a sensitivity analysis using multiple imputation by chained equations (MICE, version 3.19) was performed; results were consistent with the complete case analysis (Supplementary Table 12).

Sensitivity analyses for associations of LA measures and AF, ischemic stroke, and heart failure were performed as above, adding natural log-transformed N-terminal pro-B-type natriuretic peptide (NT-proBNP) as a covariate. NT-proBNP assessments were performed in selected subsamples rather than the full cohort and had moderate missingness (18% in MESA and 23% in CHS); MICE was therefore used for these analyses.

### Software

All association, survival, and risk-prediction analyses were performed in R version 4.3.1, using the survival (v3.5.8) package for Cox proportional hazards models and Schoenfeld-residual tests, mice (v3.19.0) for multiple imputation by chained equations, and pROC (v1.18.5), precrec (v0.14.4), survAUC (v1.3.0), and risksetROC (v1.0.4.1) for discrimination metrics and bootstrap confidence intervals; data handling and prediction used dplyr (v1.1.4), data.table (v1.15.4), tidyr (v1.3.1), haven (v2.5.4), caret (v6.0.94) and dcurves (v0.5.1). The ECG-AI deep-learning models were trained and applied in Python version 3.6.9 using TensorFlow 2.3.0 and Keras 2.4.3, with scikit-learn 0.23.2, NumPy 1.18.5, pandas 1.1.2 and SciPy 1.4.1. ECG median-beat preprocessing (neurokit2 0.2.12), Grad-CAM, ablation analyses, and learned-representation analyses (t-SNE/UMAP) were performed in Python version 3.11.14 using TensorFlow 2.20.0, scikit-learn 1.8.0, SciPy 1.16.3, NumPy 2.3.5, pandas 2.3.3, matplotlib 3.10.8 and umap-learn 0.5.11. Genome-wide association analyses used a linear mixed model implemented in the GENESIS package (v2.16.1); LD Score regression used LDSC v1.0.1; and variants were annotated against GENCODE v26.

### Risk score comparison

To evaluate the ability of ECG-AI to predict 5-year risk for AF and the AF related outcomes of ischemic and cardioembolic stroke assessed risk scores, we evaluated 4 models: (1) age and sex only (base), (2) base plus all 3 ECG-AI left atrial measures (ECG-AI), (3) CHARGE-AF risk score alone (CHARGE-AF), and (4) CHARGE-AF + ECG-AI (combined). Each model was trained in the test partition of UKB to predict 5-year AF using logistic regression models. Participants in the testing set of UKB were limited to those > = 65 to align with the external populations. The weights for each variable were then used to create scores in the external cohort studies. The models were evaluated in the joint MESA-CHS sample to predict incident events within 5 years. Ninety-five percent confidence intervals were created using 10,000 bootstrap samples. CHARGE-AF was estimated on a group of studies that included CHS, and therefore the CHARGE-AF estimates may perform better in this analysis than in a fully independent sample.

### AF detection with cardiac monitoring

We used the ECG-AI model to predict LA measurements at MESA Exam 5 (2010–12). The highest quintile for LAVImin and LAVImax, and the lowest quintile for LAEF were used to define a “high-risk” stratum for each measurement. The CHARGE-AF score was calculated on participants at Exam 5, and the highest quintile of scores was used to define the “high-risk” strata, and the top quintile of NT-proBNP defined the “high-risk” population for NT-proBNP. In MESA at Exam 6 (2016–18), 1528 participants were screened using a ZioPatch monitor that detects and stores up to 14 days of cardiac rhythm (Zio Patch XT, iRhythm Technologies, Inc, San Francisco, CA)^16^. Monitor-detected AF included both AF and atrial flutter; AF was defined as an irregularly irregular rhythm with absent P waves lasting at least 30 s. Participants were excluded if AF was detected by ECG at Exam 5, if they had <24 h of cardiac monitoring, a paced rhythm during monitoring, they were missing data for the CHARGE-AF score or NT-proBNP measurements. Area under the curve for receiver-operator curves were reported along with sensitivity and specificity.

### Gradient-weighted class activation mapping

Gradient-weighted class activation mapping (Grad-CAM) was used to identify the regions of the ECG most influential in making atrial measure predictions. The last convolutional layer of the model with the lowest validation loss was used to explore the model gradients. The average gradients were calculated and visualized as a heatmap for the ECGs with the 50 highest volume predictions, or 50 smallest predictions for LAEF. For volumes, the maximum gradient multiplied by the convolutional outputs were plotted; for LAEF, the minimal gradient multiplied by the convolutional outputs were used. The average amplitudes for lead II of the 50 visualized ECGs were plotted for context. In addition, the amplitude for lead II of the 50 ECGs predicting the smallest atrial volumes, or the 50 largest ejection fractions, were compared to the Grad-CAM intensity locations.

To assess the functional importance of the regions identified by Grad-CAM, an ablation analysis was performed. Two ablation conditions were compared: (1) saliency-guided ablation, in which the top 5%, 10%, 20%, or 30% of regions ranked by saliency were zeroed out, and (2) random ablation, in which the same fraction of randomly selected regions were zeroed out. Random ablation was repeated 5 times at each ablation level, and results were averaged. The ablated signals were then passed through the model to generate predictions, and performance was compared with the baseline (no ablation) using Pearson correlation, mean squared error (MSE), and mean absolute error (MAE). Performance degradation from saliency-guided ablation was compared with that from random ablation at each ablation level to determine whether regions highlighted by Grad-CAM were more critical for model predictions than randomly selected regions.

### Learned representation analysis

Learned representations were extracted from the penultimate dense layer of ECG deep learning models. Embeddings from the top 5 ensembled models per outcome were concatenated along the feature dimension. Two-dimensional t-SNE embeddings were computed using perplexity = 200, random_state = 42, and max_iter = 1000. Side-by-side visualizations showed cohort membership and age bins (<65, 65–75, and 75+ years).

To quantify whether CHS differed from UKB/MESA beyond age alone, we performed linear regression of the first t-SNE component on age and CHS indicator (CHS vs. non-CHS). An F-test compared the age-only model to the age + CHS model to assess whether adding cohort membership significantly improved fit.

For age-matched analysis, participants aged 70–75 years were selected from each cohort. Centroids were computed for UKB, MESA, and the combined UKB/MESA group. The Euclidean distance from each CHS participant to the UKB/MESA centroid was calculated and compared to the distance between UKB age groups 10 years apart (60–70 vs. 70–80 years).

### Genome-wide association studies

In the UKB, 488,377 participants were genotyped for over 800,000 variants using DNA extracted from blood samples collected at the baseline examination^55^. Genotypes were directly called using the UK Biobank Axiom (Affymetrix, Santa Clara, California) and the UK Applied Biosystems Array. Imputation was carried out using the Haplotype Reference Consortium and UK10K + 1000 Genomes reference panels. Variants that did not pass quality control checks were excluded from further UKB analyses. We performed genome-wide association studies (GWAS) of the 5 ECG-AI left atrial traits: LAVmin, LAVmax, LAVImax, LAVImin and LAEF. Individuals with a prior history of AF, a pacemaker, or non-European ancestry were excluded. Analyses were performed using a linear mixed model to account for relatedness using the GENESIS package^56^. To improve statistical power and control for Type I errors with a non-normal phenotype distribution, we implemented a fully adjusted two-stage procedure for rank-normalization when fitting the null model^57^. The residuals from a regression model adjusting for age at MRI, sex, ten principal components of ancestry, and an indicator for UK BiLEVE or UK Biobank Axiom array were rank-normalized. Transformed residuals were then fit in a null model adjusting for the same fixed effects and a sparse genetic relationship matrix (GRM). The GRM was constructed from the KING kinship coefficients limited to third-degree relatives from UKB. The resulting null model was used to perform genome-wide score tests of genetic association for all individual variants with minor allele frequency (MAF) > 0.005. Genome-wide significance was defined as *P* < 5 × 10^−^^8^. We used genomic control factor lambda and the regression intercept from LD Score regression (LDSC) v1.0.1^58,59^ to assess inflation due to population stratification or polygenicity. We estimated the trait heritability (*h*^*2*^) using the LDSC regression slope. For each GWAS, index variants were identified for each locus by iteratively taking the variant with the lowest *p*-value and excluding other variants within a 500 kb window. Nearest genes were annotated using GENCODE v26^60^. We further annotated these index SNPs by identifying any AF index SNP from the 142 index SNPs from Neilson et al.^24^ within 500 kb.

### Genetic trait correlations

We used LDSC to estimate genetic correlations between 11 clinically related outcomes and risk factors and (1) the CMR atrial traits and (2) the ECG-AI predicted traits using default settings, which leave the intercept unconstrained. Trait GWAS were restricted to European ancestry individuals and limited to variants with MAF > 0.01.

Outcome trait GWAS used the largest available European ancestry meta-analyses for AF^24^, ischemic and cardioembolic stroke^61^, heart failure^62^, Alzheimer’s disease^63^, and type II diabetes^64^. Correlation for related traits and risk factors used large publicly available GWAS of body mass index^65^, SBP and DBP^66^, PR interval^67^, and QT interval^68^. To evaluate LVM and LVEF, we performed GWAS of these traits using the measures derived from the Bai et al. segmentation method^17^. LVM was indexed to body surface area. Analyses were restricted to individuals of European ancestry and variants with a minor allele frequency >0.005. GWAS analyses used the methods described above, adjusting for age at imaging visit, sex, 10 principal components of ancestry, UK BiLEVE or UK Biobank Axiom array, and imaging center.

### Reporting summary

Further information on research design is available in the Nature Portfolio Reporting Summary linked to this article.

## Supplementary information


Supplementary Information
Description of Additional Supplementary File
Supplementary Data 1
Supplementary Data 2
Supplementary Data 3
Supplementary Data 4
Supplementary Data 5
Reporting Summary
Transparent Peer Review File


## Data Availability

The data supporting the findings from this study are available within the manuscript and its Supplementary Information. UK Biobank data are made available to researchers from research institutions as described at this site: https://www.ukbiobank.ac.uk/enable-your-research. Data for the Multi-Ethnic Study of Atherosclerosis and Cardiovascular Health Study can be requested at https://internal.mesa-nhlbi.org/ and https://chs-nhlbi.org/node/6222, respectively. GWAS summary statistics are publicly available under accession numbers GCST006414 (atrial fibrillation), GCST90104540 (ischemic stroke), GCST90104541 (cardioembolic stroke), GCST90104542 (large artery stroke), GCST90104543 (small vessel stroke), GCST90132314 (coronary artery disease), GCST90239658 (low density lipoprotein cholesterol), GCST90239652 (high density lipoprotein cholesterol), GCST90239664 (triglycerides), GCST90310294 (systolic blood pressure), GCST90310295 (diastolic blood pressure), GCST009004 (BMI) and GCST010320 (PR interval) at https://www.ebi.ac.uk/gwas/home. The GWAS summary statistics for heart failure [https://cvd.hugeamp.org/dinspector.html?dataset=Shah2020_HF_EU], type 2 diabetes [https://www.diagram-consortium.org/downloads.html], and QT interval [https://www.arkinglab.org/resources/] are available at their respective repositories.

## Code availability

The fully trained model and weights are available for download at https://github.com/j-brody/ecg_ai_atrialmeasures^69^.
