# Supplementary material for: Deep learning prediction of left atrial structure and function from 12-lead electrocardiograms
Source: Nat Commun. 2026 Jul 31;17:9273. doi: 10.1038/s41467-026-76155-6 (PMC13527101; doi:10.1038/s41467-026-76155-6)
Supplement: Supplementary file 1 [file 41467_2026_76155_MOESM1_ESM.pdf]

Supplementary Table 1: Participant characteristics for UKB training and testing partitions

	**UKB training partition**	**UKB test partition**
Age, mean (SD), years	65.2 (7.7)	65.0 (7.7)
Male sex, N (%)	11608 (48.0)	11205 (47.5)
Race, white, N (%)	23310 (96.5)	22722 (96.4)
Height, mean (SD), cm	169.1 (9.2)	169.0 (9.2)
Weight, mean (SD), kg	76.2 (15.2)	76.0 (15.1)
Body mass index, mean (SD) kg/m2	26.6 (4.4)	26.5 (4.4)
SBP, mean (SD), mmHg	140.4 (19.1)	140.5 (19.2)
DBP, mean (SD), mmHg	79.0 (10.1)	78.9 (10.1)
Antihypertensive medication use, N (%)	5862 (24.3)	5558 (23.6)
Current smoking, N (%)	827 (3.4)	775 (3.3)
Diabetes mellitus, N (%)	1316 (5.4)	1292 (5.5)
Prevalent heart failure, N (%)	120 (0.5)	86 (0.4)
Prevalent myocardial infarction, N (%)	552 (2.3)	503 (2.1)
Total cholesterol, N (%), mg/dL	221.5 (42)	221.6 (41.8)
HDL cholesterol, N (%), mg/dL	57.4 (14.7)	57.5 (14.7)
LV ejection fraction category, Normal, N (%)	22951 (95.0)	22375 (94.9)
RR interval, mean (SD), ms	1001.6 (161.0)	1002.7 (160.2)
QT interval, mean (SD), ms	420.4 (30.8)	420.3 (30.8)
PR interval, mean (SD), ms	165.5 (27.5)	165.6 (27.5)
LA minimal volume MRI, mean (SD), mL	29.3 (13.1)	29.0 (12.8)
LA maximal volume MRI, mean (SD), mL	72.9 (22.2)	72.5 (21.7)
LA EF, mean (SD), %	60.8 (8.0)	61.0 (7.9)

SD=standard deviation, SBP=systolic blood pressure, DBP=diastolic blood pressure, LV=left ventricular, HDL=high-density lipoprotein, LA-left atrial, EF=ejection fraction

Supplementary Table 2: Participant baseline characteristics

	UKB	CHS	MESA
Age, mean (SD), years	65.0 (7.7)	72.7 (5.5)	62 (10.2)
Male sex, N (%)	11205 (47.5)	2198 (41.5)	3162 (47.2)
Race, N (%)			
White	22722 (96.4)	4436 (83.8)	2553 (38.1)
Chinese	-	-	798 (11.9)
Black	-	-	1863 (27.8)
Hispanic/Latino	-	-	1482 (22.1)
Height, mean (SD), cm	169.0 (9.2)	164.7 (9.4)	166.4 (10)
Weight, mean (SD), kg	76.0 (15.1)	72.7 (14.7)	78.7 (17.4)
Body Mass Index, mean (SD) kg/m2	26.5 (4.4)	26.7 (4.7)	28.4 (5.5)
SBP, mean (SD), mmHg	140.5 (19.2)	136.8 (22.1)	126.5 (21.5)
DBP, mean (SD), mmHg	78.9 (10.1)	70.9 (11.6)	72.0 (10.3)
Antihypertensive medication use, N (%)	5558 (23.6)	2511 (47.4)	2467 (36.8)
Current smoking, N (%)	775 (3.3)	632 (11.9)	876 (13.1)
Diabetes mellitus, N (%)	1292 (5.5)	832 (15.7)	843 (12.6)
Prevalent heart failure, N (%)	86 (0.4)	205 (3.9)	0 (0)
Prevalent myocardial infarction, N (%)	503 (2.1)	489 (9.2)	0 (0)
Total cholesterol, N (%), mg/dL	221.6 (41.8)	211.9 (38.9)	194.2 (35.8)
HDL cholesterol, N (%), mg/dL	57.5 (14.7)	54.3 (15.8)	50.9 (14.8)
LV ejection fraction category, Normal, N (%)	22375 (94.9)	4259 (80.4)	4837 (72.2)
RR interval, mean (SD), ms	1002.7 (160.2)	949.3 (159.2)	972.3 (148.9)
QT interval, mean (SD), ms	420.3 (30.8)	416.5 (35.4)	411.7 (30.6)
PR interval, mean (SD), ms	165.6 (27.5)	169.8 (31.4)	165.9 (24.9)
PTFV1, mean (SD), µV*ms	-	2751.2 (2255.6)	2190.2 (1830.3)
LA average peak longitudinal strain, mean (SD), %	-	40.9 (15.3)	36.3 (10.9)
NT-proBNP, mean (SD), pg/mL	-	249.5 (754)	102 (251)
CMR LAVImin, mean (SD), mL	15.4 (6.4)	-	12.5 (6.7)
CMR LAVImax, mean (SD), mL	38.6 (10.6)	-	30.2 (9.9)
CMR LAEF, mean (SD), %	61.0 (7.9)	-	59.2 (3.5)
ECG-AI LAVImin, mean (SD), mL	15.9 (3.5)	18.8 (5.4)	17.2 (4.1)
ECG-AI LAVImax, mean (SD), mL	38.9 (5.5)	43.1 (7.6)	41.1 (6.5)
ECG-AI LAEF, mean (SD), %	60.7 (3.1)	58.4 (4.4)	61.0 (11.8)

SD=standard deviation, SBP=systolic blood pressure, DBP=diastolic blood pressure,LV=left ventricular, HDL=high-density lipoprotein, LA-left atrial, EF=ejection fraction

Supplementary Table 3: Model parameters for ECG-AI training

outcome	model index	filter number	filter length	batch size	learn rate	dense1 size	dense2 size	dense3 size	patience	mean squared error	mean absolute error	epochs
LAVmin	16	40	24	24	0.00042	256	4	4	3	127.37	8.58	13
LAVmin	10	80	24	24	0.00083	52	8	4	3	128.39	8.48	14
LAVmin	6	80	24	24	1.7E-05	256	8	4	3	129.57	8.55	26
LAVmin	18	80	24	24	0.00014	52	4	4	3	131.09	8.64	12
LAVmin	19	80	48	24	3.8E-05	52	8	4	3	131.26	8.64	9
LAVmax	4	40	48	24	0.00021	256	4	4	3	375.78	15.12	13
LAVmax	3	80	96	24	0.00063	256	4	4	3	383.35	15.22	10
LAVmax	18	80	24	12	1.8E-05	52	8	4	3	385.97	15.10	7
LAVmax	19	80	48	24	0.00525	52	8	4	3	385.99	15.13	13
LAVmax	5	40	48	24	5.9E-05	256	8	4	3	386.02	15.31	9
LAEF	7	60	24	24	0.00015	256	8	4	3	52.54	5.67	16
LAEF	16	60	48	12	3.8E-05	52	8	4	3	53.03	5.70	11
LAEF	13	40	48	12	0.00126	256	4	4	3	53.13	5.67	14
LAEF	2	60	96	24	0.00027	256	4	4	3	53.43	5.72	10
LAEF	6	60	48	24	0.00029	256	4	4	3	53.49	5.72	13

Supplementary Table 4: PR interval associations with outcomes

Study	Outcome	Event/N	HR (95% CI)	P value
UKB	Atrial fibrillation	331/18804	1.01 (0.91-1.12)	0.92
	Ischemic stroke	71/16347	1.15 (0.93-1.42)	0.19
	Heart failure	118/16352	1.08 (0.92-1.27)	0.32
MESA	Atrial fibrillation	1298/6677	1.02 (0.97-1.08)	0.42
	Ischemic stroke	253/5637	0.97 (0.85-1.10)	0.61
	Heart failure	244/4190	1.16 (1.03-1.30)	0.01
CHS	Atrial fibrillation	2232/5154	1.05 (1.00-1.10)	0.04
	Ischemic stroke	777/4940	0.99 (0.92-1.07)	0.87
	Heart failure	1250/4392	1.07 (1.01-1.14)	0.02

Supplementary Table 5: Pearson correlations with markers of cardiac function in MESA

	LVEF	PTFV1	log NT-proBNP
ECG-AI LAVImin	-0.019	0.160	0.372
ECG-AI LAVImax	0.036	0.124	0.325
ECG-AI LAEF	0.080	-0.139	-0.331
CMR LAVImin	-0.089	0.136	0.279
CMR LAVImax	0.028	0.091	0.266
CMR LAEF	0.183	-0.128	-0.162

Supplementary Table 6: ln NT-proBNP associations results

Study	Outcome	Event/N	HR (95% CI)	P value
MESA	Atrial fibrillation	1111/5485	1.72 (1.59-1.86)	6.32E-42
	Ischemic stroke	213/4734	1.16 (0.97-1.38)	0.1
	Heart failure	200/3536	2.04 (1.74-2.39)	1.79E-18
	Cardioembolic stroke	63/4734	1.97 (1.44-2.70)	2.33E-05
	Small vessel stroke	65/4734	0.81 (0.60-1.11)	0.19
	Large-vessel atherothrombotic stroke	31/4734	1.06 (0.68-1.68)	0.79
CHS	Atrial fibrillation	1713/3988	1.49 (1.40-1.58)	2.23E-39
	Ischemic stroke	597/3842	1.29 (1.17-1.42)	2.91E-07
	Heart failure	957/3368	1.69 (1.56-1.84)	9.55E-38
	Cardioembolic stroke	191/3842	1.96 (1.65-2.32)	8.33E-15
	Small vessel stroke	107/3842	1.05 (0.84-1.32)	0.68
	Large-vessel atherothrombotic stroke	38/3842	1.41 (0.97-2.05)	0.07

Supplementary Table 7: Association of ECG-AI predicted measures adjusting for ln NT-proBNP

Study		Event/N	HR (95% CI)	P value
MESA	Atrial fibrillation			
	ECG-AI LAVImin	1304/6691	1.21 (1.14-1.27)	2.73E-11
	ECG-AI LAVImax	1304/6691	1.18 (1.11-1.26)	6.38E-07
	ECG-AI LAEF	1304/6691	0.85 (0.81-0.90)	1.23E-09
	Ischemic stroke			
	ECG-AI LAVImin	256/5669	1.12 (0.99-1.26)	0.08
	ECG-AI LAVImax	256/5669	1.12 (0.97-1.28)	0.13
	ECG-AI LAEF	256/5669	0.90 (0.79-1.01)	0.08
	Heart failure			
	ECG-AI LAVImin	365/5671	1.18 (1.07-1.30)	1.24E-03
	ECG-AI LAVImax	365/5671	1.18 (1.06-1.32)	3.20E-03
	ECG-AI LAEF	365/5671	0.84 (0.76-0.92)	2.99E-04
CHS	Atrial fibrillation			
	2290/5294	2290/5294	1.28 (1.22-1.35)	3.25E-23
	2290/5294	2290/5294	1.14 (1.08-1.20)	3.45E-06
	2290/5294	2290/5294	0.77 (0.74-0.81)	2.12E-29
	Ischemic stroke			
	802/5086	802/5086	1.17 (1.08-1.27)	1.40E-04
	802/5086	802/5086	1.12 (1.03-1.22)	7.98E-03
	802/5086	802/5086	0.85 (0.79-0.91)	1.06E-05
	Heart failure			
	1275/4484	1275/4484	1.28 (1.20-1.36)	2.33E-14
	1275/4484	1275/4484	1.23 (1.15-1.31)	6.23E-09
	1275/4484	1275/4484	0.82 (0.78-0.88)	1.68E-09

Supplementary Table 8: AUCs for 5-year event prediction in MESA and CHS

Outcome		Exposure	AUROC		AUPRC	
Atrial fibrillation	AUCs	AgeSex	0.71 (0.69-0.73)		0.16 (0.15-0.18)	
		CHARGE-AF	0.74 (0.72-0.76)		0.18 (0.16-0.20)	
		ECG-AI	0.75 (0.74-0.77)		0.23 (0.20-0.25)	
		Combined	0.76 (0.75-0.78)		0.22 (0.20-0.25)	
		**Exposure**	**Δ AUROC**	**P**	**Δ AUPRC**	**P**
	Delta AUCs	ECG-AI - AgeSex	0.041 (0.030-0.053)	<1e-4	0.062 (0.043-0.083)	<1e-4
		CHARGE-AF - AgeSex	0.028 (0.018-0.039)	<1e-4	0.016 (0.004-0.028)	0.013
		Combined - AgeSex	0.054 (0.041-0.066)	<1e-4	0.061 (0.041-0.082)	<1e-4
		Combined - CHARGE-AF	0.024 (0.017-0.034)	<1e-4	0.046 (0.030-0.063)	<1e-4
		**Exposure**	**AUROC**		**AUPRC**	
Cardioembolic stroke	AUCs	AgeSex	0.76 (0.71-0.80)		0.016 (0.010-0.032)	
		CHARGE-AF	0.80 (0.75-0.85)		0.026 (0.016-0.046)	
		ECG-AI	0.82 (0.77-0.87)		0.033 (0.019-0.062)	
		Combined	0.83 (0.79-0.88)		0.042 (0.023-0.082)	
		**Exposure**	**Δ AUROC**	**P**	**Δ AUPRC**	**P**
	Delta AUCs	ECG-AI - AgeSex	0.065 (0.026-0.104)	0.001	0.016 (-0.001-0.044)	0.057
		CHARGE-AF - AgeSex	0.041 (0.008-0.075)	0.017	0.010 (0.000-0.023)	0.042
		Combined - AgeSex	0.078 (0.040-0.115)	<1e-4	0.026 (0.004-0.064)	0.016
		Combined - CHARGE-AF	0.037 (0.008-0.066)	0.012	0.016 (-0.002-0.051)	0.099

AUROC = Area under the Receiver Operating Characteristic curve; AUPRC = Area under the Precision–Recall curve

Supplementary Table 9: Prediction of subclinical AF in MESA by high-risk quintiles

Predictor	AUROC	95% CI	PPV	Sensitivity
LAVImin	0.65	0.59-0.70	0.14	0.48
LAVImax	0.59	0.54-0.65	0.11	0.38
LAEF	0.66	0.60-0.71	0.14	0.50
NT-proBNP	0.62	0.56-0.68	0.12	0.43
CHARGE-AF	0.58	0.53-0.63	0.10	0.35

Each contrast is comparing the 'high-risk' quintile for each predictor against the lowest 4 quintiles, which is the highest quintile for LAVImin, LAVImax, NT-proBNP and CHARGE-AF scores, and the lowest quintile for LAEF.

PPV=Positive predictive value, ROC-AUC receiver operator curve area under the curve

Supplementary Table 10: Linkage disequilibrium score regression (LDSC) heritability

Trait	Total Observed scale h2	Lambda GC	Ratio	Mean Chi^2	Intercept
LAVmin	0.1444 (0.0185)	1.0772	0.1308 (0.0669)	1.1027	1.0134 (0.0069)
LAVmax	0.1411 (0.0202)	1.0833	0.1582 (0.0686)	1.1039	1.0164 (0.0071)
LAVImin	0.1433 (0.0185)	1.0895	0.1089 (0.0683)	1.1008	1.0110 (0.0069)
LAVImax	0.1677 (0.0190)	1.1019	0.0894 (0.0593)	1.116	1.0104 (0.0069)
LAEF	0.1511 (0.0180)	1.0833	0.1157 (0.0657)	1.1057	1.0122 (0.0069)

Supplementary Table 11: Codes for defining outcomes in UKB

Outcome	UKB Codes
Atrial fibrillation	Self-report codes: 1471, 1483
	ICD-9 codes: 427.3
	ICD-10 code: I48
	First occurrence code: I48
Ischemic stroke	ICD-9 codes: 433, 434, 436
	ICD-10 codes: I63, I64
Heart failure	ICD-9 codes: 428
	ICD-10 codes: I50, I110, I1130
	First occurrence code: I50

Supplementary Table 12: Multiple imputation by chained equations (MICE) imputed results for heart failure

MESA

	Event/N	HR (95% CI)	P value
ECG-AI LAVImin	365/5671	1.36 (1.25-1.48)	1.36E-11
ECG-AI LAVImax	365/5671	1.41 (1.27-1.57)	8.53E-10
ECG-AI LAEF	365/5671	0.72 (0.66-0.79)	8.44E-12
LA Strain	365/5671	0.93 (0.80-1.08)	0.31
ECG PTFV1	365/5671	1.19 (1.08-1.31)	3.98E-04

CHS

	Event/N	HR (95% CI)	P value
ECG-AI LAVImin	1275/4484	1.44 (1.36-1.53)	9.53E-34
ECG-AI LAVImax	1275/4484	1.37 (1.29-1.46)	9.26E-23
ECG-AI LAEF	1275/4484	0.74 (0.70-0.78)	3.37E-26
LA Strain	1275/4484	0.80 (0.75-0.86)	9.59E-09
ECG PTFV1	1275/4484	1.11 (1.04-1.17)	6.74E-04

Supplementary Fig. 1: Consort diagram of UKB analyses

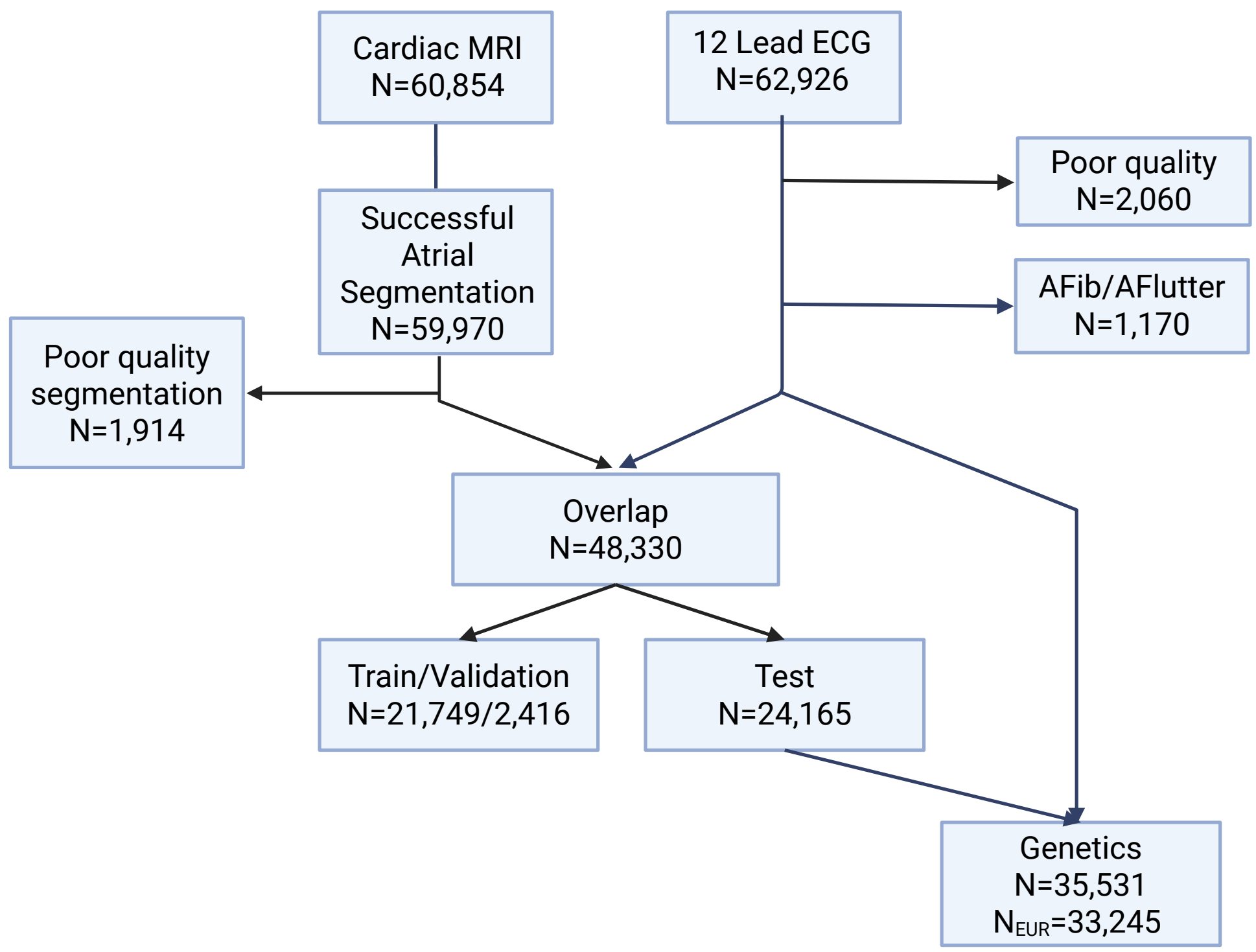

All cardiac MRIs were retrieved from UKB at the initial imaging visit and cardiac structure segmentation was attempted on all participants with imaging files for the short axis, long axis 2-chamber and 4-chamber view. Poor quality segmentation results were removed. All 12-lead ECGs from the same visit were downloaded and poor quality or ECGs with reported codes for atrial fibrillation or flutter were removed. The resulting 48,330 samples with both CMR and ECG were the ECG-AI model dataset. The UKB data were split into 45% training, 5% validation, and 50% testing datasets. Correlations with CMR volumes and UKB outcomes were performed in the Test dataset. Left atrial measures were predicted into the additional ECGs that did not have matching CMR data and were of European ancestry for genetics analyses. Created in BioRender. Brody, J. (2026) https://BioRender.com/d00t542.

Supplementary Fig. 2: Bland–Altman analysis of ECG-AI predicted vs CMR measured left atrial parameters

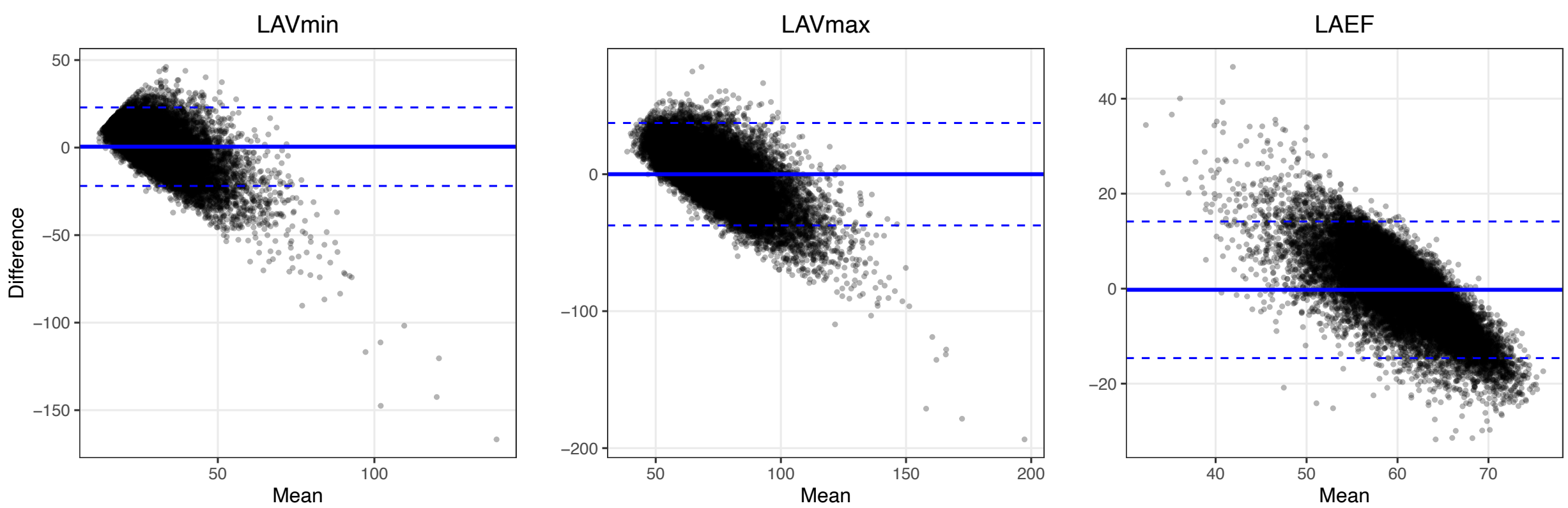

Bland-Altman plots for LAVmin, LAVmax, and LAEF in the UK Biobank test set (N=24,165). The x-axis shows the mean of predicted and measured values; the y-axis shows the difference (predicted minus measured). Solid line: mean difference (bias); dashed lines: 95% limits of agreement (±1.96 SD). Proportional bias is evident, with systematic underestimation at higher values and overestimation at lower values.

Supplementary Fig. 3: Correlation between segmented CMR measures and ECG-AI predicted measures in MESA

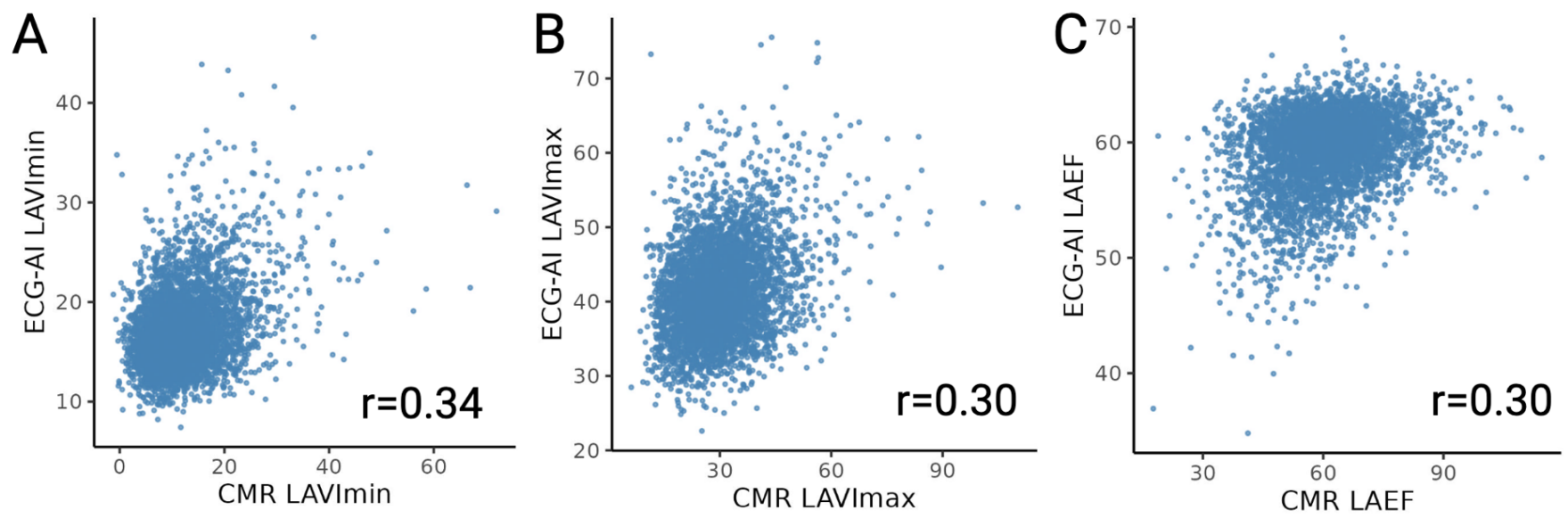

Scatter plots comparing CMR-measured LA minimal volume (LAVmin; n = 4,202), LA maximal vol ume (LAVmax; n = 4,253), and LA ejection fraction (LAEF; n = 4,201) with ECG-AI predictions fro m 12-lead ECGs in MESA.

Supplementary Fig. 4. Learned representation analysis

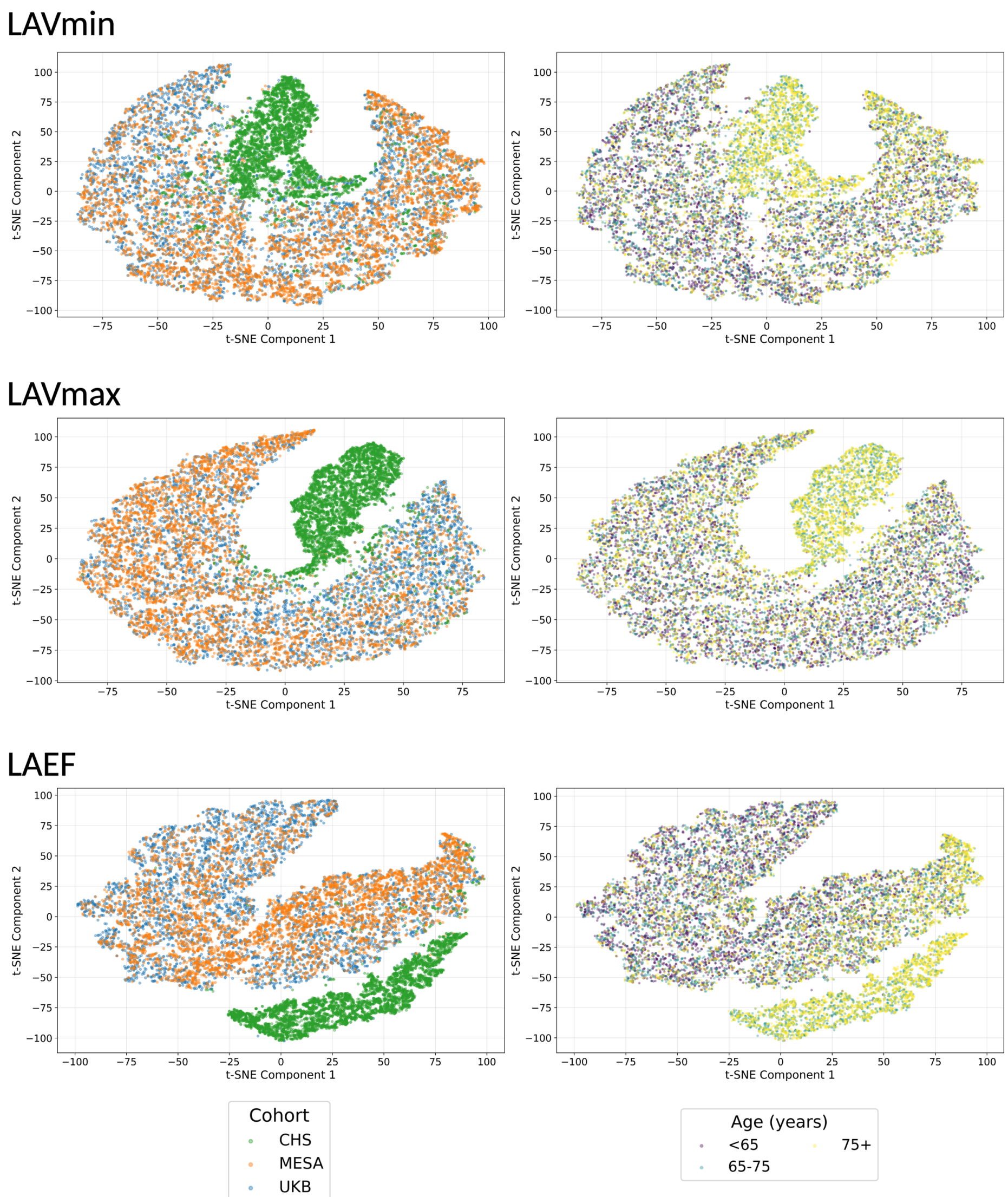

t-SNE visualization of learned representations from ensemble models for LAVmin, LAVmax and LAEF in 36,733 participants (UKB, N = 24,161; MESA, N = 6,766; CHS, N = 5,806). Left panels are colored by cohort: UKB (blue), MESA (orange), and CHS (green). Right panels show the same embeddings colored by age: <65 years (purple), 65-75 years (blue), and >75 years (yellow). UKB and MESA participants overlap extensively despite being independent cohorts with different acquisition systems, demonstrating transportability across populations. CHS participants occupy predominantly distinct regions with scattered intermixing, which aligns with their older age distribution (mean age 72±5 years vs 55±8 years UKB, 62±10 years MESA). Age-matched analysis (70-75 years) revealed CHS remained distinctly separated from UKB/MESA (mean distance: 37.6 units vs 9.9 units between UKB age groups 10 years apart), indicating cohort-specific factors beyond age alone.

Supplementary Fig. 5. Decision curve analysis for risk-guided intensive cardiac monitoring

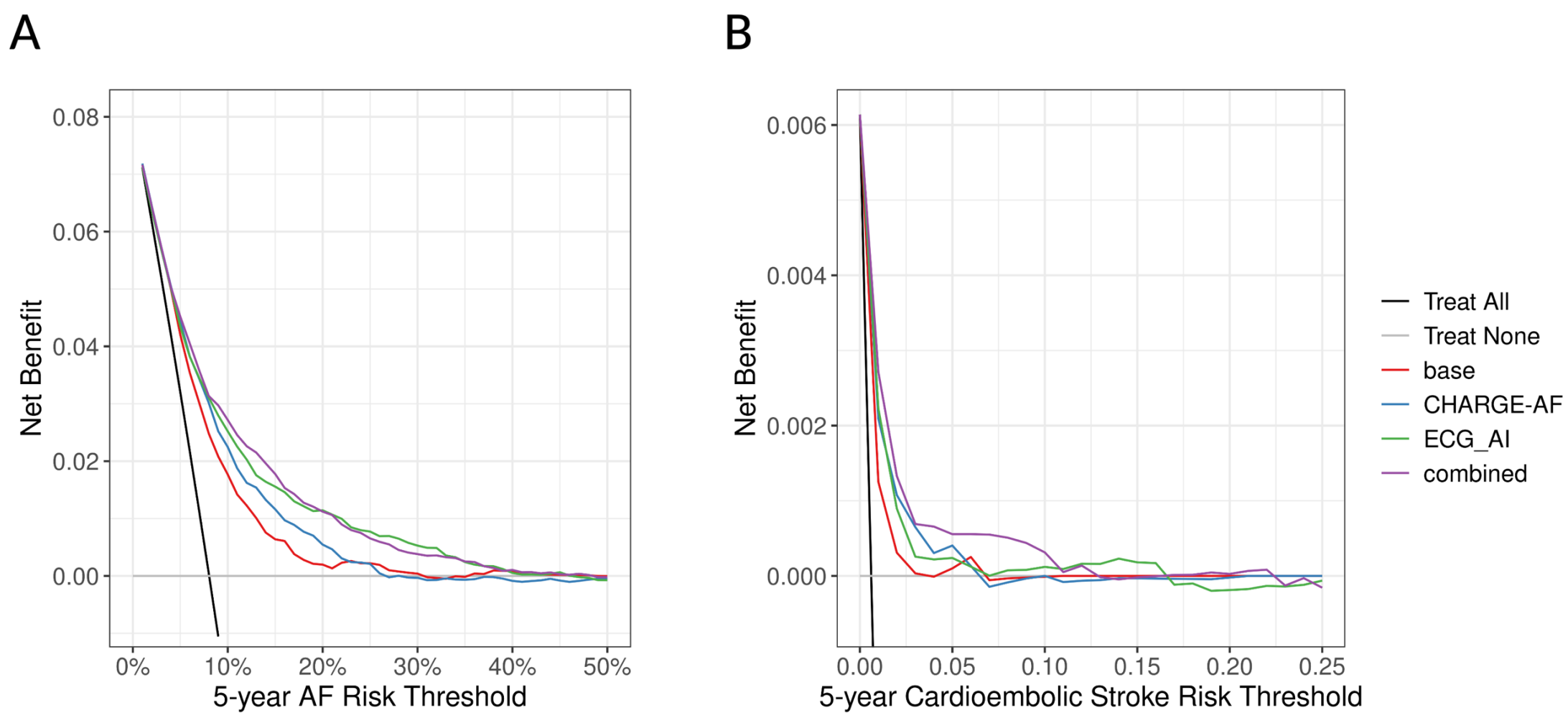

Decision curve analysis of 5-year atrial fibrillation risk in 10,846 participants (842 events) and 5-year cardioembolic stroke risk in 10,642 participants (63 events) from the combined Cardiovascular Health Study and Multi-Ethnic Study of Atherosclerosis cohorts, comparing net benefit of prediction models across risk thresholds. Panel A shows 5-year atrial fibrillation risk, where elevated risk would inform decisions about extended cardiac monitoring. Panel B shows 5-year cardioembolic stroke risk, included to demonstrate outcome specificity. Net benefit is shown for base model (Age+Sex), CHARGE-AF, ECG-AI, and combined model, alongside treat-all and treat-none strategies. For AF, ECG-AI and the combined model show greater net benefit across clinically relevant thresholds. For cardioembolic stroke, differences between models are minimal.

Supplementary Fig. 6: Gradient-weighted Class Activation Mapping of ECG features in left atrial prediction modeling

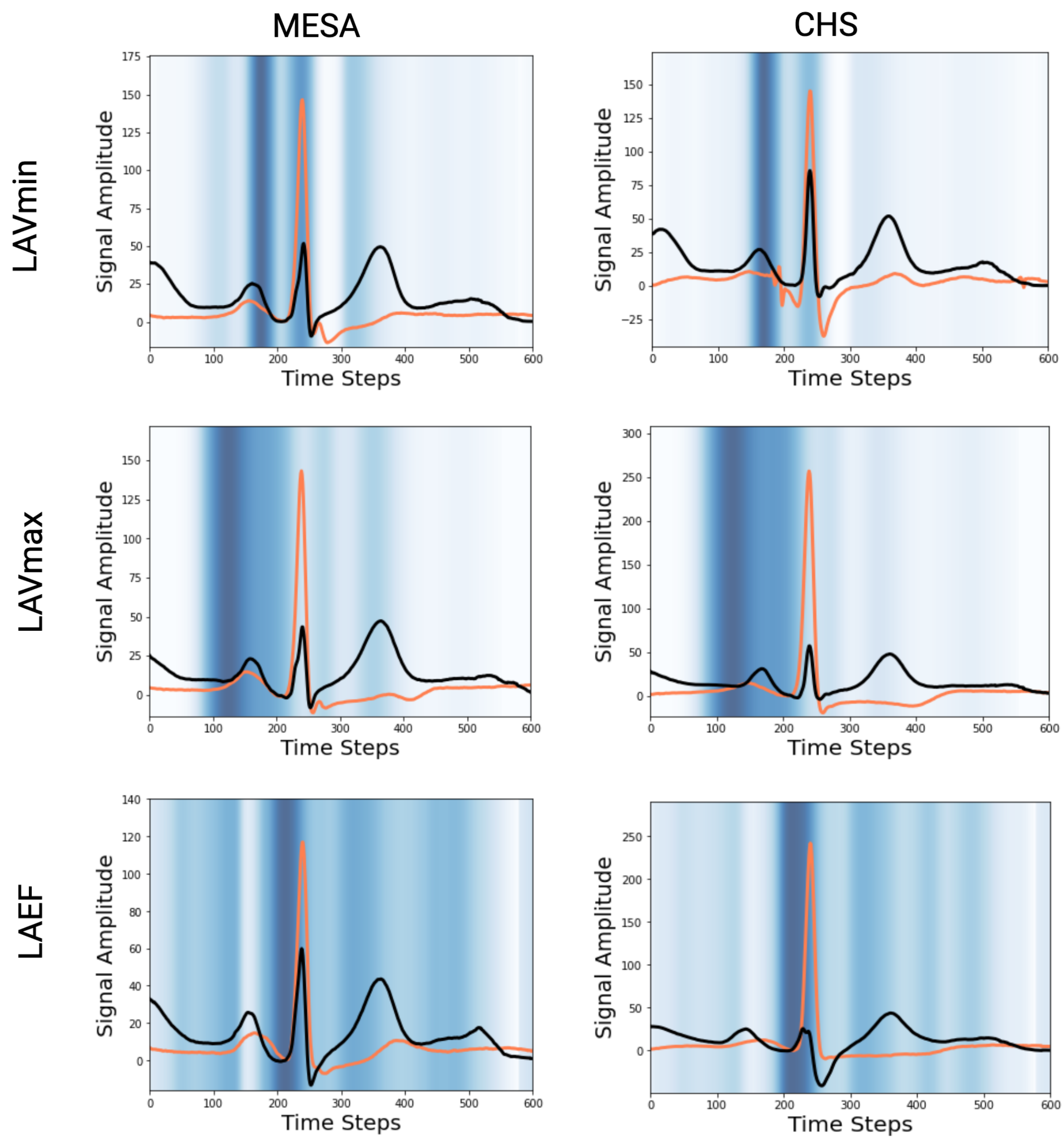

This figure shows the heatmaps for the electrocardiogram (ECG) region driving the predictions of the ECG-AI model across three cohorts. The blue intensity corresponds to feature importance, with the darkest blue signaling the highest importance. The 50 largest volumes (worse) for LAVmin and the 50 smallest ejection fractions for LAEF (worse) median beats are averaged and displayed in orange. For comparison, the average of the 50 smallest (volumes) or largest (LAEF) are plotted in black. For left atrial minimal volume (LAVmin), the model is primarily focused on the decline of the P-wave, while left atrial maximal volume (LAVmax) is focused on the proximal P-wave. In contrast, the left atrial ejection fraction (LAEF) signal is focused on the PQ segment, with consistent P-wave signaling intensities across the three cohort studies. Created in BioRender. Brody, J. (2026) https://BioRender.com/a26r019.

Supplementary Fig. 7: Grad-CAM guided ECG feature ablation and prediction performance

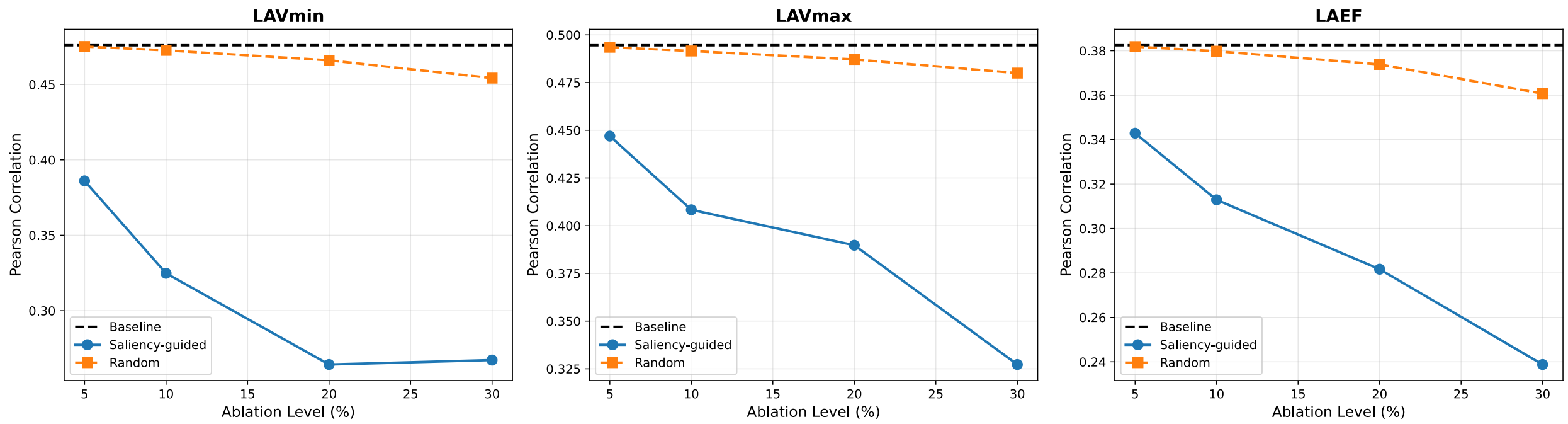

Model performance under progressive ablation of ECG features in UKB (n = 24,165). Features were removed at four levels (5%, 10%, 20%, 30%) using saliency-guided ablation (removing Grad-CAM high-importance regions, red), random ablation (removing random segments, blue), or no ablation (baseline, black). Performance is measured by Pearson correlation on the held-out test set for LAVmin, LAVmax and LAEF. Greater performance degradation with saliency-guided versus random ablation demonstrates that Grad-CAM identifies functionally important ECG regions.

Supplementary Fig. 8. Manhattan plot for ECG-AI predicted LA measures in UKB participants

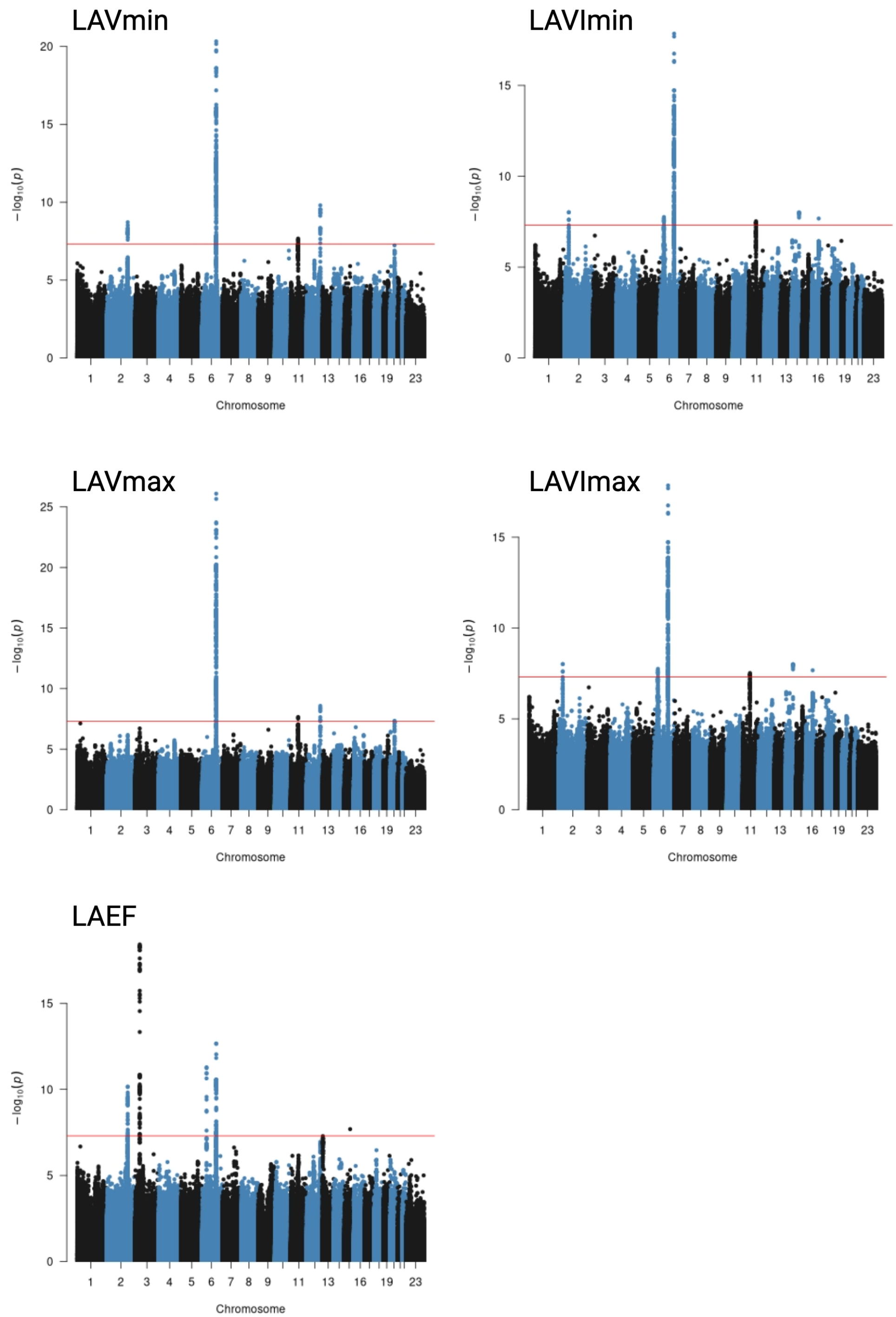

Manhattan plots for genome-wide association studies of ECG-AI predicted left atrial measures in UK Biobank European ancestry participants (N = 33,245). Each point represents a genetic variant. The x-axis represents the genomic chromosomal position, and the y-axis represents is the −log10(p value) of the genetic association. The red horizonal line represents the threshold for significance at 5E-8.
