# Supplementary material for: Deep learning prediction of left atrial structure and function from 12-lead electrocardiograms
Source: Nat Commun. 2026 Jul 31;17:9273. doi: 10.1038/s41467-026-76155-6 (PMC13527101; doi:10.1038/s41467-026-76155-6)
Supplement: Supplementary file 2 [file 41467_2026_76155_MOESM2_ESM.pdf]

Description of Additional supplementary files

Supplementary Data 1: Proportional hazards assumption testing and associations of ECG-AI predicted measures and biomarkers of left atrial function with 5-year clinical outcomes
Cox proportional hazards regression with two-sided p-values; no adjustment for multiple comparisons was made. PH Test P values are from Schoenfeld residual tests. LAEF, left atrial ejection fraction; LAVImax, maximum left atrial volume index; LAVImin, minimum left atrial volume index; PTFV1, P-terminal force in lead V1.

Supplementary Data 2: Age stratified results in MESA
Cox proportional hazards regression with two-sided p-values; no adjustment for multiple comparisons was made. LAEF, left atrial ejection fraction; LAVImax, maximum left atrial volume index; LAVImin, minimum left atrial volume index; PTFV1, P-terminal force in lead V1.

Supplementary Data 3: Sex stratified associations of ECG-AI predicted measures and biomarkers of left atrial function with clinical outcomes
Cox proportional hazards regression with two-sided p-values; no adjustment for multiple comparisons was made. LAEF, left atrial ejection fraction; LAVImax, maximum left atrial volume index; LAVImin, minimum left atrial volume index; PTFV1, P-terminal force in lead V1.

Supplementary Data 4: Association of CMR measured and ECG-AI predicted atrial measures with outcomes in MESA
Cox proportional hazards regression with two-sided p-values; no adjustment for multiple comparisons was made. CMR, cardiac magnetic resonance; LAEF, left atrial ejection fraction; LAVImax, indexed maximum left atrial volume; LAVImin, indexed minimum left atrial volume.

Supplementary Data 5: GWAS index SNPs for ECG-AI traits in UKB
Beta and SE are in units of trait SD. Nearest gene was determined using GENCODE v26. The Atrial Fibrillation SNP column lists any index SNP from Nielsen et al. within 500 kb of the ECG-AI index SNP. Chr, chromosome; LAEF, left atrial ejection fraction; LAVImax, indexed maximum left atrial volume; LAVImin, indexed minimum left atrial volume; LAVmax, maximum left atrial volume; LAVmin, minimum left atrial volume; SE,

standard error; SNP, single nucleotide polymorphism; UKB, UK Biobank.
