# Supplementary material for: Deep learning prediction of left atrial structure and function from 12-lead electrocardiograms
Source: Nat Commun. 2026 Jul 31;17:9273. doi: 10.1038/s41467-026-76155-6 (PMC13527101; doi:10.1038/s41467-026-76155-6)
Supplement: Supplementary file 3 [file 41467_2026_76155_MOESM8_ESM.pdf]

nature portfolio

Corresponding author(s): Jennifer A Brody

Last updated by author(s): Feb 6, 2026

Reporting Summary

Nature Portfolio wishes to improve the reproducibility of the work that we publish. This form provides structure for consistency and transparency in reporting. For further information on Nature Portfolio policies, see our Editorial Policies and the Editorial Policy Checklist.

Statistics

For all statistical analyses, confirm that the following items are present in the figure legend, table legend, main text, or Methods section.

n/a	Confirmed	
☐	☒	The exact sample size (n) for each experimental group/condition, given as a discrete number and unit of measurement
☐	☒	A statement on whether measurements were taken from distinct samples or whether the same sample was measured repeatedly
☐	☒	The statistical test(s) used AND whether they are one- or two-sided<br>*Only common tests should be described solely by name; describe more complex techniques in the Methods section.*
☐	☒	A description of all covariates tested
☐	☒	A description of any assumptions or corrections, such as tests of normality and adjustment for multiple comparisons
☐	☒	A full description of the statistical parameters including central tendency (e.g. means) or other basic estimates (e.g. regression coefficient) AND variation (e.g. standard deviation) or associated estimates of uncertainty (e.g. confidence intervals)
☐	☒	For null hypothesis testing, the test statistic (e.g. F, t, r) with confidence intervals, effect sizes, degrees of freedom and P value noted<br>*Give P values as exact values whenever suitable.*
☒	☐	For Bayesian analysis, information on the choice of priors and Markov chain Monte Carlo settings
☐	☒	For hierarchical and complex designs, identification of the appropriate level for tests and full reporting of outcomes
☐	☒	Estimates of effect sizes (e.g. Cohen's d, Pearson's r), indicating how they were calculated

Our web collection on statistics for biologists contains articles on many of the points above.

Software and code

Policy information about availability of computer code

Data collection	ECG data: CASE® CardioSoft® Version 6 (GE Healthcare) for UK Biobank; MAC PC ECG Machines with GE 12-SL software running under GE MUSE and Magellan Research Work Station for MESA and CHS. CMR imaging: 1.5 Tesla scanner (MAGNETOM Aera, Syngo Platform VD13A, Siemens Healthcare) for UK Biobank; 1.5-T scanners with ECG-gated fast gradient echo pulse sequence (baseline) and steady-state free precession (Exam 5) for MESA. Left atrial segmentation: Publicly available fully convolutional network pipeline by Bai et al. (2018), available on GitHub (https://github.com/baiwenjia/ukbb_cardiac). Echocardiography strain analysis in CHS: TOMTEC v4.5 speckle-tracking software.
Data analysis	Deep learning model: Custom convolutional neural network (inception architecture) implemented in Python using TensorFlow/Keras. Statistical analyses: R version [4.3.1] using packages including survival (Cox models), GENESIS (GWAS linear mixed models), LDSC v1.0.1 (genetic correlations). Gradient-weighted Class Activation Mapping and learned representation analysis performed in python 3.9.23. Code for implementing the ECG-AI model is available on a GitHub repository: https://github.com/j-brody/ecg_ai_atrialmeasures.

For manuscripts utilizing custom algorithms or software that are central to the research but not yet described in published literature, software must be made available to editors and reviewers. We strongly encourage code deposition in a community repository (e.g. GitHub). See the Nature Portfolio guidelines for submitting code & software for further information.

Data

Policy information about availability of data

All manuscripts must include a data availability statement. This statement should provide the following information, where applicable:

- Accession codes, unique identifiers, or web links for publicly available datasets
- A description of any restrictions on data availability
- For clinical datasets or third party data, please ensure that the statement adheres to our policy

The data supporting the findings from this study are available within the manuscript and its supplementary information. UK Biobank data are made available to researchers from research institutions as described at this site: https://www.ukbiobank.ac.uk/enable-your-research. Data for Multi-Ethnic Study of Atherosclerosis and Cardiovascular Health Study can be requested at https://internal.mesa-nhlbi.org/ and https://chs-nhlbi.org/node/6222, respectively. GWAS summary statistics are publicly available under accession numbers GCST006414 [https://www.ebi.ac.uk/gwas/studies/GCST006414] (atrial fibrillation), GCST90104540 [https://www.ebi.ac.uk/gwas/studies/GCST90104540] (ischemic stroke), GCST90104541 [https://www.ebi.ac.uk/gwas/studies/GCST90104541] (cardioembolic stroke), GCST90104542 [https://www.ebi.ac.uk/gwas/studies/GCST90104542] (large artery stroke), GCST90104543 [https://www.ebi.ac.uk/gwas/studies/GCST90104543] (small vessel stroke), GCST90132314 [https://www.ebi.ac.uk/gwas/studies/GCST90132314] (coronary artery disease), GCST90239658 [https://www.ebi.ac.uk/gwas/studies/GCST90239658] (low density lipoprotein cholesterol), GCST90239652 [https://www.ebi.ac.uk/gwas/studies/GCST90239652] (high density lipoprotein cholesterol), GCST90239664 [https://www.ebi.ac.uk/gwas/studies/GCST90239664] (triglycerides), GCST90310294 [https://www.ebi.ac.uk/gwas/studies/GCST90310294] (systolic blood pressure), GCST90310295 [https://www.ebi.ac.uk/gwas/studies/GCST90310295] (diastolic blood pressure), GCST009004 [https://www.ebi.ac.uk/gwas/studies/GCST009004] (BMI) and GCST010320 [https://www.ebi.ac.uk/gwas/studies/GCST010320 (PR interval) at https://www.ebi.ac.uk/gwas/home. The GWAS summary statistics for heart failure [https://cvd.hugeamp.org/dinspector.html?dataset=Shah2020_HF_EU], type 2 diabetes [https://www.diagram-consortium.org/downloads.html], and QT interval [https://www.arkinglab.org/resources/] are available at their respective repositories.

Research involving human participants, their data, or biological material

Policy information about studies with human participants or human data. See also policy information about sex, gender (identity/presentation), and sexual orientation and race, ethnicity and racism.

Reporting on sex and gender	Sex was determined by self-report at baseline assessment in all three cohorts (UK Biobank, MESA, CHS). Sex was included as a covariate in all multivariable Cox proportional hazards models. All analyses included both sexes. The term "sex" refers to biological sex as reported by participants; gender was not assessed. Sex-stratified analyses are reported in Supplementary table 7.
Reporting on race, ethnicity, or other socially relevant groupings	Race and ethnicity were self-reported at baseline and included as covariates in all adjusted models to account for social and environmental factors. In MESA, race/ethnicity categories were White, African American, Hispanic, or Chinese American as defined by the study protocol. In UK Biobank and CHS, race was categorized as White or other due to limited racial diversity in these cohorts. Race was included as a covariate to control for confounding by social, environmental, and healthcare access factors associated with racial categories. Race was not used as a proxy for biological or genetic differences.
Population characteristics	Baseline characteristics for all three cohorts are provided in Supplementary Tables 1-2, including age, sex, race/ethnicity, body mass index, systolic and diastolic blood pressure, smoking status, prevalent diabetes mellitus, heart failure, myocardial infarction, medication use (antihypertensive medications), and lipid levels. Training, validation, and test samples in UK Biobank showed balanced characteristics (Supplementary Table 1). Mean ages were 65 years in UKB, 62 years in MESA, and 73 years in CHS. The UK Biobank training sample was 53% female with low prevalence of heart failure (0.4%) and prior myocardial infarction (2.1%). Genotypic information is described in the Genome-wide Association Studies methods section.
Recruitment	UK Biobank recruited 500,000 participants aged 40-69 years from the United Kingdom between 2006-2010 through postal invitation. The imaging substudy (2014-2023) included participants who returned for a second assessment visit and consented to CMR imaging, which may represent a healthier subset of the overall UK Biobank population. MESA recruited 6,814 adults aged 45-84 years free of clinical cardiovascular disease from four racial/ethnic groups at six U.S. field centers in 2000-2002. CHS recruited 5,888 adults aged ≥65 years from random samples of Medicare eligibility lists at four U.S. field centers (1989-1990 and 1992-1993). All cohorts relied on volunteer participation, which may introduce healthy volunteer bias and limit generalizability to individuals with more advanced disease or lower willingness to participate in research.
Ethics oversight	UK Biobank received ethics approval from the North West Multi-Centre Research Ethics Committee; all participants provided electronic informed consent at UK Biobank assessment centers. MESA was approved by institutional review boards of all six field centers; all participants gave written informed consent. CHS was approved by institutional review boards of all four participating sites; all study participants provided informed consent.

Note that full information on the approval of the study protocol must also be provided in the manuscript.

Field-specific reporting

Please select the one below that is the best fit for your research. If you are not sure, read the appropriate sections before making your selection.

☒ Life sciences ☐ Behavioural & social sciences ☐ Ecological, evolutionary & environmental sciences

For a reference copy of the document with all sections, see nature.com/documents/nr-reporting-summary-flat.pdf

Life sciences study design

All studies must disclose on these points even when the disclosure is negative.

Sample size	No formal sample size calculation was performed. Sample sizes were determined by data availability: UK Biobank N=48,300 with concurrent ECG and CMR passing quality control, randomly split 45% training (N=21,749), 5% validation (N=2,416), 50% test (N=24,165). External validation used existing cohorts: MESA (N=6,814) and CHS (N=5,888).
Data exclusions	Pre-established exclusion criteria applied before analysis. ECG exclusions (N=3,230 total): poor quality (N=1,483), suspect lead reversal (N=429), atrial fibrillation/flutter (N=1,251), pacemaker (N=174), other quality issues (N=313). CMR exclusions: LA minimal volume ≤8 mL, LA maximal volume ≤24 mL, LA ejection fraction >86%, validated by visual inspection (>95% accuracy). Participants with prevalent disease were excluded from corresponding outcome analyses. Details in Supplementary Figure 1.
Replication	ECG-AI model developed in UK Biobank was externally validated without retraining in two independent cohorts (MESA, CHS). Key findings replicated with consistent effect sizes: AF associations (LAVImin HR 1.37 UKB, 1.34 MESA, 1.41 CHS), heart failure (HR 1.42 UKB, 1.36 MESA, 1.45 CHS), cardioembolic stroke (HR 1.16 UKB, 1.16 MESA, 1.22 CHS). Model performance was lower in external cohorts (r=0.30-0.34) than development cohort (r=0.50), as expected for external validation.
Randomization	Not applicable to observational study design. UK Biobank participants were randomly allocated to training (45%), validation (5%), and test (50%) sets for model development, with balanced baseline characteristics (Supplementary Table 1). Allocation performed once and remained fixed throughout analyses.
Blinding	Not applicable. ECG-AI predictions were generated algorithmically without human interpretation. Clinical outcomes were ascertained independently through medical records, adjudication committees (MESA, CHS), and national health databases (UK Biobank).

Reporting for specific materials, systems and methods

We require information from authors about some types of materials, experimental systems and methods used in many studies. Here, indicate whether each material, system or method listed is relevant to your study. If you are not sure if a list item applies to your research, read the appropriate section before selecting a response.

Materials & experimental systems

n/a	Involved in the study
☒	☐ Antibodies
☒	☐ Eukaryotic cell lines
☒	☐ Palaeontology and archaeology
☒	☐ Animals and other organisms
☒	☐ Clinical data
☒	☐ Dual use research of concern
☒	☐ Plants

Methods

n/a	Involved in the study
☒	☐ ChIP-seq
☒	☐ Flow cytometry
☒	☐ MRI-based neuroimaging

Plants

Seed stocks	*Report on the source of all seed stocks or other plant material used. If applicable, state the seed stock centre and catalogue number. If plant specimens were collected from the field, describe the collection location, date and sampling procedures.*
Novel plant genotypes	*Describe the methods by which all novel plant genotypes were produced. This includes those generated by transgenic approaches, gene editing, chemical/radiation-based mutagenesis and hybridization. For transgenic lines, describe the transformation method, the number of independent lines analyzed and the generation upon which experiments were performed. For gene-edited lines, describe the editor used, the endogenous sequence targeted for editing, the targeting guide RNA sequence (if applicable) and how the editor was applied.*
Authentication	*Describe any authentication procedures for each seed stock used or novel genotype generated. Describe any experiments used to assess the effect of a mutation and, where applicable, how potential secondary effects (e.g. second site T-DNA insertions, mosiacism, off-target gene editing) were examined.*
