# Supplementary material for: Deep learning prediction of left atrial structure and function from 12-lead electrocardiograms
Source: Nat Commun. 2026 Jul 31;17:9273. doi: 10.1038/s41467-026-76155-6 (PMC13527101; doi:10.1038/s41467-026-76155-6)
Supplement: Supplementary file 4 [file 41467_2026_76155_MOESM9_ESM.pdf]

nature portfolio

Peer Review File

Deep Learning Prediction of Left Atrial Structure and Function from 12-lead Electrocardiograms

Corresponding Author: Ms Jennifer Brody

This file contains all reviewer reports in order by version, followed by all author rebuttals in order by version.

Version 0:

Reviewer comments:

Reviewer #1

(Remarks to the Author)
Following concerns need to be addressed:

1. Authors referenced to only a few studies applying artificial intelligence (AI) and deep learning (DL) models. Furthermore, they did not describe their criteria and rationale to select these studies.

2. Authors did not describe their own rationale for using DL approach, and even other techniques used for data analysis.

3. Novelty is not clear.

4. They used human genomic data, is the data used in this study from the same participants? Furthermore, details about genomic data processing and analysis are not well addressed.

5. The biggest concern is the validation and authenticity of results, and their clinical acceptance and translational impact.

6. Some minor comments, data analysis can be better planned and presented. Better choice of figures can be made for clear presentation and interpretation. Method's reproducibility and transparency can be better explained.

(Remarks on code availability)
There must be guidelines in accessing data, source code, and execution. In current form, ReadMe is not clear and self explanatory for the readers to exercise.

Reviewer #2

(Remarks to the Author)
1. The model is trained on cardiac MRI labels generated by an automated segmentation algorithm rather than on manually annotated ground truth. The authors do not quantify segmentation error, assess uncertainty propagation, or validate model performance against a manually labeled subset. Without this, it is difficult to determine whether the ECG model is learning true left atrial structure or reproducing artifacts of the segmentation pipeline.

2. The procedures for ECG preprocessing and exclusion are insufficiently documented. The manuscript does not report how many ECGs were removed due to quality issues, arrhythmias, lead reversal, or automated flags. Because the excluded ECGs likely come from individuals with more advanced pathology, this may bias the training population and reduce the model's ability to generalize to sicker patients.

3. The dataset split of 45% train, 5% validation, and 50% test is unconventional and may reduce the effective training data for a high capacity model. The authors do not describe stratification, nor do they address how individuals in the UKBB were separated across splits.

4. The evaluation of the core regression task is incomplete. The model's accuracy is presented using (weak) correlations

only. Absolute error metrics such as RMSE or MAE are not reported, and no calibration analyses are shown. Without these, it is unclear whether predicted volumes and ejection fraction values are sufficiently accurate to serve as surrogates for MRI measurements.

5. The outcome modeling framework lacks several important statistical diagnostics. The proportional hazards assumption is not tested, competing risks are not modeled, and multiple hypothesis testing is not addressed despite numerous comparisons across cohorts, outcomes, and left atrial metrics. These omissions weaken the validity of the reported associations.

6. The subclinical atrial fibrillation screening experiment is limited by a six year gap between the baseline ECG and the 14 day monitor. Structural remodeling and incident AFib may have occurred during this period, making the analysis unsuitable for evaluating near term screening potential. Additionally, only a subset of participants underwent monitoring, but the authors do not analyze potential selection bias.

7. The harmonization of ECG data across devices, acquisition systems, and cohorts is not adequately described. The authors mention voltage rescaling but do not address any other domain shift issues. Without explicit analyses, claims of transportability remain tentative.

8. I'm not entirely sold on the utilization of one "median" beat. What exactly is the gain here?
8a. And just to be absolutely certain, can the authors explicitly state whether the beat splitting was done after individual cohort level splitting s.t. each ECG yields exactly one data instance? Furthermore, are we absolutely certain that repeat ECGs from a single person were not used in different cohorts?

9. The manuscript sometimes blends evaluation of prediction accuracy with evaluation of prognostic association. Separating these more cleanly would help readers interpret what the model is and is not demonstrating.

10. The handling of missing clinical covariates in survival models is not described. Whether the authors performed complete case analysis, imputation, or indicator modeling is unclear. This is especially relevant for proBNP, where missingness is common. If it's not common, we're likely overselecting from one cohort of patients.

11. The model architecture is described at a high level but omits important hyperparameters such as kernel sizes, filter counts, and the exact search space for the random hyperparameter search.

12. The Grad CAM visualization is based on extreme examples and is not validated with quantitative perturbation or ablation analyses. Given the limitations of saliency methods for one dimensional signals, I'm not sure the claims related to explainability within the manuscript are justified.

(Remarks on code availability)
A key issue affecting both correctness and reproducibility is that the released inference code does not implement the full preprocessing pipeline described in the manuscript. The paper's methods emphasize that each ECG was transformed into a standardized 600 sample median beat derived from ten seconds of raw data, with explicit harmonization of mv scaling across systems. The provided code, however, accepts preprocessed 600x8 NumPy arrays without offering any mechanism to compute the median beat, align cardiac cycles, resample the waveform, or parse raw ECG formats. Consequently, users cannot reproduce the model's inputs from raw ECG data using the released code alone, limiting the reproducibility of the core methodological pipeline.

Additionally, the script does not document the units or scale of the model outputs. The paper indicates that atrial volumes were indexed to body surface area and may have been standardized prior to modeling, yet the inference code emits raw prediction values without specifying whether they correspond to physical volumes, indexed measures, or z-scores. Without explicit post-processing or metadata accompanying the pretrained models, users cannot interpret the resulting LAVmin, LAVmax, or LAEF values in the same units used in the manuscript. This omission directly impairs reproducibility of any downstream analysis based on the predictions.

As such, the provided code isn't very helpful without at least a representative data sample to go alongside it.

Reviewer #3

(Remarks to the Author)
This manuscript uses paired CMR and 12-lead ECG data from the UK Biobank to train deep learning models that predict left atrial (LA) structure and functon(LAVmin, LAVmax, LAEF), and then evaluates these “ECG-AI” traits in UKB, MESA and CHS for associations with incident atrial fibrillation (AF), heart failure (HF) and stroke. The authors further compare ECG-AI with conventional ECG and imaging markers and with CHARGE-AF, build 5-year prediction models for AF and cardioembolic stroke, analyze a MESA patch-monitoring sub study for subclinical AF, and add Grad-CAM and GWAS analyses for interpretability. The overall question, whether a deep learning model applied to standard ECG can approximate LA phenotypes and serve as a clinically useful risk marker, is timely and important. The cohorts and outcome adjudication are strong. However, the internal characterization of the models, the framing of external validation and comparative performance, and the interpretation of several exploratory analyses require tightening.

1. Better quantify internal model performance and label quality

In UKB, the authors train “inception-style” 1D CNNs on an 8×600 median-beat representation of the 10-second ECG to predict CMR-derived LAVmin, LAVmax and LAEF, using a 45/5/50% patient-level split. The main internal metric reported is the Pearson correlation in the hold-out test set (r≈0.50 for LAVmin/LAVmax and r≈0.40 for LAEF). While this demonstrates a moderate relationship between ECG-AI and CMR, correlation alone is not sufficient to judge whether the predictions are accurate and unbiased enough to be treated as surrogate LA traits in downstream analyses. The manuscript does not provide MAE/RMSE in the test set, show error behavior at small versus markedly enlarged LA volumes, or present basic bias plots. Given that these continuous ECG-AI traits are later used as exposures in Cox models and in risk prediction, the reader needs a concise description of absolute error and error structure, ideally stratified across the range of LA size and function.

In addition, all LA labels come from an automated segmentation pipeline. The authors refer to prior validation, but there is no summary—either from that work or from a quality-control subsample in the present study—of segmentation performance for LA volumes and LAEF in this dataset. A short description of segmentation error and reproducibility would strengthen confidence in the labels and, by extension, in the regression models.

2. Explicitly acknowledge limited power of UK Biobank stroke analyses

In the UKB test set, both CMR-indexed LA traits and their ECG-AI counterparts are related to incident AF, HF, and ischemic stroke over a median of roughly four years, with adjustment for age, sex, and conventional risk factors. The directions and magnitudes of association for CMR LAVImin/LAVmax and LAEF are consistent with prior literature, and ECG-AI traits show broadly similar hazards for AF and, in some models, even stronger associations with HF. These results support the idea that ECG-AI captures much of the risk-relevant signal encoded in LA structure and function.

By contrast, the UKB stroke analysis is clearly underpowered: there are only 71 ischemic stroke events, and hazard ratios are not statistically significant. More definitive conclusions on stroke risk are rightly drawn from MESA and CHS, which have more events and formal subtype adjudication. To avoid over-interpreting the UKB data, it would improve clarity if the authors explicitly stated that the UKB stroke findings are directional only and that substantive inferences about stroke—particularly cardioembolic stroke—are based on the external cohorts.

3. Moderate mechanistic claims that ECG-AI is “beyond imaging”

The external validation in MESA and CHS is a major strength. Applying the UKB-trained models without retraining, the authors show that cross-sectional correlations between ECG-AI and LA imaging traits are lower than in UKB (r≈0.30–0.34), yet associations with AF, HF and ischemic/cardioembolic stroke remain robust and often similar in magnitude to those in UKB after multivariable adjustment. This pattern—attenuated correlation with imaging but preserved outcome association—supports the robustness of ECG-AI as a risk marker across cohorts with different imaging modalities and protocols.

However, the manuscript currently tends to interpret these findings as evidence that ECG-AI captures information “beyond imaging.” While this is possible, it is not required by the data: differences in modality (CMR vs echocardiography), acquisition and measurement error between cohorts can readily explain the drop in cross-sectional correlation, and outcome associations reflect not only LA structure but also broader risk profiles and surveillance intensity. I would suggest using more conservative wording that emphasizes ECG-AI as a transportable risk marker which performs comparably to, and in some settings as well as or modestly better than, imaging-based measures, without implying a strong mechanistic claim that it biologically surpasses imaging.

4. Clarify the incremental value over conventional markers and risk scores

The comparative analyses with PTFV1, LA strain and CHARGE-AF are important for clinical positioning. It is plausible and well supported by the data that ECG-AI offers stronger risk associations than PTFV1 and LA strain in MESA and CHS, given that it uses the full waveform rather than a single summary metric. The observation that ECG-AI correlates more strongly with log NT-proBNP than do CMR LA measures, and that adjustment for NT-proBNP attenuates but does not eliminate ECG-AI associations with AF, HF, and stroke, is also informative: it suggests that the model integrates a mix of LA and global hemodynamics/load information rather than representing a purely structural LA marker.

For the 5-year prediction models, ECG-AI alone reaches similar AUROC to CHARGE-AF and slightly higher AUPRC and adding ECG-AI to CHARGE-AF yields small but statistically significant improvements in discrimination (ΔAUROC on the order of 0.02–0.04). The analysis itself is well designed. However, expressions such as “outperformed clinical risk scores” are stronger than the effect sizes support. Without calibration plots, Brier scores, reclassification metrics (e.g., NRI/IDI) or decision-curve analyses, it is difficult to judge whether these improvements would meaningfully change clinical decisions. I would recommend moderating the language and, if feasible, adding at least a brief calibration and/or reclassification assessment to quantify the incremental value of ECG-AI over established clinical scores.

5. Re-frame the MESA subclinical AF analysis as exploratory

The MESA patch-monitoring sub study addresses a clinically attractive use case: using ECG-AI to enrich for individuals in whom 14-day monitoring is likely to detect AF. The finding that upper-quintile ECG-AI LAVImin groups have higher positive predictive value and capture a larger share of detected AF cases than corresponding CHARGE-AF strata is encouraging and points toward a potential screening application.

Nevertheless, the mean six-year gap between baseline ECG and monitoring, the reliance on a single 14-day monitoring period, and the small number of AF cases (86) substantially limit the strength of inference. As currently written, this section reads close to a practical screening proposal. It would be more appropriate to describe this analysis explicitly as exploratory and hypothesis-generating, and to emphasize that prospective studies with contemporaneous ECG-AI assessment and rhythm monitoring will be needed before ECG-AI can be recommended as a tool to select patients for extended monitoring.

In summary, this is a well-designed and potentially impactful study that uses high-quality imaging–ECG pairings and large, well-phenotype cohorts to address an important question at the intersection of ECG-based AI, LA remodeling and cardioembolic risk. Its main strengths are the same-day CMR labels, multi-cohort validation and coherent outcome analyses.

The main weaknesses are the limited internal characterization of the regression models and the over-interpretation of several exploratory analyses, including some mechanistic claims. With a more complete description of model error and label performance, more cautious and quantitatively grounded comparative claims, and clearer framing of the subclinical AF and genetics components as exploratory.

(Remarks on code availability)

Reviewer #4

(Remarks to the Author)
This well-conducted study by Brody and colleagues presents a deep learning framework (ECG-AI) to predict left atrial (LA) structure and function from the 12-lead ECG, using cardiac magnetic resonance imaging (CMR) as the gold standard. The model was trained on over 48,000 ECG–CMR pairs from UK Biobank and externally validated in two large, well-characterized cohort studies (MESA and CHS). The ECG-AI–derived measures of LA volume and ejection fraction (LAEF) correlated moderately with CMR-derived parameters. They were significantly associated with incident atrial fibrillation (AF), heart failure (HF), and ischemic stroke. Significantly, the model outperformed traditional clinical risk models, such as CHARGE-AF, and demonstrated potential utility for screening for subclinical AF.
The study's key strengths include: 1) Leveraging the gold-standard CMR for model training, ensuring a robust reference for LA structure and function; 2) Using the extensive UK Biobank dataset, external validation across two independent cohorts with multiple outcomes (AF, HF, and stroke); 3) Demonstrating that ECG-AI metrics outperform both imaging-derived measures and clinical risk factors for predicting cardiovascular outcomes emphasizes the translational novelty of the approach; 4) The inclusion of model interpretability analyses (Gradient-weighted Class Activation Mapping [Grad-CAM]) adds important physiological plausibility by localizing the predictive signal to the P-wave and PQ-segment intervals.
From a genetic standpoint, the authors perform genome-wide association studies (GWAS) on five ECG-AI–derived phenotypes (indexed and non-indexed LA volumes and LAEF) among 33,000 UK Biobank participants of European ancestry. Twelve loci were identified across the five traits, including a leading signal near PLN (rs11153730), a locus previously linked to ECG traits, HF, and AF. The authors suggest that these genetic findings, along with modest heritability estimates (h^2 = 0.14–0.17), provide insight into the underlying biological mechanisms of AI-derived traits. While this integration of genomics is conceptually valuable, several vital limitations temper the strength of these conclusions.
Major Comments
1. Interpretation of the genetic analysis requires caution: The authors claim that their genetic analysis "characterized the biological basis" of the ECG-AI phenotypes. However, the identified loci only partially overlap with known AF loci, and heritability estimates are modest (h^2 0.14–0.17). The PLN variant, while biologically plausible, is already well known as a pleiotropic locus associated with multiple ECG and cardiac traits. The mechanistic link between this variant and LA structure/function is not explored, and thus, the assertion that the GWAS elucidates the biology of AI-derived phenotypes is overstated. The authors should clearly delineate that these findings are preliminary and largely hypothesis-generating.
2. Limited genetic overlap with AF: Roughly half of the identified loci did not coincide with established AF loci. This suggests that ECG-AI phenotypes capture overlapping but distinct biological pathways, possibly reflecting general atrial or ventricular remodeling rather than AF-specific substrate. The authors should explicitly discuss this distinction.
3. Population ancestry and representativeness: The UK Biobank training set is predominantly of European ancestry and healthier than the general population, and the GWAS was restricted to European participants. The validation cohorts (MESA, CHS) improved ethnic diversity, but not for the genetic analysis, limiting generalizability. This should be acknowledged.
4. Validation cohort limitations: CHS consists exclusively of adults aged ≥65 years, which restricts applicability to younger populations. The authors should clarify whether predictive performance differed by age or cohort (younger MESA participants vs. older CHS participants).
5. Moderate correlation with imaging-derived measures: Correlations between ECG-AI and CMR-derived LA parameters were only moderate (r = 0.4–0.5 in UKB; r = 0.3–0.34 in external cohorts). The authors should comment on how this level of concordance supports mechanistic validity.
6. Incremental predictive value over existing tools: Although ECG-AI modestly improved discrimination over CHARGE-AF (ΔAUC ≈ 0.02), the clinical implications of this incremental gain are unclear. Reporting net reclassification improvement or decision-curve analysis would help quantify added value.
7. Subclinical AF detection: The subclinical AF analysis relied on 14-day monitoring approximately six years after baseline. This limited window may underestimate the true AF incidence, particularly for paroxysmal cases. The authors should acknowledge that longer-term monitoring would provide a more accurate benchmark.
8. Biological interpretation of AI-derived traits: While Grad-CAM analyses showed that predictive regions align with physiologic atrial activity (P-wave, PQ-segment), it remains unclear whether the ECG-AI measures capture atrial remodeling distinct from ventricular or systemic predictors (e.g., BMI, hypertension). This distinction should be further discussed.

(Remarks on code availability)
I am not qualified to comment on the assessment of the code.

Version 1:

Reviewer comments:

Reviewer #2

(Remarks to the Author)
Thank you for the thorough and constructive revision. The authors have been responsive to my comments, and the manuscript appears substantially improved / stronger in several important respects.

My main concerns have therefore been addressed. That said, the following points would still benefit from minor revision in the final manuscript.
I. Limitations around label quality should be stated as plainly as possible: although the authors provide supporting information on segmentation performance and quality control, the study still does not directly validate the ECG model against a manually labeled subset generated within the present analysis.
II. the 14 day monitoring analysis should remain explicitly framed as exploratory, and the possibility of selection bias in the monitored subset should be acknowledged clearly in the text.
III. Claims regarding transportability and biological interpretation should remain calibrated to the level of direct validation provided, particularly in cohorts where left atrial labels were not available for direct accuracy assessment.

(Remarks on code availability)

Reviewer #3

(Remarks to the Author)
The authors have responsed my comments, There are no further comments.

(Remarks on code availability)

Reviewer #4

(Remarks to the Author)
The authors have addressed the prior critiques thoughtfully and, in doing so, have further strengthened the manuscript. Overall, this is a rigorous, high-impact, and conceptually innovative study. Notably, it represents the first model to leverage the 12-lead ECG to predict left atrial structural and functional measures that are independently associated with cardiovascular complications.

(Remarks on code availability)
N/A

Open Access This Peer Review File is licensed under a Creative Commons Attribution 4.0 International License, which permits use, sharing, adaptation, distribution and reproduction in any medium or format, as long as you give appropriate credit to the original author(s) and the source, provide a link to the Creative Commons license, and indicate if changes were made.

In cases where reviewers are anonymous, credit should be given to 'Anonymous Referee' and the source.
The images or other third party material in this Peer Review File are included in the article's Creative Commons license, unless indicated otherwise in a credit line to the material. If material is not included in the article's Creative Commons license and your intended use is not permitted by statutory regulation or exceeds the permitted use, you will need to obtain permission directly from the copyright holder.
To view a copy of this license, visit https://creativecommons.org/licenses/by/4.0/

REVIEWER COMMENTS

Reviewer #1 (Remarks to the Author):

1. Authors referenced to only a few studies applying artificial intelligence (AI) and deep learning (DL) models. Furthermore, they did not describe their criteria and rationale to select these studies.

In the introduction, we focused on the largest studies that evaluated AF risk prediction using an ECG-model. Studies focused on research specific cohorts (e.g., those with CLL or PFOs) were not included. As there has been a surge in the interest in this topic in the last few years, we selected studies that achieved the highest-performance levels (AUC > 0.80) and have been widely cited. A prior citation has been exchanged to reflect this rationale.

2. Authors did not describe their own rationale for using DL approach, and even other techniques used for data analysis.

We have added a sentence to the Introduction explaining our rationale for using deep learning. It now reads "Recent studies have applied artificial intelligence (AI) and deep learning models to the resting 12-lead electrocardiogram (ECG), which has moderate accuracy in predicting short-and long-term risks of AF.[1-4] Building on the demonstrated success of deep learning approaches in predicting AF from ECG waveforms, we hypothesized that similar methods could extract information about underlying atrial structure and function when trained on paired ECG and imaging data."

3. Novelty is not clear.

The manuscript has undergone significant restructuring to better emphasize the novelty of our approach. The majority of existing models have evaluated the prediction of incident atrial fibrillation, which can be limited by substantial ascertainment bias, while our approach predicts continuous imaging measures of left atrial structure and function that capture the underlying atrial substrate. To our knowledge, this is the first model that uses the 12-lead ECG to predict left atrial structure and function measures and has shown promising findings: our ECG-AI measures are independently associated with downstream cardiovascular complications across several large cohorts.

4. They used human genomic data, is the data used in this study from the same participants? Furthermore, details about genomic data processing and analysis are not well addressed.

The genomic dataset was derived from the sample of UKB participants who with ECGs and genetic data. Participants used to develop the ECG-AI models (*train* and *validate* samples) were excluded from our genetic analyses (Supplementary Fig. 1).

Methods, under "Genome-wide association studies": "In the UKB, 488,377 participants were genotyped for over 800,000 variants using DNA extracted from blood samples collected at the baseline examination[5]. Genotypes were directly called using the UK Biobank Axiom (Affymetrix, Santa Clara, California) and the UK Applied Biosystems Array. Imputation was carried out using the Haplotype Reference Consortium and UK10K+1000 Genomes reference panels. Variants that did not pass quality control checks were excluded from further UKB analyses. We performed Genome-wide Association Studies (GWAS) of the five ECG-AI left atrial traits: LAVmin, LAVmax, LAVImax, LAVImin and LAEF. Individuals with a prior history of AF, a pacemaker or non-European ancestry were excluded. Analyses were performed using a linear mixed model to account for relatedness using the GENESIS package[6]"

5. The biggest concern is the validation and authenticity of results, and their clinical acceptance and translational impact.

As indicated in more detail below, we have revised the manuscript to emphasize the clinical relevance of our ECG-AI models, which were developed in the world's largest imaging cohort (UKB), then externally validated in

two large and diverse NHLBI prospective cohort studies (CHS and MESA). Findings were strongest in the validation cohorts: ECG-AI measures of left atrial structure and function were independent cardiovascular risk factors as strongly associated with outcomes as widely studied imaging measures. The robust findings suggest that this low-cost and widely available technology (conventional 12-lead ECG combined with fully trained prediction models) is well-suited to clinical implementation, and perhaps even population-wide screening programs. The ECG-AI models did not require retraining or significant pre-processing, and performed similarly in different populations. Because of these features, colleagues at two large health systems are working on applying these models to millions of digitized ECGs for evaluation of potential clinical use cases.

6. Some minor comments, data analysis can be better planned and presented. Better choice of figures can be made for clear presentation and interpretation. Method's reproducibility and transparency can be better explained.

Prompted by the helpful comments from all reviewers, we have substantially revised the manuscript with extensive changes to the Methods and additional Figures. These revisions have improved the clarity and readability of this manuscript.

Reviewer #1 (Remarks on code availability):

There must be guidelines in accessing data, source code, and execution. In current form, ReadMe is not clear and self explanatory for the readers to exercise.

We agree that the original README lacked sufficient structure and guidance for users unfamiliar with the codebase and have substantially revised the README to improve clarity and usability. The updated documentation now includes: (1) explicit installation instructions with dependencies listed for both standard and Docker-based workflows; (2) a step-by-step Quick Start Guide that walks users from model download through prediction; (3) clear documentation of input format requirements, including dimensions, lead order, and units; (4) a new preprocessing section describing how to convert raw 10-second ECG recordings into the required median beat format using the provided process_ecg_to_median_beat.py script; (5) explicit documentation of output units, confirming that LAVmin and LAVmax are in milliliters (mL) and LAEF is in percent (%); and (6) a troubleshooting section addressing common errors. The revised README is available in the GitHub repository. These changes should make it considerably easier for users to apply the model.

Reviewer #2 (Remarks to the Author):

1. The model is trained on cardiac MRI labels generated by an automated segmentation algorithm rather than on manually annotated ground truth. The authors do not quantify segmentation error, assess uncertainty propagation, or validate model performance against a manually labeled subset. Without this, it is difficult to determine whether the ECG model is learning true left atrial structure or reproducing artifacts of the segmentation pipeline.

This helpful comment prompted us to address these issues with more detail and clarity in the manuscript. The CMR labels used in this study were generated by an automated segmentation algorithm that was validated against a manually labeled subset and underwent additional quality control to evaluate segmentation error and minimize uncertainty. All left atrial measurements were derived using a fully automated segmentation pipeline developed by Bai et al. in the UKB. This pipeline was trained using a dataset of manually annotated images from 5,008 participants. DICOM images and annotations were quality controlled to ensure they covered both the end-diastolic and end-systolic frames. The fully convolutional network was employed to learn the image features through a series of convolutions, ultimately resulting in automated image segmentation. The accuracy of these automated measures was evaluated both by various technical metrics and qualitatively by clinical experts (Bai et al). For example, when the Dice metric was evaluated on 600 subjects, the mean Dice metric was 0.93 for the LA on the 2-chamber view, 0.95 for the LA on the 4-chamber view, demonstrating a high degree of accuracy. We applied this algorithm to our sample of UKB CMR images, then performed additional quality control focused on the outliers to improve the reliability of the automated measurements. To identify segmentation errors, a cardiac imaging fellowship-trained cardiologist and coauthor visually inspected the 10 highest and 10 lowest measurements for each trait in 1-mL increments for LA volumes and 1-percent increments for LA ejection

fraction. Observed errors for the lowest LA min and LA max and the highest LA EF values included improper identification or foreshortening of the left atrium; no errors were observed for the highest LA min and LA max values and the lowest LA EF values. After a multidisciplinary discussion weighing the competing risks and benefits of QC thresholds that are too high or too low, we set exclusion thresholds for each LA measure at values where >50% of visually inspected images included segmentation or imaging-plane errors. Based on this protocol, CMR studies with LA minimal volume <= 8 mL, LA maximal volume <= 24 mL, and LA ejection fraction >86% were excluded, representing 3.1% of cardiac imaging studies. In addition, we randomly sampled and visually inspected 50 measurements for each directly measured trait and observed that 96% (96/100 across both LA volume traits) were accurate. This QC effort involved the visual inspection of images from over 200 participants. These same QC-processed measures were also used in our recently published epidemiologic and genome-wide association study[7].

Despite these QC measures, it is possible (and even likely) that LA measures derived from this automated segmentation pipeline were erroneous for some participants. Measurement error is ubiquitous feature of virtually any clinically relevant measurement, and in this case segmentation error is unlikely to be a major one. The robust findings that replicated in two independent settings strongly suggest that whatever measurement error may be present was not large enough to overwhelm the biologically informative information captured by the ECG-AI models.

Revised Online Methods section on "Cardiac magnetic resonance imaging parameters":
"The accuracy of these automated methods was evaluated quantitatively by various technical metrics (Dice metric, contour distance metrics, and Hausdorff distance) and qualitatively by clinical experts[8]. For example, when the Dice metric was evaluated on 600 subjects, the mean Dice metric was 0.93 for the LA on the 2-chamber view, 0.95 for the LA on the 4-chamber view, demonstrating good accuracy.

Left atrial ejection fraction (LAEF) was calculated through the following formula: [(maximal volume – minimal volume)/maximal volume *(100)]. A cardiologist then performed additional systematic quality control focused on the outliers to improve the reliability of the automated measurements. The 10 highest and 10 lowest measurements for each trait were visually inspected to identify segmentation errors. This process was then repeated in 1-mL increments for LA volumes and 1-percentile increments for LAEF. After a multidisciplinary discussion weighing the risks and benefits of over- or under-exclusion, we defined the quality control exclusion threshold as any increment in which ≥50% of the visually inspected images contained segmentation or imaging-plane errors. Based on this protocol, we included the following measures in our analysis: LA minimal volume > 8 mL, LA maximal volume > 24 mL, and LAEF ≤86%. To validate our process, we randomly sampled and visually inspected 50 measurements for each directly measured trait and observed a 96% accuracy rate."

2. The procedures for ECG preprocessing and exclusion are insufficiently documented. The manuscript does not report how many ECGs were removed due to quality issues, arrhythmias, lead reversal, or automated flags. Because the excluded ECGs likely come from individuals with more advanced pathology, this may bias the training population and reduce the model's ability to generalize to sicker patients.

We have added individual counts (and percentages) to the various exclusion criteria in the methods section and corrected Supplementary Fig. 1. We also compared baseline characteristic between excluded and included participants to assess potential selection bias: Quality-related exclusions (poor quality, no interpretation possible, suspect arm reversal, undetermined rhythm, implausible rates; N=2,225 (3.5%)): Mean age 65.1 (SD 7.7) vs. training sample 63.8 (SD 8.2) years, suggesting minimal age-related bias from technical quality exclusions. Arrhythmia or pacemaker presence exclusions (atrial fibrillation/atrial flutter; N=1,251, pacemaker; N=174): Mean age 71.4 (SD 6.5) years, representing older individuals with prevalent disease who were appropriately excluded from a model intended to predict *incident* AF.

Methods: "Quality-related exclusions included: poor-quality data (N=1,483 [2.4%]), no interpretation possible (N=3 [< 0.1%]), suspect arm/limb lead reversal (N=429 [0.7%]), undetermined rhythm (N=265 [0.4%]), and implausible ventricular rates <20 or >140 bpm (N=45 [0.1%]). Clinical exclusions included: atrial fibrillation

(N=1,153 [1.8%]), atrial flutter (N=98 [0.2%]), and pacemaker rhythm (N=174 [0.3%]). Due to potential overlap between categories (e.g., AF with poor quality), the total number of excluded ECGs was N=3230 (5.1%) as detailed in Supplementary Fig. 1. Participants excluded for quality-related reasons had similar baseline characteristics similar to the included population (mean age 65.1±7.7 vs. 63.8±8.2 years), while those excluded for atrial fibrillation/flutter or pacemaker presence were older (71.4±6.5 years), consistent with prevalent disease."

3. The dataset split of 45% train, 5% validation, and 50% test is unconventional and may reduce the effective training data for a high capacity model. The authors do not describe stratification, nor do they address how individuals in the UKBB were separated across splits.

We agree that a larger training set could potentially improve model performance. The 45/5/50 split was chosen to prioritize a large test set for robust epidemiologic analyses of ECG-AI-predicted LA metrics and clinical outcomes. This allocation balanced model development needs with adequate statistical power for downstream association analyses. As we undertake new work on other ECG-AI modelling approaches in the full sample of UKBB imaging participants, which now has longer follow-up accrual and more outcome events, we plan to allocate a larger proportion of the sample to model development.

All splits were performed randomly at the participant level, with each individual appearing in only one dataset. Given the large sample size, random assignment ensured balanced baseline characteristics across splits, which we verified empirically in Supplementary Table 1. Outcomes were evaluated exclusively in the test set and were not used during model training or validation. We have clarified these points in the Methods.

Methods: "ECG-AI models to predict CMR measured LAVmin, LAVmax and LAEF were trained in UKB using a deep convolutional neural network (CNN). We used the median beat waveform as model input because it reduces dimensionality while preserving morphological features relevant to structural cardiac prediction. The median beat approach eliminates beat-to-beat variability and ectopic beats that do not reflect underlying cardiac structure and improves robustness to acquisition noise and device-specific artifacts through signal averaging."

"The UKB sample was randomly divided into 45% training, 5% validation and a 50% hold-out test sets, with each individual contributing data to only one split; this resulted in balanced baseline characteristics across samples (Supplementary Table 1). The large allocation to the test sample was done to provide adequate power for extensive epidemiologic analyses of association with clinical outcomes."

4. The evaluation of the core regression task is incomplete. The model's accuracy is presented using (weak) correlations only. Absolute error metrics such as RMSE or MAE are not reported, and no calibration analyses are shown. Without these, it is unclear whether predicted volumes and ejection fraction values are sufficiently accurate to serve as surrogates for MRI measurements.

We agree that correlation alone does not fully characterize model performance. We have now added error metrics (MAE and RMSE) and included Bland–Altman analyses to assess agreement and systematic bias between ECG-predicted and CMR-measured values. Absolute prediction errors represented 0.67-0.72 standard deviations of the variability in LA measurements. Mean absolute error was 8.6 mL for LAVmin (mean CMR value: 29.0±12.8 mL), 14.8 mL for LAVmax (mean CMR value: 72.5±21.7 mL), and 5.7 percentage points for LAEF (mean CMR value: 61.0±7.9%). Root mean squared errors were 11.5 mL, 19.1 mL, and 7.3 percentage points, respectively. Bland–Altman analyses demonstrated no overall systematic bias (mean differences: <1 mL for volumes, <1% for LAEF). However, proportional bias was evident, with the model tending to underestimate LA volumes at higher values and overestimate at lower values (i.e., regression toward the mean). Despite this limitation, ECG-predicted values retained strong associations with clinical outcomes, as detailed in the main analyses.

Results and Supplementary Figure:
"Absolute prediction errors represented 0.67-0.72 standard deviations of the variability in LA measurements. Mean absolute error in the test set was 8.6 mL for LAVmin (mean CMR value: 29.0±12.8 mL), 14.8 mL for LAVmax (mean CMR value: 72.5±21.7 mL), and 5.7 percentage points for LAEF (mean CMR value:

61.0±7.9%). Root mean squared errors were 11.5 mL, 19.1 mL, and 7.3 percentage points, respectively. These prediction errors are consistent with the observed correlations and indicate the model captured moderate inter-individual variation in LA parameters. Bland–Altman analyses showed no overall systematic bias (mean difference <1 mL for volumes, <1% for LAEF); however, proportional bias was evident, with the model tending to underestimate extreme values (regression toward the mean) (Supplementary Figure 2). "

Supplementary Fig. 2. Bland–Altman analysis of ECG-AI predicted vs CMR measured left atrial parameters

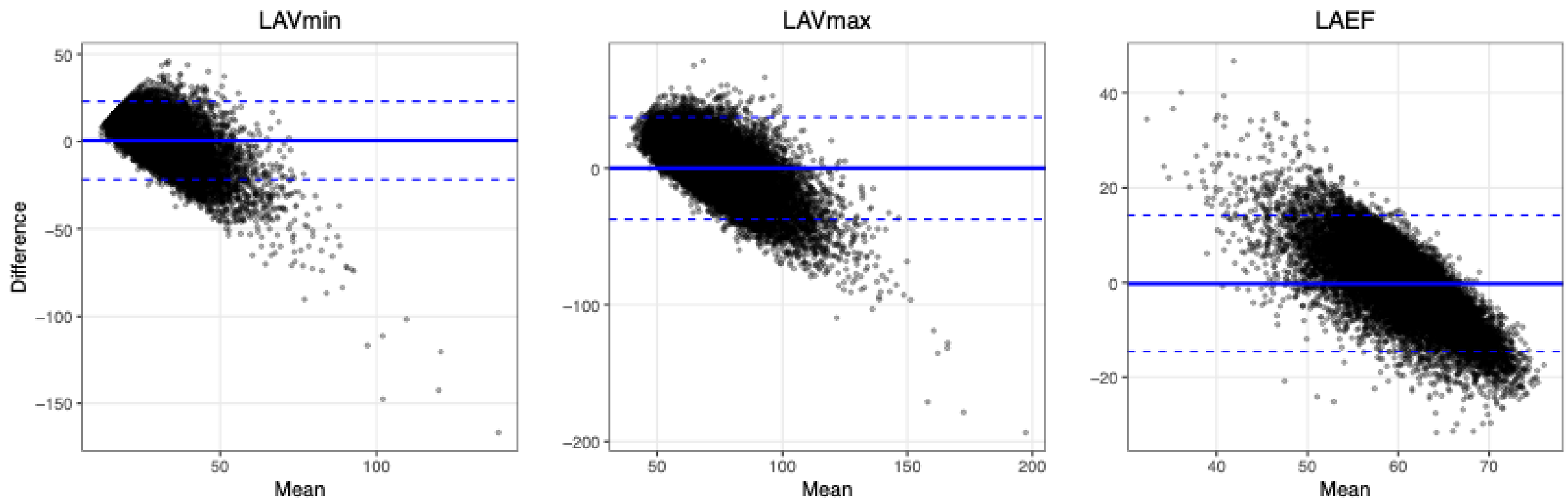

Bland-Altman plots for LAVmin, LAVmax, and LAEF in the UK Biobank test set. The x-axis shows the mean of predicted and measured values; the y-axis shows the difference (predicted minus measured). Solid line: mean difference (bias); dashed lines: 95% limits of agreement (±1.96 SD). Proportional bias is evident, with systematic underestimation at higher values and overestimation at lower values.

5. The outcome modeling framework lacks several important statistical diagnostics. The proportional hazards assumption is not tested, competing risks are not modeled, and multiple hypothesis testing is not addressed despite numerous comparisons across cohorts, outcomes, and left atrial metrics. These omissions weaken the validity of the reported associations.

Proportional hazards assumption:

We tested the proportional hazards (PH) assumption for all primary exposure variables using Schoenfeld residuals. In UKB, the PH assumption was satisfied for all outcomes (all p>0.05). In MESA and CHS, which have substantially longer follow-up (up to 26 years), PH violations were observed for some measures in the full follow-up period (new Supplementary Table 5). To address this, we performed sensitivity analyses restricting follow-up to 5 years. With 5-year restricted follow-up, PH violations were largely resolved, and associations were systematically stronger across all outcomes. For example, in CHS with 5-year follow-up, ECG-AI LAVImin for incident AF showed HR=1.49 (95% CI 1.39-1.61, $p=3.20\times10^{-28}$, PH test p=0.52) compared to HR=1.41 with full follow-up (PH test p=0.13). These findings indicate our reported associations are conservative estimates of a shorter duration of risk (e.g. 5 years) because of the long duration of lower relevance of ECG-based risk measures 20 years into the future.

Methods: "Proportional hazards assumptions were tested using Schoenfeld residuals for all exposure variables. In MESA and CHS, where some measures showed evidence of non-proportionality over extended follow-up, sensitivity analyses restricted to 5-year follow-up were performed (Supplementary Table 5).

Results: "In MESA and CHS, some measures showed evidence of non-proportionality over the extended follow-up period; sensitivity analyses restricted to 5-year follow-up showed stronger associations and largely resolved the proportional hazards violations (Supplementary Table 5)"

Discussion: "In MESA and CHS, some measures showed violations of the proportional hazards assumption over extended follow-up; however, sensitivity analyses restricted to 5 years showed substantial resolution of violations and stronger associations, indicating our primary results provide conservative estimates for clinically relevant time horizons."

Competing risks:
There are two main approaches that can be used to account for competing events – subdistribution hazard models (e.g. Fine and Gray) and cause-specific hazard models (e.g. Cox models with censoring at the time of competing event). When primary interest lies in evaluating etiological associations, the cause-specific hazard approach is not only statistically adequate, but it also has a more relevant interpretation, i.e. the estimated HRs are 'among those patients who did not (yet) experience the event of interest or a competing event'[9]. Therefore, we used the cause-specific hazard function to handle the competing event of death. The limitations of and misuse of subdistribution hazards have been widely reported in the literature.

Multiple testing:
We present all p-values without formal multiple testing correction. Our primary scientific interest is comparing effect sizes and consistency of associations across cohorts rather than binary hypothesis testing. Our analyses tested pre-specified, biologically motivated hypotheses about ECG-AI measures and cardiovascular outcomes, with specific predictions about cardioembolic stroke. The robustness of findings is demonstrated by consistent effect sizes and directions across three independent cohorts, which provides stronger evidence than statistical significance in a single cohort. Many associations show large effect sizes with very small p-values (e.g., $p<10^{-20}$) that would remain highly significant under any reasonable multiple testing framework. While we present comprehensive analyses across multiple outcomes and measures, our primary claims focus on the strongest and most consistent associations (AF, cardioembolic stroke, heart failure). For transparency, all p-values are reported explicitly in tables, allowing readers to apply their own multiple testing adjustments as appropriate.

Methods: "All P values are reported without adjustment for multiple testing. Our primary scientific interest is comparing effect sizes and consistency of associations across cohorts rather than binary hypothesis testing, with robustness demonstrated by consistent directions and magnitudes of associations across three independent cohorts."

6. The subclinical atrial fibrillation screening experiment is limited by a six year gap between the baseline ECG and the 14 day monitor. Structural remodeling and incident AFib may have occurred during this period, making the analysis unsuitable for evaluating near term screening potential. Additionally, only a subset of participants underwent monitoring, but the authors do not analyze potential selection bias.

MESA does not have ECGs closer to the date of the 14-day monitor and does not have any imaging measures or outcomes data available for us to review during this period. This is a limitation of contemporary large-scale cardiac monitoring studies conducted in population settings, which are few in number, particular in population settings rather than highly selected clinical populations. It is because of the long interval between the "screening" ECG and cardiac monitoring that these analyses yield a very conservative estimate of the utility of using our ECG-AI models a screening tool, which performed moderately well. These findings are relevant and informative, and at the suggestion of this reviewer we have now called this "exploratory".

Introduction: "In an exploratory analysis of individuals who underwent 14-day cardiac monitoring six years after a screening 12-lead ECG, our ECG-AI measures predicted which individuals had cardiac monitor-detected AF with greater accuracy than an existing AF risk prediction tool and serum biomarker NT-proBNP."

Results section header: "*Screening for AF with 14-day cardiac monitoring: an exploratory analysis*"

Discussion: "Moreover, compared to a clinical risk prediction tool, our ECG-AI improved the 5-year prediction of AF and cardioembolic stroke, the hallmark complication of AF, and exploratory analyses found that ECG-AI identified nearly half of individuals with cardiac monitoring-detected.

…

In the exploratory analysis of AF detection with 14-day cardiac monitoring, ECG-AI identified high-risk individuals with greater accuracy than both clinical risk factors and the cardiovascular biomarker NT-proBNP, suggesting that

this new approach should be evaluated as a potential targeted screening strategy for AF in a more definitive study. "

7. The harmonization of ECG data across devices, acquisition systems, and cohorts is not adequately described. The authors mention voltage rescaling but do not address any other domain shift issues. Without explicit analyses, claims of transportability remain tentative.

We thank the reviewer for the opportunity to clarify this point. We used median beats rather than raw ECG waveforms as model input, which provides inherent robustness to technical variation across acquisition systems. The median beat approach reduces sensitivity to baseline wander, high-frequency noise, and beat-to-beat variability that often differ across ECG devices and acquisition protocols. Beyond voltage rescaling, no additional preprocessing, filtering, or harmonization was performed. This same minimal preprocessing pipeline was applied identically to all cohorts (UK Biobank training data and both external validation cohorts) without cohort-specific adjustments.

Methods: "Beyond voltage rescaling and extraction of median beats, no additional preprocessing, filtering, baseline correction, or harmonization was performed. This minimal preprocessing pipeline was applied identically to all cohorts without cohort-specific adjustments or model retraining."

Discussion: "While our minimal preprocessing approach (median beats and voltage rescaling) demonstrated transportability across the ECG acquisition systems in our study, whether additional harmonization would be needed for substantially different acquisition protocols remains to be determined."

Beyond clarifying our preprocessing approach (above), we have performed explicit learned representation analysis across all three outcomes (new Supplemental Fig. 4, Methods section, Results text and added limitation). t-SNE visualization showed UKB and MESA overlap extensively despite being independent cohorts, supporting transportability when populations are similar. However, CHS formed predominantly distinct cluster with scattered intermixing. Linear regression confirmed CHS participants occupied different embedding positions than UKB/MESA after accounting for age (F-test p<0.0001). Age-matched analysis (70-75 years) quantified this separation: CHS distance from the UKB/MESA centroid (37.6 units) was 3.8-fold greater than the separation between UKB age groups 10 years apart (9.9 units). This likely reflects era-specific acquisition differences, selection bias, and secular trends, factors confounded with age in CHS. The UKB/MESA overlap demonstrates transportability across contemporary cohorts with minimal preprocessing. Without labeled LA measurements in CHS/MESA, we cannot directly validate prediction accuracy in these cohorts. We have added this limitation to the Discussion and revised claims about transportability accordingly.

Results: "To better understand model transportability across cohorts, we analyzed learned representations from the model's penultimate layer. t-SNE visualization of learned representations across all three prediction models showed CHS participants occupying predominantly distinct regions with scattered intermixing, while UKB and MESA overlapped extensively (Supplementary Fig. 4). Linear regression confirmed CHS participants occupied different embedding positions than UKB/MESA after accounting for age (p<0.0001). Age-matched analysis (70-75 years) revealed CHS remained distinctly separated from UKB/MESA (distance: 37.6 units vs 9.9 units between UKB age groups 10 years apart), indicating cohort-specific factors beyond age alone. Without labeled LA measurements in CHS, prediction accuracy cannot be directly assessed in this cohort."

Discussion, limitations: "Learned representation analysis revealed CHS-specific embedding patterns that exceeded age-related variation, likely reflecting era-specific differences in acquisition protocols, selection bias, and secular trends; without labeled LA measurements in CHS, prediction accuracy in this cohort could not be validated."

New Methods section:
Learned representation analysis

Learned representations were extracted from the penultimate dense layer of ECG deep learning models.

Embeddings from the top 5 ensembled models per outcome were concatenated along the feature dimension. Two-dimensional t-SNE embeddings were computed using perplexity=200, random_state=42, and max_iter=1000. Side-by-side visualizations showed cohort membership and age bins (<65, 65-75, 75+ years).
To quantify whether CHS differed from UKB/MESA beyond age alone, we performed linear regression of the first t-SNE component on age and CHS indicator (CHS vs non-CHS). An F-test compared the age-only model to the age + CHS model to assess whether adding cohort membership significantly improved fit.
For age-matched analysis, participants aged 70-75 years were selected from each cohort. Centroids were computed for UKB, MESA, and the combined UKB/MESA group. The Euclidean distance from each CHS participant to the UKB/MESA centroid was calculated and compared to the distance between UKB age groups 10 years apart (60-70 vs 70-80 years).

Supplementary Fig. 4. Learned representation analysis

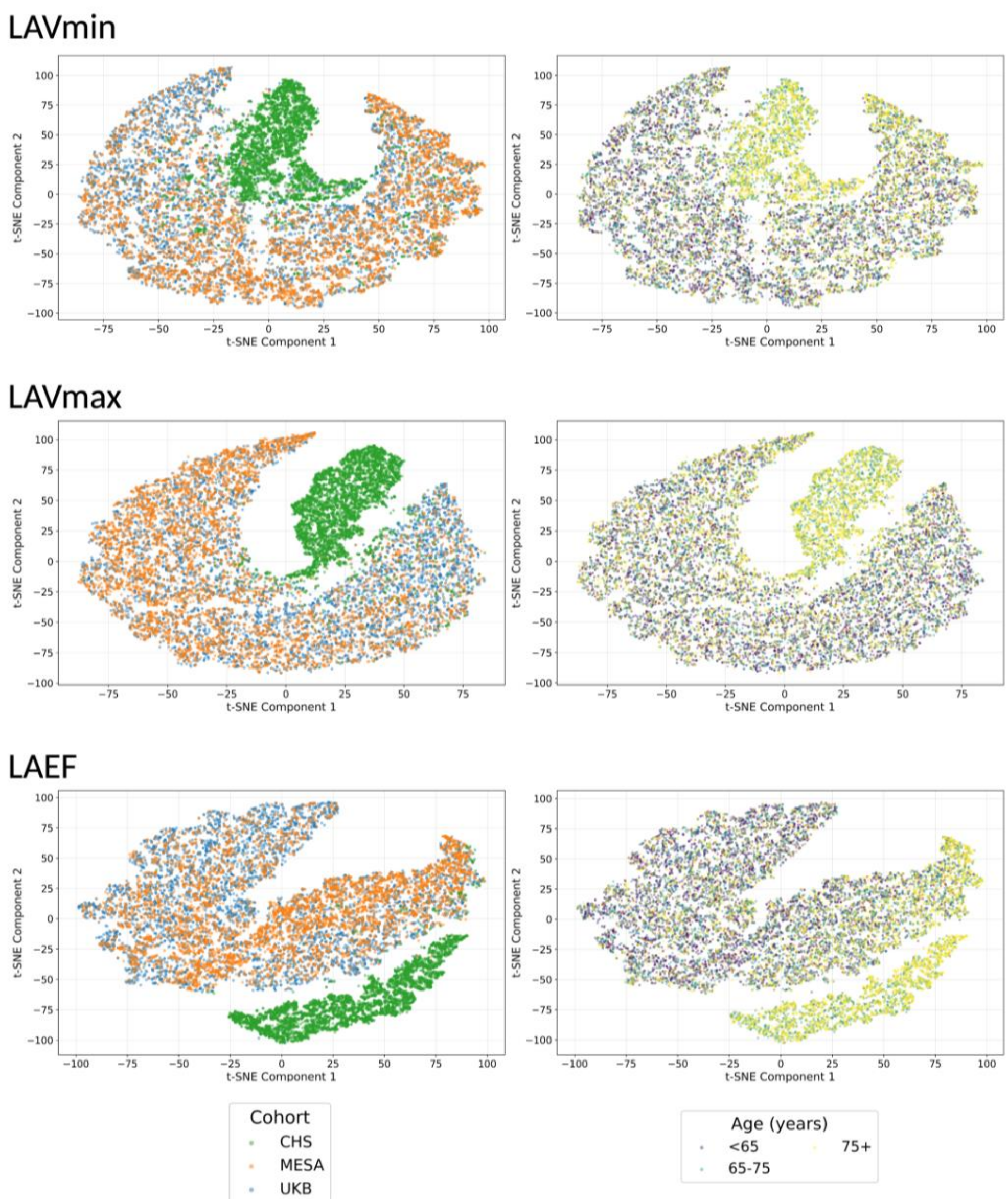

t-SNE visualization of learned representations from ensemble models for LAVmin, LAVmax and LAEF. Left panels are colored by cohort: UKB (blue), MESA (orange), and CHS (green). Right panels show the same embeddings colored by age: <65 years (purple), 65-75 years (blue), and >75 years (yellow). UKB and MESA participants overlap extensively despite being independent cohorts with different acquisition systems, demonstrating transportability across populations. CHS participants occupy predominantly distinct regions with scattered intermixing, which aligns with their older age distribution (mean age 72±5 years vs 55±8 years UKB, 62±10 years

MESA). Age-matched analysis (70-75 years) revealed CHS remained distinctly separated from UKB/MESA (mean distance: 37.6 units vs 9.9 units between UKB age groups 10 years apart), indicating cohort-specific factors beyond age alone.

8. I'm not entirely sold on the utilization of one "median" beat. What exactly is the gain here?

Contemporary acquisition systems (including the GE platforms used in this study) routinely compute a representative median (or averaged) beat after beat alignment and artifact rejection, and this median complex is the basis for all guideline-recommended interval and amplitude measurements. International standards for digital ECG acquisition and reporting (e.g., AHA/ACCF/HRS Recommendations for the Standardization and Interpretation of the Electrocardiogram, Part I: The Electrocardiogram and Its Technology, Circulation, 2007 and IEC/ISO standards (e.g., ISO 41064 and ISO 22077-2)) explicitly assume such vendor-implemented beat alignment, scaling harmonization, and formation of a representative complex. This representative complex or 'median beat' approach reduces dimensionality while preserving morphological information. It also improves robustness to acquisition noise, artifacts and beat-to-beat variability. This in turn facilitates model transportability across different ECG systems, as demonstrated by our external validation results that allowed transporting the model to external datasets without specific preprocessing alignment or fine-tuning. In the results section we have clarified that these data are produced from the acquisition vendor data. Also, in the methods added specific information on the origin or the data and the source software.

Methods, ECG-AI model: "We used the median beat waveform as model input because it reduces dimensionality while preserving morphological features relevant to structural cardiac prediction. The median beat approach eliminates beat-to-beat variability and ectopic beats that do not reflect underlying cardiac structure and improves robustness to acquisition noise and device-specific artifacts through signal averaging."

Methods, Electrocardiograms: "In UKB, ECG waveforms were extracted for all 62,926 subjects with resting digital ECGs measured at the Instance 2 visit that were available for download as of Oct 8th, 2023. ECGs were acquired using CASE® CardioSoft® Version 6 (GE Healthcare). The CardioSoft calculated median beat waveform from ECGs sampled at 500 Hz in each lead were extracted as Extensible Markup Language files.

All ECGs were visually inspected for technical errors and inadequate quality centrally at the Epidemiological Cardiology Research Center (EPICARE) of Wake Forest University School of Medicine (Winston Salem, NC) using General Electric (GE) 12-SL software (GE, Milwaukee, WI) running under GE MUSE and Magellan Research Work Station. Median beat waveforms were extracted from the GE MUSE report."

Results: "In the training sample, we used the vendor-generated median beat of the eight non-derived leads of the 12-lead ECGs to train several models with a range of hyperparameters to predict LAVmin, LAVmax and LAEF (Figure 1)."

8a. And just to be absolutely certain, can the authors explicitly state whether the beat splitting was done after individual cohort level splitting s.t. each ECG yields exactly one data instance? Furthermore, are we absolutely certain that repeat ECGs from a single person were not used in different cohorts?

We confirm that all splitting was performed at the individual (participant) level prior to any ECG processing. Each participant contributed only one ECG to our study, and no individual appears in more than one split (training, validation, or test). Median beat construction was performed independently for each ECG and the clinical ECG software and does not involve information sharing across records; therefore, no information leakage across cohorts is possible.

9. The manuscript sometimes blends evaluation of prediction accuracy with evaluation of prognostic association. Separating these more cleanly would help readers interpret what the model is and is not demonstrating.

We have revised the Results section to distinguish between three distinct types of evaluation: (1) prediction

accuracy (how well ECG-AI predicts CMR-derived measurements), (2) prognostic associations (how strongly ECG-AI measures associate with clinical outcomes), and (3) clinical prediction model performance (discrimination metrics for risk prediction). We separated external validation into two distinct sections.

"External Validation: Accuracy and Representations" now immediately follows the "ECG-AI model" section and focuses exclusively on correlations between ECG-AI predictions and CMR measurements in MESA and CHS, including a new learned representation analysis.
"*External validation of outcome associations: MESA and CHS*" presents hazard ratios for clinical outcomes separately, with an opening sentence that explicitly bridges the two types of evaluation: "ECG-AI associations with cardiovascular outcomes...were similar or greater in magnitude."
Clarified the "Prediction evaluation" section by changing the section heading and adding an introductory sentence that explicitly distinguishes risk prediction model performance from the prognostic associations presented in earlier sections:
"Clinical prediction models for AF and cardioembolic stroke"
Beyond evaluating prognostic associations, we assessed whether ECG-AI measures could improve clinical risk prediction when incorporated into prediction models."

10. The handling of missing clinical covariates in survival models is not described. Whether the authors performed complete case analysis, imputation, or indicator modeling is unclear. This is especially relevant for proBNP, where missingness is common. If it's not common, we're likely overselecting from one cohort of patients.

We have clarified the missing data handling in the Methods section. A complete case analysis was performed for primary survival models given low missingness for most covariates (0.2% in MESA, 2.6% in CHS). The exception was LVEF, used only in heart failure models, where missingness was higher (26% in MESA, 12% in CHS), reflecting a pattern common in prospective NHLBI cohorts where imaging and biomarker assessments were performed in selected subsamples rather than the full cohort.

To address potential bias, we performed a sensitivity analysis using multiple imputation by chained equations (MICE) for the heart failure models. In MESA, HRs were largely stable: ECG-AI LAVImin was unchanged (1.36 in both), LAVImax and LAEF were essentially stable (1.40→1.41 and 0.71→0.72, respectively), and PTFV1 attenuated modestly from 1.26 to 1.19. In CHS, results were virtually identical across all predictors (1.45→1.44, 1.37→1.37, 0.74→0.74, and 1.10→1.11). The direction and statistical significance of all associations were preserved in both cohorts, supporting the robustness of our primary complete case findings (Supplementary Table 17).

NT-proBNP, used only in supplementary sensitivity analyses, had moderate missingness (18% in MESA, 23% in CHS) given its assessment in selected subsamples. We have updated these analyses to use MICE for all analyses adjusting for NT-proBNP (Supplementary Table 11).

Methods: "The LVEF covariate in the heart failure models had higher missingness (26% in MESA and 12% in CHS), reflecting that these measures were obtained in selected subsamples of the cohorts rather than all participants. To assess the potential impact of this missingness, a sensitivity analysis using multiple imputation by chained equations (MICE) was performed; results were consistent with the complete case analysis (Supplementary Table 17).

Sensitivity analyses for associations of LA measures with AF, ischemic stroke, and heart failure were performed as above adding natural log transformed N-terminal pro-B-type natriuretic peptide (NT-proBNP) as a covariate. NT-proBNP assessments were performed in selected subsamples rather than the full cohort and had moderate missingness (18% in MESA and 23% in CHS); MICE was therefore used for these analyses."

11. The model architecture is described at a high level but omits important hyperparameters such as kernel sizes, filter counts, and the

exact search space for the random hyperparameter search.

We have expanded the Methods section to include more detail on the hyperparameter search space and model selection process. The specific hyperparameters selected for each of the five models in each ensemble are provided in Supplementary Table 3.

Methods: “For model training, we performed a random hyperparameter search with 20 configurations per phenotype (LAVmin, LAVmax, LAEF). The hyperparameter search space included: filter counts [40, 80, 120], kernel sizes [6, 12, 24, 48, 96] samples, dense layer sizes of [52, 256] units for layer 1, [4, 8] units for layer 2, and 4 units for layer 3, batch sizes [12, 24], and learning rates sampled from a log-uniform distribution between 10^{-5} and 10^{-2}. All models used the Adam optimizer[10] with 'same' padding, were trained for up to 100 epochs, and employed early stopping[11] with patience set to 3 epochs. Model selection was based on validation set mean squared error. Twenty models were trained and the top five models for each outcome were ensembled using the mean of the predictions. The specific hyperparameters for each model in the final ensembles are provided in Supplementary Table 3.”

12. The Grad CAM visualization is based on extreme examples and is not validated with quantitative perturbation or ablation analyses. Given the limitations of saliency methods for one dimensional signals, I'm not sure the claims related to explainability within the manuscript are justified.

We have now added quantitative ablation experiments to validate the qualitative inspection of extreme cases with Grad-CAM visualizations. Specifically, we performed systematic ablation studies where we progressively removed portions of the ECG signal (0-30% of features) using three strategies: (1) baseline (no ablation), (2) random removal, and (3) saliency-guided removal targeting regions identified by Grad-CAM as most important. As shown in the new Supplementary Fig. 7, saliency-guided ablation caused substantially greater performance degradation than random ablation across all three outcomes. These findings support the claim that Grad-CAM identified ECG regions that are functionally important for model predictions. We have updated the manuscript to include these quantitative analyses and have moderated our claims about explainability to appropriately reflect both the strengths and limitations of saliency methods for time-series data.

Methods: “To assess the functional importance of the regions identified by Grad-CAM, an ablation analysis was performed. Two ablation conditions were compared: (1) saliency-guided ablation, in which the top 5%, 10%, 20%, or 30% of regions ranked by saliency were zeroed out, and (2) random ablation, in which the same fraction of randomly selected regions were zeroed out. Random ablation was repeated five times at each ablation level and results were averaged. The ablated signals were then passed through the model to generate predictions, and performance was compared with the baseline (no ablation) using mean absolute error (MAE). Performance degradation from saliency-guided ablation was compared with that from random ablation at each ablation level to determine whether regions highlighted by Grad-CAM were more critical for model predictions than randomly selected regions.”

Results: “Systematic ablation experiments validated these saliency maps (Supplementary Fig. 7). Removing Grad-CAM high-importance regions caused substantially greater performance degradation than random ablation, confirming functional importance of identified features.”

Discussion: “While ablation validated that Grad-CAM identifies functionally important ECG regions, saliency methods cannot confirm whether features are used in physiologically meaningful ways or capture all relevant patterns.”

Supplementary Fig. 7. Grad-CAM guided ECG feature ablation and prediction performance

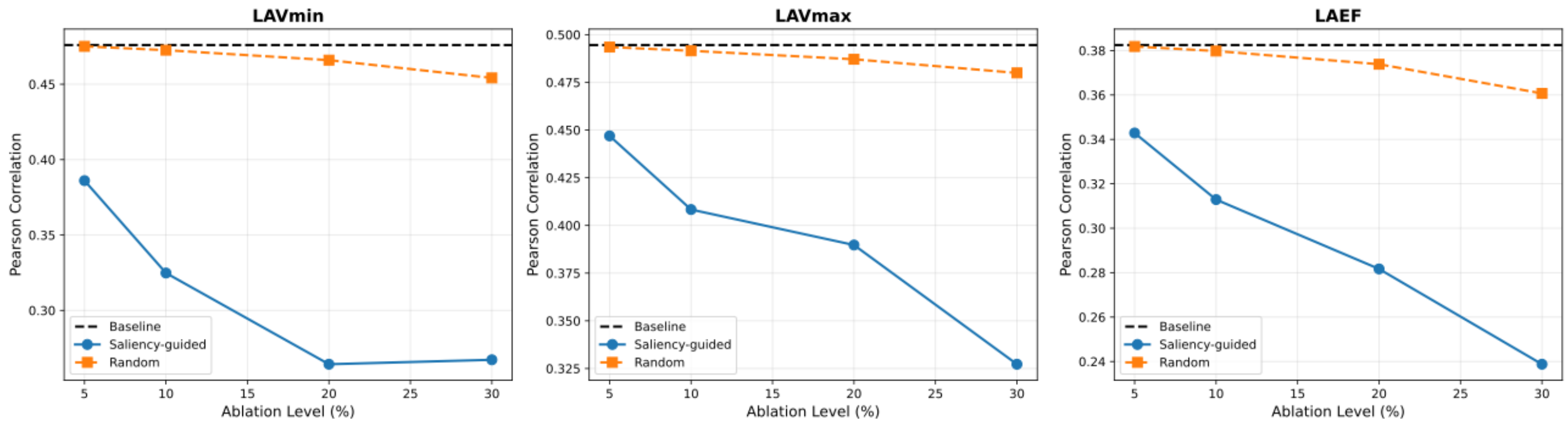

Model performance under progressive ablation of ECG features in UKB. Features were removed at four levels (5%, 10%, 20%, 30%) using saliency-guided ablation (removing Grad-CAM high-importance regions, orange), random ablation (removing random segments, blue), or no ablation (baseline, black). Performance is measured by Pearson correlation on the held-out test set for LAVmin, LAVmax and LAEF. Greater performance degradation with saliency-guided versus random ablation demonstrates that Grad-CAM identifies functionally important ECG regions.

Reviewer #2 (Remarks on code availability):

A key issue affecting both correctness and reproducibility is that the released inference code does not implement the full preprocessing pipeline described in the manuscript. The paper's methods emphasize that each ECG was transformed into a standardized 600 sample median beat derived from ten seconds of raw data, with explicit harmonization of mv scaling across systems. The provided code, however, accepts preprocessed 600x8 NumPy arrays without offering any mechanism to compute the median beat, align cardiac cycles, resample the waveform, or parse raw ECG formats. Consequently, users cannot reproduce the model's inputs from raw ECG data using the released code alone, limiting the reproducibility of the core methodological pipeline.

Additionally, the script does not document the units or scale of the model outputs. The paper indicates that atrial volumes were indexed to body surface area and may have been standardized prior to modeling, yet the inference code emits raw prediction values without specifying whether they correspond to physical volumes, indexed measures, or z-scores. Without explicit post-processing or metadata accompanying the pretrained models, users cannot interpret the resulting LAVmin, LAVmax, or LAEF values in the same units used in the manuscript. This omission directly impairs reproducibility of any downstream analysis based on the predictions.

As such, the provided code isn't very helpful without at least a representative data sample to go alongside it.

We thank the reviewer for this careful and constructive critique. We address each point in turn.

Preprocessing pipeline and reproducibility of model inputs:

As clarified in our prior response, the median beat used as model input is generated by the acquisition system's software (GE MUSE), consistent with vendor-implemented beat alignment and scaling harmonization that follows international ECG standards. Because this preprocessing occurs at the point of acquisition and is not performed in post-hoc analysis software, it was not included in the original inference code.

We recognize, however, that this creates a practical reproducibility barrier for users who wish to start from raw 10-second ECG data. To address this, we have now added a preprocessing module to the GitHub repository that takes a raw 10-second ECG XML file and computes a median beat suitable for model input, replicating the key steps of signal cleaning, R peak detection, PVC filtering, and cardiac cycle alignment.

Table R1 shows association results in MESA using both the GE MUSE-derived and our custom software-derived median beats. Raw predictions of the LA measures here highly correlated (all Pearson correlations > 0.92) and

association results were nearly identical, suggesting the two preprocessing approaches are nearly interchangeable in practice.

Table R1. Comparison of association results in MESA using the median beat used in the manuscript and the median beat derived using the code in our GitHub

		GE MUSE Median Beat Used in Paper		Custom Median Beat Available in our GitHub	
	Event/N	HR (95% CI)	P value	HR (95% CI)	P value
Atrial Fibrillation					
ECG-AI LAVImin	1298/6677	1.34 (1.28-1.41)	2.58E-30	1.34 (1.28-1.41)	1.92E-29
ECG-AI LAVImax	1298/6677	1.34 (1.26-1.42)	5.23E-20	1.33 (1.25-1.42)	2.74E-19
ECG-AI LAEF	1298/6677	0.76 (0.73-0.80)	8.10E-27	0.75 (0.71-0.79)	3.70E-30
Cardioembolic Stroke					
ECG-AI LAVImin	75/5637	1.38 (1.14-1.69)	1.27E-03	1.41 (1.16-1.72)	7.14E-04
ECG-AI LAVImax	75/5637	1.24 (0.96-1.59)	0.10	1.27 (0.99-1.63)	0.06
ECG-AI LAEF	75/5637	0.70 (0.58-0.84)	2.01E-04	0.66 (0.55-0.79)	1.18E-05

Output units and interpretability:

The model outputs LAVmin, LAVmax, and LAEF directly in clinically interpretable units: LAVmin and LAVmax are expressed in milliliters (mL), and LAEF is expressed as a percentage (%). We have updated the inference code and its accompanying documentation (README) to explicitly state the units of each output variable, and have added inline comments to the prediction output section of the script to make this unambiguous for end users.

Reviewer #3

1. Better quantify internal model performance and label quality
In UKB, the authors train "inception-style" 1D CNNs on an 8×600 median-beat representation of the 10-second ECG to predict CMR-derived LAVmin, LAVmax and LAEF, using a 45/5/50% patient-level split. The main internal metric reported is the Pearson correlation in the hold-out test set (r≈0.50 for LAVmin/LAVmax and r≈0.40 for LAEF). While this demonstrates a moderate relationship between ECG-AI and CMR, correlation alone is not sufficient to judge whether the predictions are accurate and unbiased enough to be treated as surrogate LA traits in downstream analyses. The manuscript does not provide MAE/RMSE in the test set, show error behavior at small versus markedly enlarged LA volumes, or present basic bias plots. Given that these continuous ECG-AI traits are later used as exposures in Cox models and in risk prediction, the reader needs a concise description of absolute error and error structure, ideally stratified across the range of LA size and function.

We agree that Pearson correlation alone does not fully characterize model performance. We have now added error metrics evaluated in the test set and included Bland–Altman analyses to assess agreement and systematic bias between ECG-predicted and CMR-measured values. Please see response above to Reviewer #2, comment #4.

In addition, all LA labels come from an automated segmentation pipeline. The authors refer to prior validation, but there is no summary—either from that work or from a quality-control subsample in the present study—of segmentation performance for LA volumes and LAEF in this dataset. A short description of segmentation error and reproducibility would strengthen confidence in the labels and, by extension, in the regression models.

We have updated the section to "Cardiac magnetic resonance imaging parameters" of the Online Methods, as

noted in the response to Reviewer #2, comment #1.

2. Explicitly acknowledge limited power of UK Biobank stroke analyses
In the UKB test set, both CMR-indexed LA traits and their ECG-AI counterparts are related to incident AF, HF, and ischemic stroke over a median of roughly four years, with adjustment for age, sex, and conventional risk factors. The directions and magnitudes of association for CMR LAVImin/LAVmax and LAEF are consistent with prior literature, and ECG-AI traits show broadly similar hazards for AF and, in some models, even stronger associations with HF. These results support the idea that ECG-AI captures much of the risk-relevant signal encoded in LA structure and function.
By contrast, the UKB stroke analysis is clearly underpowered: there are only 71 ischemic stroke events, and hazard ratios are not statistically significant. More definitive conclusions on stroke risk are rightly drawn from MESA and CHS, which have more events and formal subtype adjudication. To avoid over-interpreting the UKB data, it would improve clarity if the authors explicitly stated that the UKB stroke findings are directional only and that substantive inferences about stroke—particularly cardioembolic stroke—are based on the external cohorts.

We thank the reviewer for this important point and agree that the UKB stroke analysis requires clearer acknowledgment of its limited statistical power. The UKB test set included only 71 ischemic stroke events over mean follow-up of 4 years, resulting in wide confidence intervals and non-significant associations for ECG-predicted LA parameters (LAVImin HR=1.16, 95% CI 0.95-1.43, p=0.15). Conclusions about stroke risk are based on MESA and CHS, which provided substantially larger stroke samples with longer follow-up. In MESA (253 ischemic stroke events) and CHS (777 events), ECG-AI LAVImin showed consistent and significant associations with incident stroke (MESA HR=1.16, 95% CI 1.03-1.30, p=0.01; CHS HR=1.22, 95% CI 1.13-1.32, $p=1.88\times10^{-7}$). These cohorts also included formal stroke subtype adjudication, which allowed us to assess cardioembolic stroke specifically. We have revised the Results to explicitly acknowledge UKB stroke findings have limited events, with substantive inferences drawn from external cohorts.

Results: "Associations with ischemic stroke were not statistically significant in the UKB test sample (n=71) and should be interpreted as directional only; substantive stroke inferences are drawn from MESA and CHS."

3. Moderate mechanistic claims that ECG-AI is "beyond imaging"
The external validation in MESA and CHS is a major strength. Applying the UKB-trained models without retraining, the authors show that cross-sectional correlations between ECG-AI and LA imaging traits are lower than in UKB (r≈0.30–0.34), yet associations with AF, HF and ischemic/cardioembolic stroke remain robust and often similar in magnitude to those in UKB after multivariable adjustment. This pattern—attenuated correlation with imaging but preserved outcome association—supports the robustness of ECG-AI as a risk marker across cohorts with different imaging modalities and protocols.
However, the manuscript currently tends to interpret these findings as evidence that ECG-AI captures information "beyond imaging." While this is possible, it is not required by the data: differences in modality (CMR vs echocardiography), acquisition and measurement error between cohorts can readily explain the drop in cross-sectional correlation, and outcome associations reflect not only LA structure but also broader risk profiles and surveillance intensity. I would suggest using more conservative wording that emphasizes ECG-AI as a transportable risk marker which performs comparably to, and in some settings as well as or modestly better than, imaging-based measures, without implying a strong mechanistic claim that it biologically surpasses imaging.

We agree that the pattern of attenuated cross-sectional correlations with imaging (r≈0.30-0.34 in MESA/CHS vs r≈0.40-0.50 in UKB) alongside preserved prognostic associations does not necessarily indicate that ECG-AI captures fundamentally different biological information than imaging. As the reviewer notes, the reduced imaging correlations can be explained by differences in imaging modalities (CMR in UKB/MESA vs echocardiography in CHS), acquisition protocols, and measurement error between cohorts.

We have revised the manuscript to adopt more conservative language, emphasizing ECG-AI as a robust, transportable risk marker with performance comparable to imaging-based measures across diverse populations, and avoiding strong mechanistic interpretations. We have updated the manuscript accordingly:

Abstract: "ECG-AI measures of atrial cardiopathy were strongly associated with new-onset AF, heart failure, and ischemic stroke after adjustment for clinical risk factors and biomarkers in two external cohorts; with magnitudes comparable to or greater than those for direct imaging measures and clinical risk factors."

Results: "Associations for ECG-AI measures were much stronger than for PTFV1 and were similar to or stronger than associations LA reservoir strain associations for all three outcomes (Table 2)."

Discussion: "Our ECG-AI measures demonstrated prognostic associations with cardiovascular outcomes that were comparable to or, in some cases, stronger than conventional ECG and imaging measures of atrial cardiopathy. Human genetic evidence revealed modest genetic correlation with CMR measures (h^2 =0.34-0.62) and some overlap in genomic loci with AF, suggesting the ECG-AI measures and direct imaging share some but not all biological determinants of cardiovascular risk."

4. Clarify the incremental value over conventional markers and risk scores
The comparative analyses with PTFV1, LA strain and CHARGE-AF are important for clinical positioning. It is plausible and well supported by the data that ECG-AI offers stronger risk associations than PTFV1 and LA strain in MESA and CHS, given that it uses the full waveform rather than a single summary metric. The observation that ECG-AI correlates more strongly with log NT-proBNP than do CMR LA measures, and that adjustment for NT-proBNP attenuates but does not eliminate ECG-AI associations with AF, HF, and stroke, is also informative: it suggests that the model integrates a mix of LA and global hemodynamics/load information rather than representing a purely structural LA marker.
For the 5-year prediction models, ECG-AI alone reaches similar AUROC to CHARGE-AF and slightly higher AUPRC and adding ECG-AI to CHARGE-AF yields small but statistically significant improvements in discrimination (ΔAUROC on the order of 0.02–0.04). The analysis itself is well designed. However, expressions such as "outperformed clinical risk scores" are stronger than the effect sizes support. Without calibration plots, Brier scores, reclassification metrics (e.g., NRI/IDI) or decision-curve analyses, it is difficult to judge whether these improvements would meaningfully change clinical decisions. I would recommend moderating the language and, if feasible, adding at least a brief calibration and/or reclassification assessment to quantify the incremental value of ECG-AI over established clinical scores.

We agree that several phrases in the original manuscript overstated the magnitude of improvement over established clinical risk scores, and we have revised the text throughout to better reflect the observed effect sizes. We have also now included decision curve analyses to assess clinical utility beyond discrimination metrics. For atrial fibrillation, the ECG-AI and combined ECG-AI + CHARGE-AF models demonstrated modest but consistent improvements in net benefit across clinically relevant risk thresholds. These analyses support the potential value of ECG-AI for guiding decisions about extended rhythm monitoring. For cardioembolic stroke, net benefit differences were minimal, consistent with its role as a specificity outcome rather than a direct intervention target. We have revised the manuscript to more accurately reflect the observed effect sizes, stating that ECG-AI performs comparably to CHARGE-AF in discrimination, improves precision-recall characteristics, and yields statistically significant but modest improvements when added to clinical risk scores.

Results:
"For 5-year AF prediction (Figure 2, Supplementary Table 11), the ECG-AI model performed similarly to the CHARGE-AF model in terms of area under the received operating characteristic curve (AUROC, 0.75 vs 0.74) but demonstrated improved precision-recall characteristics (AUPRC, 0.23 vs 0.18). Adding ECG-AI to CHARGE-AF resulted in statistically significant but modest improvements in discrimination (Δ AUROC 0.024, 95% CI 0.017-0.034, P < 10E-4) and AUPRC (Δ AUPRC 0.046, 95% CI 0.030-0.063, P < 10E-4). Decision curve analysis showed that ECG-AI and the combined model provided greater net benefit than CHARGE-AF across clinically relevant risk thresholds (Supplementary Fig. 5).

For the prediction of cardioembolic stroke, ECG-AI improved discrimination compared with CHARGE-AF (AUROC 0.82 vs 0.80; AUPRC 0.033 vs 0.026), and the addition of ECG-AI to CHARGE-AF improved prediction further (Δ AUROC 0.037, 95% CI 0.008-0.066, P=0.012). Net benefit differences for cardioembolic stroke were minimal. When categorizing risk predictions into tertiles, the high-risk ECG-AI group has an OR of 52.8 (95% CI 7.3-382, P = 8.5E-5) compared to the low-risk group."

Supplementary Fig. 5. Decision curve analysis for risk-guided intensive cardiac monitoring

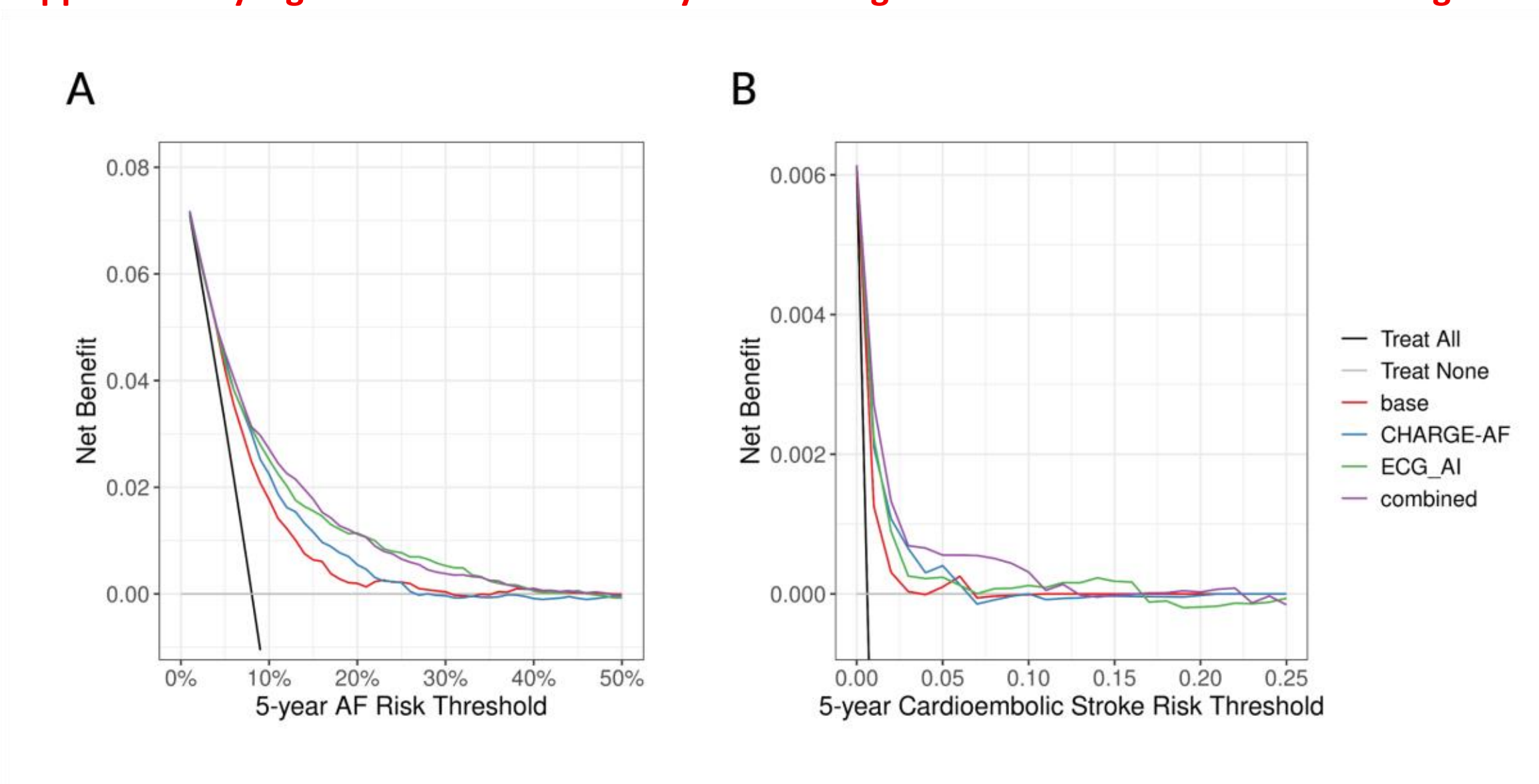

Decision curve analysis comparing net benefit of prediction models across risk thresholds. Panel A shows 5-year atrial fibrillation risk, where elevated risk would inform decisions about extended cardiac monitoring. Panel B shows 5-year cardioembolic stroke risk, included to demonstrate outcome specificity. Net benefit is shown for base model (Age+Sex), CHARGE-AF, ECG-AI, and combined model, alongside treat-all and treat-none strategies. For AF, ECG-AI and the combined model show greater net benefit across clinically relevant thresholds. For cardioembolic stroke, differences between models are minimal.

5. Re-frame the MESA subclinical AF analysis as exploratory

The MESA patch-monitoring sub study addresses a clinically attractive use case: using ECG-AI to enrich for individuals in whom 14-day monitoring is likely to detect AF. The finding that upper-quintile ECG-AI LAVImin groups have higher positive predictive value and capture a larger share of detected AF cases than corresponding CHARGE-AF strata is encouraging and points toward a potential screening application.

Nevertheless, the mean six-year gap between baseline ECG and monitoring, the reliance on a single 14-day monitoring period, and the small number of AF cases (86) substantially limit the strength of inference. As currently written, this section reads close to a practical screening proposal. It would be more appropriate to describe this analysis explicitly as exploratory and hypothesis-generating, and to emphasize that prospective studies with contemporaneous ECG-AI assessment and rhythm monitoring will be needed before ECG-AI can be recommended as a tool to select patients for extended monitoring.

We have reframed this analysis as exploratory and softened the language throughout the manuscript. Please also see response above to Reviewer #2, comment #6.

In summary, this is a well-designed and potentially impactful study that uses high-quality imaging–ECG pairings and large, well-phenotype cohorts to address an important question at the intersection of ECG-based AI, LA remodeling and cardioembolic risk. Its main strengths are the same-day CMR labels, multi-cohort validation and coherent outcome analyses. The main weaknesses are the limited internal characterization of the regression models and the over-interpretation of several exploratory analyses, including some mechanistic claims. With a more complete description of model error and label performance, more cautious and quantitatively grounded comparative claims, and clearer framing of the subclinical AF and genetics components as exploratory.

We appreciate the reviewer's assessment and have directly addressed the identified weaknesses through additional error metrics and bias analyses, more measured comparative language, decision curve analyses to quantify clinical utility, and clearer framing of exploratory components. We believe these revisions appropriately present the study's contributions while acknowledging its limitations.

Reviewer #4

1. Interpretation of the genetic analysis requires caution: The authors claim that their genetic analysis "characterized the biological basis" of the ECG-AI phenotypes. However, the identified loci only partially overlap with known AF loci, and heritability estimates are modest (h^2 0.14–0.17). The PLN variant, while biologically plausible, is already well known as a pleiotropic locus associated with multiple ECG and cardiac traits. The mechanistic link between this variant and LA structure/function is not explored, and thus, the assertion that the GWAS elucidates the biology of AI-derived phenotypes is overstated. The authors should clearly delineate that these findings are preliminary and largely hypothesis-generating.

We agree that our original language overstated the degree to which the GWAS "characterized the biological basis" of ECG-AI phenotypes, and we have revised the manuscript accordingly. The primary purpose of our genetic analyses was to serve as a validity check on the ECG-AI predictions rather than to provide comprehensive mechanistic insight. Because ECG signals are excellent predictors of general risk factors such as age, sex, and BMI, we sought to establish whether ECG-AI predictions captured cardiac-specific biology beyond these general characteristics. The genetic analyses support this interpretation in two ways. First, the moderate genetic correlations between ECG-AI and CMR measures (h^2=0.34-0.62) indicate that ECG-AI predictions share substantial genetic architecture with directly measured left atrial traits. Second, the identification of the PLN locus, a well-established pleiotropic variant associated with multiple cardiac phenotypes including heart failure and AF, supports cardiac specificity of the ECG-AI predictions. We have revised the Introduction and Discussion to clarify that these genetic findings serve primarily to validate the cardiac specificity of ECG-AI predictions rather than to elucidate detailed biological mechanisms, and that the results are hypothesis-generating rather than definitive:

"We also conducted genetic correlation analyses and model explainability studies to validate the cardiac specificity and explore the biological basis of our model's predictions."

"To assess whether ECG-AI predictions captured cardiac-specific biology beyond general risk factors such as age, sex, and BMI, which ECG signals predict well, we performed genetic analyses. The moderate genetic correlations between ECG-AI and CMR measures (h^2 of 0.47 for LAVImin, 0.34 for LAVImax, and 0.62 for LAEF) suggest that our model captures a substantial portion of the heritable component of left atrial structure and function. However, the genetic correlations between our ECG-AI measures and related risk factors and outcomes differed from directly measured CMR variables. The identification of 12 genetic loci, including the well-characterized PLN variant associated with heart failure and AF, supports the cardiac specificity of our ECG-AI measures. These genetic findings validate that ECG-AI captures biologically meaningful cardiac information and provide a foundation for future mechanistic studies."

2. Limited genetic overlap with AF: Roughly half of the identified loci did not coincide with established AF loci. This suggests that ECG-AI phenotypes capture overlapping but distinct biological pathways, possibly reflecting general atrial or ventricular remodeling rather than AF-specific substrate. The authors should explicitly discuss this distinction.

We have revised the Discussion: "Human genetic evidence revealed modest genetic correlation with CMR measures (h^2 =0.34-0.62) and some overlap in genomic loci with AF, suggesting the ECG-AI measures and direct imaging share some but not all biological determinants of cardiovascular risk."

3. Population ancestry and representativeness: The UK Biobank training set is predominantly of European ancestry and healthier than the general population, and the GWAS was restricted to European participants. The validation cohorts (MESA, CHS) improved ethnic diversity, but not for the genetic analysis, limiting generalizability. This should be acknowledged.

We have revised the Limitations section: "Our GWAS analyses were further restricted to European ancestry participants to minimize population stratification, limiting the generalizability of our genetic findings to other

ancestries. While the validation of our ECG-AI in a large multi-ethnic cohort study demonstrates the tremendous potential for transportability of these findings and models to diverse and heterogeneous populations[12, 13], the genetic architecture of ECG-AI traits in non-European ancestries remains to be characterized."

4. Validation cohort limitations: CHS consists exclusively of adults aged ≥65 years, which restricts applicability to younger populations. The authors should clarify whether predictive performance differed by age or cohort (younger MESA participants vs. older CHS participants).

The reviewer is correct that CHS enrolled exclusively adults aged ≥65, and that this limits direct comparison of predictive performance across the age spectrum. To address this, we performed age-stratified analyses in MESA, which enrolled participants across a broader age range, comparing outcomes in those younger and older than 65 years (new Supplementary Table 6). For atrial fibrillation, ECG-AI associations were robust in both age groups but somewhat stronger in older participants (e.g., LAVImin HR 1.40 vs 1.27 in younger vs older MESA participants), consistent with the stronger associations observed in CHS. For heart failure, associations were strong and comparable across age groups, with, if anything, slightly larger point estimates in younger participants (LAVImin HR 1.46 vs 1.35). For ischemic stroke, event numbers in younger MESA participants were limited (N=100), resulting in wider confidence intervals and non-significant point estimates; associations were directionally consistent but not statistically significant in either age group within MESA alone. These findings suggest that the ECG-AI associations with AF and heart failure are not restricted to older adults, though the relative magnitude of associations may vary modestly by age. The stronger AF associations in older MESA participants and CHS are consistent with a higher burden of atrial remodeling with advancing age. We have added Supplementary Table 6 and revised the Results section to include age-stratified analyses in MESA, which support generalizability of ECG-AI associations across the adult age range while acknowledging that further validation in younger populations would strengthen these conclusions:

"Given that CHS enrolled exclusively adults aged ≥65, we performed age-stratified analyses in MESA to assess whether associations differed by age (Supplementary Table 6). ECG-AI associations with AF and heart failure were robust in both younger (<65 years) and older (≥65 years) MESA participants, with AF associations somewhat stronger in older participants (LAVImin HR 1.40 vs 1.27), consistent with the pattern observed in CHS. Heart failure associations were similarly robust across age groups, with point estimates somewhat larger in younger participants (LAVImin HR 1.46 vs 1.35). Ischemic stroke associations were directionally consistent but not statistically significant in either age group, likely reflecting limited event numbers."

5. Moderate correlation with imaging-derived measures: Correlations between ECG-AI and CMR-derived LA parameters were only moderate (r = 0.4–0.5 in UKB; r = 0.3–0.34 in external cohorts). The authors should comment on how this level of concordance supports mechanistic validity.

The ECG-AI and CMR-derived LA parameters were moderately correlated, which is expected, as these measures capture distinct aspects of atrial function. In our genetic analyses, we also observed moderate levels of genetic correlations between the ECG-AI measures and CMR measures. CMR provides a direct anatomic and functional assessment, while the ECG reflects atrial depolarization and repolarization, and captures other downstream aspects of remodeling, loading conditions, and conduction. Accordingly, we would not expect high levels of correlation. However, with only moderate levels of correlation, our study shows that ECG-AI captures enough biologic information to be clinically relevant, supporting its role as a screening tool.

Discussion: "CMR provides a direct anatomic and functional assessment, while the ECG reflects atrial depolarization and repolarization, and captures other downstream aspects of remodeling, loading conditions, and conduction. Although correlations are only moderate, ECG-AI has strong associations with clinical outcomes, and captures biologically meaningful information for cardiovascular risk stratification."

6. Incremental predictive value over existing tools: Although ECG-AI modestly improved discrimination over CHARGE-AF

(ΔAUC ≈ 0.02), the clinical implications of this incremental gain are unclear. Reporting net reclassification improvement or decision-curve analysis would help quantify added value.

We thank the reviewer for this important comment regarding the clinical implications of the incremental discrimination improvements we observed. We agree that discrimination metrics alone are insufficient to establish clinical utility. To address this concern, we have now included decision curve analyses to quantify the added value of ECG-AI beyond traditional discrimination metrics. For atrial fibrillation, the ECG-AI and combined ECG-AI + CHARGE-AF models demonstrated modest but consistent improvements in net benefit across clinically relevant risk thresholds (new Supplementary Fig. 5). These analyses indicate that ECG-AI provides meaningful clinical value for guiding decisions about extended rhythm monitoring, with greater net benefit than CHARGE-AF across the range of thresholds where monitoring decisions would plausibly be made. For cardioembolic stroke, net benefit differences between models were minimal, which is consistent with its role in our study as a specificity outcome to demonstrate the biological relevance of atrial substrate rather than as a direct target for intervention. Beyond discrimination, ECG-AI consistently improved precision-recall characteristics (AUPRC), which may be particularly relevant in screening contexts where positive predictive value is important. These findings, together with the decision curve analyses, support the potential clinical utility of ECG-AI for risk stratification and screening decisions.

See response to similar comment from Reviewer #3, comment #4, for details response and changes to Results section and Supplementary Fig. 5:

7. Subclinical AF detection: The subclinical AF analysis relied on 14-day monitoring approximately six years after baseline. This limited window may underestimate the true AF incidence, particularly for paroxysmal cases. The authors should acknowledge that longer-term monitoring would provide a more accurate benchmark.

This analysis has been reframed as exploratory throughout the manuscript. Please see response to Reviewer #2, comment #6.

8. Biological interpretation of AI-derived traits: While Grad-CAM analyses showed that predictive regions align with physiologic atrial activity (P-wave, PQ-segment), it remains unclear whether the ECG-AI measures capture atrial remodeling distinct from ventricular or systemic predictors (e.g., BMI, hypertension). This distinction should be further discussed.

We agree the ECG-AI model captures information about atrial remodeling that can be influenced by ventricular loading conditions and systemic predictors. From previously published literature (e.g., PMID: 7768556 and PMID: 19808574), we know that hypertension and other chronic conditions can impact atrial remodeling. Our goal with this manuscript was to determine whether ECG-AI derived predictions of left atrial structure and function can be important risk measures for longitudinal cardiovascular events beyond traditional clinical risk factors. Formally separating the atrial, ventricular, and systemic predictors of the ECG-AI measures would require training separate deep learning models that is beyond the scope of the current study. However, in order to further evaluate the biology behind this, we performed a genome wide association study to evaluate the genetic correlations between the ECG-AI predictions, atrial measures, and established cardiovascular risk measures. These results are outlined on Page 8 and in Figure 3 of the manuscript.

1. Yuan N, Duffy G, Dhruva SS, Oesterle A, Pellegrini CN, Theurer J, Vali M, Heidenreich PA, Keyhani S and Ouyang D. Deep Learning of Electrocardiograms in Sinus Rhythm From US Veterans to Predict Atrial Fibrillation. *JAMA Cardiology*. 2023;8:1131-1139.
2. Attia ZI, Noseworthy PA, Lopez-Jimenez F, Asirvatham SJ, Deshmukh AJ, Gersh BJ, Carter RE, Yao X, Rabinstein AA, Erickson BJ, Kapa S and Friedman PA. An artificial intelligence-enabled

ECG algorithm for the identification of patients with atrial fibrillation during sinus rhythm: a retrospective analysis of outcome prediction. *Lancet*. 2019;394:861-867.
3. Khurshid S, Friedman S, Reeder C, Di Achille P, Diamant N, Singh P, Harrington LX, Wang X, Al-Alusi MA, Sarma G, Foulkes AS, Ellinor PT, Anderson CD, Ho JE, Philippakis AA, Batra P and Lubitz SA. ECG-Based Deep Learning and Clinical Risk Factors to Predict Atrial Fibrillation. *Circulation*. 2022;145:122-133.
4. Raghunath S, Pfeifer JM, Ulloa-Cerna AE, Nemani A, Carbonati T, Jing L, vanMaanen DP, Hartzel DN, Ruhl JA, Lagerman BF, Rocha DB, Stoudt NJ, Schneider G, Johnson KW, Zimmerman N, Leader JB, Kirchner HL, Griessenauer CJ, Hafez A, Good CW, Fornwalt BK and Haggerty CM. Deep Neural Networks Can Predict New-Onset Atrial Fibrillation From the 12-Lead ECG and Help Identify Those at Risk of Atrial Fibrillation-Related Stroke. *Circulation*. 2021;143:1287-1298.
5. Bycroft C, Freeman C, Petkova D, Band G, Elliott LT, Sharp K, Motyer A, Vukcevic D, Delaneau O, O'Connell J, Cortes A, Welsh S, Young A, Effingham M, McVean G, Leslie S, Allen N, Donnelly P and Marchini J. The UK Biobank resource with deep phenotyping and genomic data. *Nature*. 2018;562:203-209.
6. Gogarten SM, Sofer T, Chen H, Yu C, Brody JA, Thornton TA, Rice KM and Conomos MP. Genetic association testing using the GENESIS R/Bioconductor package. *Bioinformatics*. 2019;35:5346-5348.
7. Yogeswaran V, Brody JA, Sitlani CM, Wiggins KL, Prutkin JM, Bis J, Yang E, Shah AM, Akoum N, Ye T and Floyd JS. Right and Left Atrial Dysfunction as Independent Cardiovascular Risk Factors: A UK Biobank Study. *Circ Arrhythm Electrophysiol*. 2026:e014412.
8. Bai W, Sinclair M, Tarroni G, Oktay O, Rajchl M, Vaillant G, Lee AM, Aung N, Lukaschuk E, Sanghvi MM, Zemrak F, Fung K, Paiva JM, Carapella V, Kim YJ, Suzuki H, Kainz B, Matthews PM, Petersen SE, Piechnik SK, Neubauer S, Glocker B and Rueckert D. Automated cardiovascular magnetic resonance image analysis with fully convolutional networks. *J Cardiovasc Magn Reson*. 2018;20:65.
9. Noordzij M, Leffondre K, van Stralen KJ, Zoccali C, Dekker FW and Jager KJ. When do we need competing risks methods for survival analysis in nephrology? *Nephrol Dial Transplant*. 2013;28:2670-7.
10. Kingma DPB, J. Adam. A method for stochastic optimization. *International Conference on Learning Representations*. 2015.
11. Yuan Yao LR, and Andrea Caponnetto. OnEarly Stopping in Gradient Descent Learning. *Constructive Approximation*. 2007;26:289-315.
12. Schoeler T, Speed D, Porcu E, Pirastu N, Pingault JB and Kutalik Z. Participation bias in the UK Biobank distorts genetic associations and downstream analyses. *Nat Hum Behav*. 2023;7:1216-1227.
13. Fry A, Littlejohns TJ, Sudlow C, Doherty N, Adamska L, Sprosen T, Collins R and Allen NE. Comparison of Sociodemographic and Health-Related Characteristics of UK Biobank Participants With Those of the General Population. *Am J Epidemiol*. 2017;186:1026-1034.

REVIEWERS' COMMENTS

We thank the reviewers for their time and constructive feedback. The manuscript was substantially strengthened by incorporating their thoughtful comments.

Reviewer #2 (Remarks to the Author):

Thank you for the thorough and constructive revision. The authors have been responsive to my comments, and the manuscript appears substantially improved / stronger in several important respects.

My main concerns have therefore been addressed. That said, the following points would still benefit from minor revision in the final manuscript.

I. Limitations around label quality should be stated as plainly as possible: although the authors provide supporting information on segmentation performance and quality control, the study still does not directly validate the ECG model against a manually labeled subset generated within the present analysis.

> Our UKB training labels were derived from a previously validated automated CMR segmentation pipeline (Bai et al.; mean Dice 0.93–0.95 vs. manual contours), refined by a cardiologist-led quality-control protocol and a random-sample audit (50 measurements/trait, 96% accuracy). We note, however, that the model was additionally evaluated against an independent, manually-derived reference: in MESA, left atrial volumes were manually traced by an experienced operator using area-length methods (i.e., not the automated pipeline used for training), and our ECG-AI predictions correlated with these manual measures (r = 0.30–0.34; Supplementary Fig. 3), with the manually-measured MESA LA values also showing the expected associations with incident AF, stroke, and heart failure (Supplementary Table 8). This provides validation against human-derived labels in an external cohort, with the lower correlation potentially attributable to methodological differences between the manual (MESA) and automated (UKB) measurement approaches.
>
> We acknowledge that we did not perform a de novo manual re-annotation of the UKB training subset itself, and we have revised the Limitations to state this:
>
> "Training labels from UKB CMR were generated by a previously validated automated segmentation pipeline rather than by de novo manual annotation within this study, and therefore it is possible that residual label noise may attenuate the observed ECG-AI/CMR correlations. Although we did not directly validate ECG-AI measures again physician-derived measures in another subset of UKB population, a high degree of segmentation accuracy is supported by published technical metrics (mean Dice 0.93–0.95), our random-sample audit (96% accuracy), and modest correlations of ECG-AI predictions with physician-traced LA measures in MESA (r=0.30–0.34)."

II. the 14 day monitoring analysis should remain explicitly framed as exploratory, and the possibility of selection bias in the monitored subset should be acknowledged clearly in the text.

> The 14-day cardiac monitoring analysis is framed as exploratory throughout the manuscript, including in the Abstract, the Results subheading ("Screening for AF with 14-day cardiac monitoring: an exploratory analysis"), and the Discussion, and we have retained this framing consistently. We have additionally added explicit language acknowledging the potential for selection bias in the monitored subset: cardiac monitoring was performed at MESA Exam 6, an average of six years after the Exam 5 ECG, and was limited to surviving participants who returned and consented to monitoring (with further exclusions for prevalent AF, insufficient monitoring duration, paced rhythm, or missing covariate data). This subset is therefore likely healthier than the full cohort, and the resulting estimates should be interpreted as hypothesis-generating and validated in a prospective study.
>
> We have updated the Limitations to reflect this:

"Our 14-day cardiac monitoring analysis was exploratory and subject to potential selection bias, as monitoring was performed only in surviving participants who returned for MESA Exam 6 (approximately six years after the index ECG) and met eligibility criteria; these findings require confirmation in a prospective study with monitoring performed shortly after ECG-based risk stratification."

III. Claims regarding transportability and biological interpretation should remain calibrated to the level of direct validation provided, particularly in cohorts where left atrial labels were not available for direct accuracy assessment.

We have made two clarifications. First, we now specify that our evidence for transportability reflects consistency of ECG-AI outcome associations across cohorts. Direct accuracy against measured left atrial parameters was assessable only in UKB and MESA, where labels were available. In CHS, which lacked left atrial labels, prediction accuracy could not be directly assessed (as already noted in the Limitations). Second, we have softened our biological-interpretation language. Because our genetic analyses provide correlative evidence rather than direct mechanism, we now describe these findings as "supporting" rather than "validating" the cardiac specificity of the ECG-AI measures.

We have made these revisions to the Discussion:

"The consistent performance of these ECG-AI measures in two external cohort studies using different ECG acquisition systems and requiring only voltage rescaling without additional model training, calibration, or cohort-specific preprocessing demonstrates the reproducibility and transportability of the ECG-AI outcome associations across diverse populations, though formal fairness assessment remains necessary to ensure responsible implementation."

"These genetic findings support that ECG-AI captures biologically meaningful cardiac information and provide a foundation for future mechanistic studies."
